# Supplementary material for: Out-of-Plane Transport of 1T-TaS2/Graphene-Based van der Waals Heterostructures
Source: ACS Nano. 2021 Jul 6;15(7):11898–907. doi: 10.1021/acsnano.1c03012 (PMC8454993; doi:10.1021/acsnano.1c03012)
Supplement: Supplementary file 1 — nn1c03012_si_001.pdf [file nn1c03012_si_001.pdf]

Supporting Information

Out-of-Plane Transport of 1T-TaS_2/Graphene-Based van der Waals Heterostructures

Carla Boix-Constant,[1] Samuel Mañas-Valero,[1,*] Rosa Córdoba,[1]
José J. Baldoví,[1] Ángel Rubio,[2,3] Eugenio Coronado[1,*]

[1] *Instituto de Ciencia Molecular (ICMol), Universitat de València, Catedrático José Beltrán Martínez n° 2, Paterna 46980, Spain.*

[2] *Max Planck Institute for the Structure and Dynamics of Matter and Center for Free-Electron Laser Science, Luruper Chaussee 149, 22761, Hamburg, Germany.*

[3]*Nano-Bio Spectroscopy Group, Departamento de Física de Materiales, Universidad del País Vasco, 20018 San Sebastian, Spain.*

E-mail: samuel.manas@uv.es, eugenio.coronado@uv.es

The following contents are included:

1. Optical contrast of 1T-TaS_2 thin-layers on SiO_2/Si substrates. 2
   - 1.1. Theoretical framework and discussion. 2
   - 1.2. Optical contrast of a 1T-TaS_2 single layer: SiO_2 thickness, wavelength and numerical aperture dependence. 5
   - 1.3. Optical contrast of a 1T-TaS_2 thin-layers layer (0 – 60 nm): SiO_2 thickness and wavelength dependence (NA = 0). 6
   - 1.4. Optical contrast of 1T-TaS_2 thin-layers layer (0 – 60 nm) on 285 nm SiO_2/Si substrates: numerical aperture and wavelength dependence. 7
   - 1.5. Examples of experimental optical contrast of 1T-TaS_2 thin-layers on 285 nm SiO2/Si substrates with an objective lens of NA = 0.9. 8
2. Comparative of in-plane *vs.* out-of-plane devices. 11
3. Junction geometrical factors, Arrhenius and hopping analysis. 14
   - 3.1. Junction geometrical factors. 15
   - 3.2. Arrhenius analysis. 17
   - 3.3. Hopping analysis. 37
4. Measurements on the FLG/TaS_2/ FLG graphene devices. 55
   - 4.1. Device A. 55
   - 4.2. Device B. 74
   - 4.3. Device C. 78
   - 4.4. Device D. 85
   - 4.5. Device E. 87
   - 4.6. Device F. 89
   - 4.7. Device G. 91
   - 4.8. Device H. 92
   - 4.9. Device I. 93
5. Scanning transmission electron microscopy images. 96
6. Band structure calculations. 98
7. References. 99

1. Optical contrast of 1T-TaS_2 thin-layers on SiO_2/Si substrates.

1.1. Theoretical framework and discussion.

Optical contrast is a widely used technique in the field of 2D materials that allows the fast identification of flakes with different thicknesses,[1,2] being of great importance for air unstable materials, such as 1T-TaS_2 and CrI_3.[3] As it has been reported for graphene,[4] metal halides[1,3] and other transition metal dichalcogenides[1,5,6] –but not for 1T-TaS_2-, optical contrast can be done quantitatively and its results compared with those expected theoretically. In this work we consider thin-layers of 1T-TaS_2 on top of SiO_2/Si substrates. The reflected light intensity (I) depends on the thickness of every layer (d), the refractive index of the different materials involved, ñ (ñ = n – ik, where n and k are the real and the imaginary component of the refractive index, respectively), and the incident wavelength (λ). An analytical expression in normal incidence can be derived from the Fresnel equations.[1,3,4]

$$I_{flake}(\lambda) = \left| \frac{r_{01}e^{i(\phi_1+\phi_2)} + r_{12}e^{-i(\phi_1-\phi_2)} + r_{23}e^{-i(\phi_1+\phi_2)} + r_{01}r_{12}r_{23}e^{i(\phi_1-\phi_2)}}{e^{i(\phi_1+\phi_2)} + r_{01}r_{12}e^{-i(\phi_1+\phi_2)} + r_{12}r_{23}e^{i(\phi_1-\phi_2)}} \right|^2 \quad (S1)$$

$$I_{substrate}(\lambda) = \left| \frac{r_{02} + r_{23}e^{-2i\phi_2}}{1 + r_{02}r_{23}e^{-i\phi_2}} \right|^2 \quad (S2)$$

In the expressions above, the subindices refer to the different media involved (0, 1, 2 and 3 correspond to argon, 1T-TaS_2, SiO_2 and Si, being the last one considered as a semi-infinite layer), r_{ij} is the reflection Fresnel coefficient when the light travels from medium i to medium k, $r_{ij} = \left| (\tilde{n}_i - \tilde{n}_j)/(\tilde{n}_i + \tilde{n}_j) \right|$, and Φ_i is the optical pathway length in the medium i, $\Phi_i = 2\pi\tilde{n}_i d_i/\lambda$.

The optical contrast can be defined as:[1,3]

$$C(\lambda) = \frac{I_{flake} - I_{substrate}}{I_{flake} + I_{substrate}} \quad (S3)$$

From **Equation S3**, the optical contrast can be estimated from both the experimental and theoretical point of view. Experimentally, it can be obtained from optical microscopy images: the I_{flake} value is obtained by selecting a region of interest in an RGB image and averaging its intensities (the same procedure follows for $I_{substrate}$). From the theoretical point of view, the reflected intensities of the flake and the substrate can be estimated using the Fresnel equations as described above. Previously reported refractive indices were employed for Ar,[7] Si[8] and SiO_2.[9] In the present case, we worked inside an argon glovebox; nonetheless, a similar trend in the optical contrast is expected for nitrogen or air environments due to their similarities of the refractive index in the visible range. The only available optical data for 1T-TaS_2 was the dielectric constant (dispersion, ε_1, and absorption, ε_2).[10] The refractive index was then calculated by considering the optical relationships $\varepsilon_1 = n_2 - k_2$ and $\varepsilon_2 = 2nk$.[10]

For a single layer in normal incidence (**Figure S.1**), optimal contrasts are found for a wavelength of 750 nm and SiO_2 thickness of either 100 nm or 380 nm (C ~ - 20 %). For the SiO_2 thickness of 90 nm, 285 nm and 300 nm, some of the most common SiO_2 thickness used in the field of 2D materials,[4,11] the optimum contrast for identifying a monolayer is obtained for 540 nm (C ~ -14 %), 565 nm (C ~ -14 %) and 590 nm (C ~ -15 %), respectively. In general, the optical contrast of single layer 1T-TaS_2 is comparable to other transition metal dichalcogenides like $TaSe_2$ (C ~ -27 % for 265 nm of SiO_2 and $\lambda = 550$ nm)[1] or $NbSe_2$ (C ~ -20 % for 90 nm of SiO2 and $\lambda = 500$ nm)[11]. Once the thickness of SiO_2 is fixed, it is possible to identify thin-layers (those flakes with thicknesses below 10 nm). For this purpose, it must be identified the conditions where the variation of the optical contrast with the 1T-TaS_2 thickness in the range below 10 nm is higher. As can be seen in **Figure S.2**, the optimal curves for the identification of thin-layers exhibit a negative contrast in the thin-layer regime that, later, changes its slope until it tends to saturate. The optimal hot spots are found for 90-100 nm of SiO_2 and 285-300 nm of SiO_2 and a wavelength in the range of 550 - 600 nm. On the

contrary, native silicon oxide (1 – 3 nm) or 150 nm of SiO_2 are not good conditions for the optical identification of thin-flakes at any incident wavelength.

The previous discussion is applicable for a strictly normal incidence of the light. However, it is common to determine the optical contrast by using an optical microscope where the light is focused by an objective lens and, thus, the numerical aperture (NA) of the objective has to be taken into account since the different angle of light incidence modify the optical pathway and affects the interference. The main feature is a diminishment of the magnitude as well as a blue shift of the optical contrast peak as a consequence of the destruction of the optical interference, which can be seen in **Figures S.1** and **Figure S.3**. In order to consider this oblique incidence, it has to be taken into account that for s(p)-polarized light, the reflection coefficient, $\tilde{n}_i$, must be substituted by $\tilde{n}_i \cdot \cos\theta$ ($\tilde{n}_i/\tilde{n}_j \rightarrow \tilde{n}_i\cos\theta_j/ \tilde{n}_j\cos\theta_i$), where θ is the incident angle (for unpolarized light, an average is taken of both contributions), and that Φ_i is $2\pi\tilde{n}_i d_i \cos\theta_i/\lambda$, where θ_i is the refraction angle in the layer i ($\theta_i = \text{asin}((n0/n_i)\cdot\sin\theta)$, as can be derived from Snell's law). Then, for including the NA of the objective, the reflected light intensity has to be calculated by the numerical integration of the intensities over the solid angle of each NA, determined by the angle θ_m. Considering that θ_m = asin(NA) and a Gaussian distribution of the collected light ($W(\theta) = \exp(-2\sin2\theta/\sin2\theta_m)$), equation S1 and S2 are transformed to:[12,13]

$$I_{flake}(\lambda) = 2\pi \int_0^{\theta_m} I_{flake}(\theta) W(\theta) \sin\theta d\theta \quad \text{(S4)}$$

$$I_{substrate}(\lambda) = 2\pi \int_0^{\theta_m} I_{substrate}(\theta) W(\theta) \sin\theta d\theta \quad \text{(S5)}$$

The predicted contrast tendencies are validated experimentally for 285 nm of SiO_2 (**Figure S.4**, **Figure S.5** and **Figure S.6**), observing the maximum contrast for thin-layers in the green region of the electromagnetic spectrum.

1.2. Optical contrast of a 1T-TaS_2 single layer: SiO_2 thickness, wavelength and numerical aperture dependence.

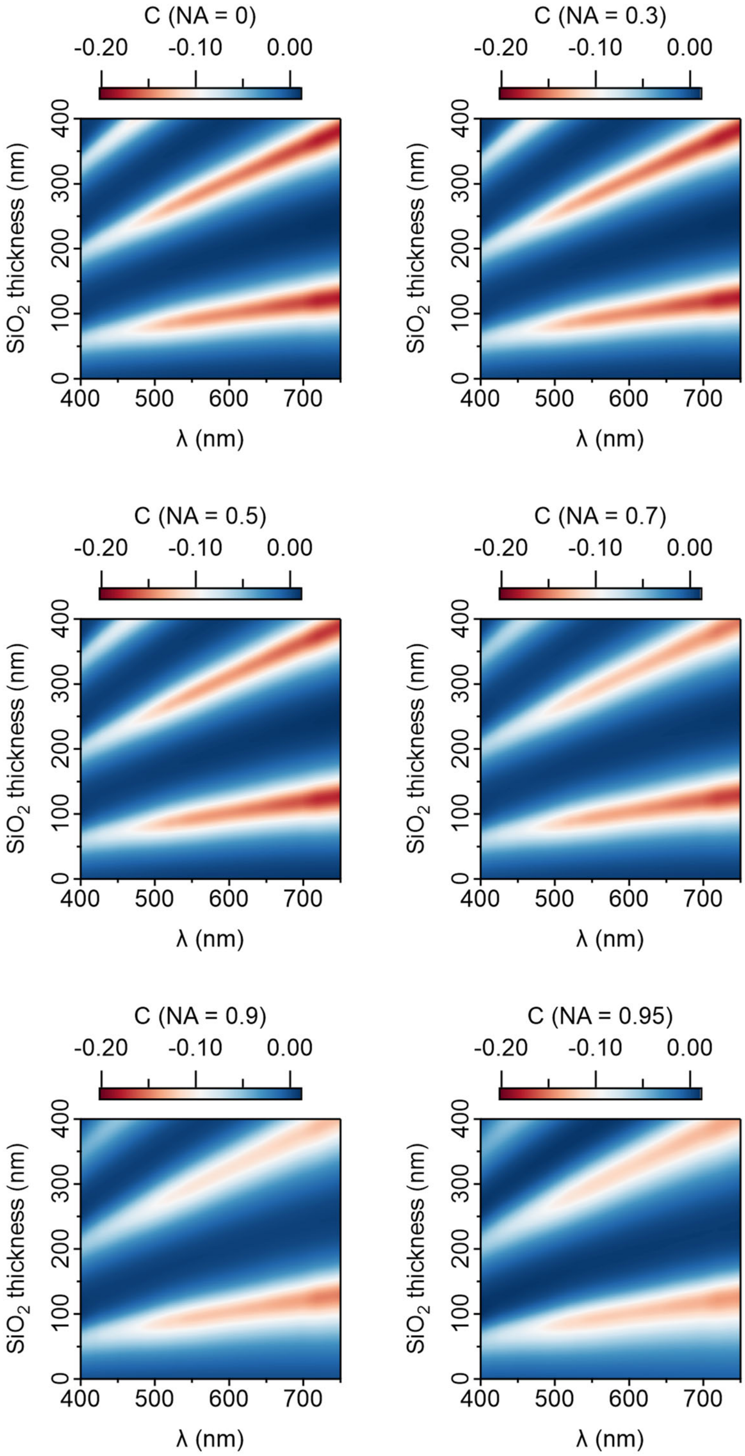

Figure S.1.- Optical contrast of a 1T-TaS_2 monolayer as a function of the wavelength and SiO_2 thickness for different numerical apertures.

1.3. Optical contrast of a 1T-TaS_2 thin-layers layer (0 – 60 nm): SiO_2 thickness and wavelength dependence (NA = 0).

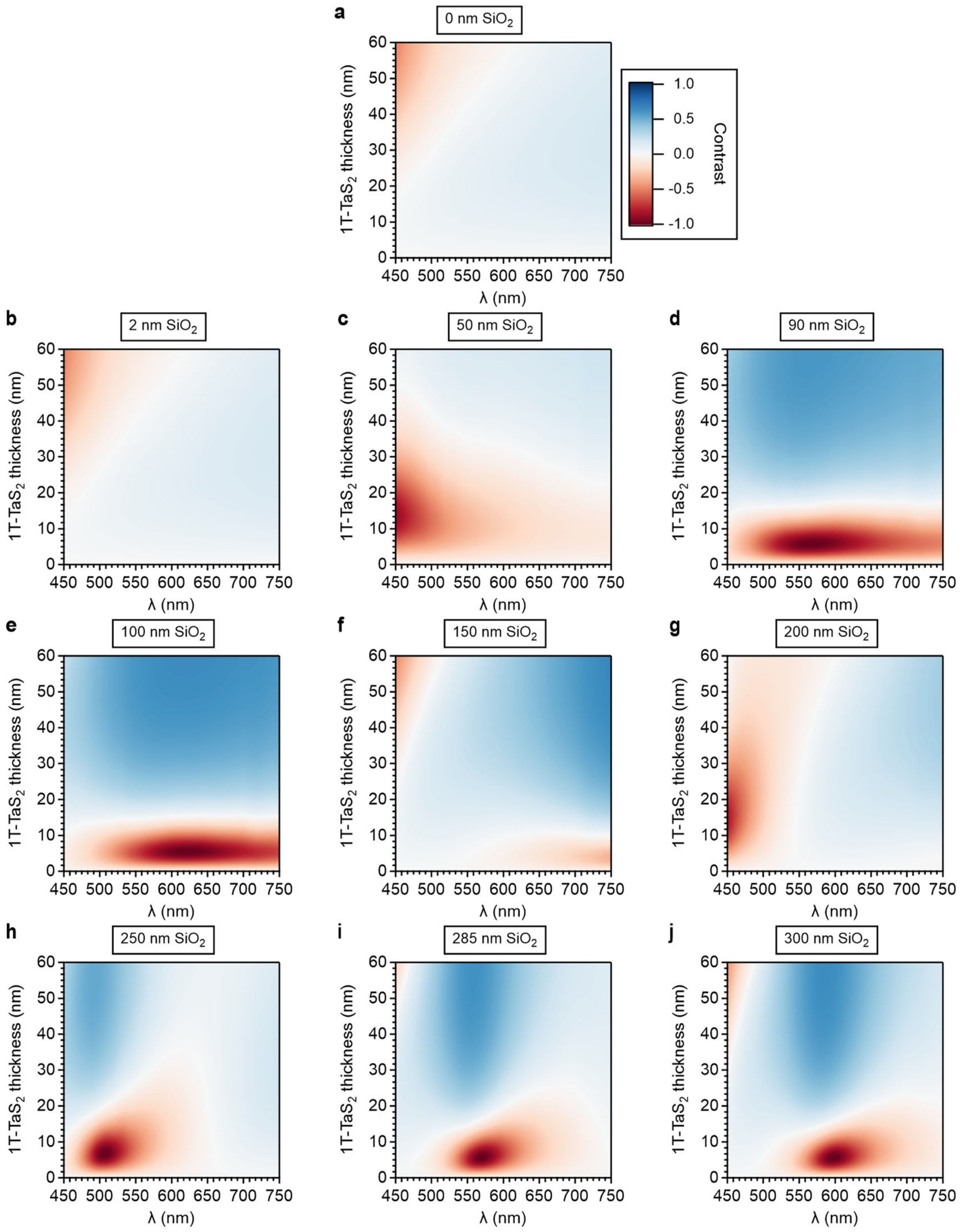

Figure S.2.- Optical contrast as a function of the incident wavelength and the 1T-TaS_2 thickness for different SiO_2 thicknesses for NA = 0 (normal incidence).

1.4. Optical contrast of 1T-TaS_2 thin-layers layer (0 – 60 nm) on 285 nm SiO_2/Si substrates: numerical aperture and wavelength dependence.

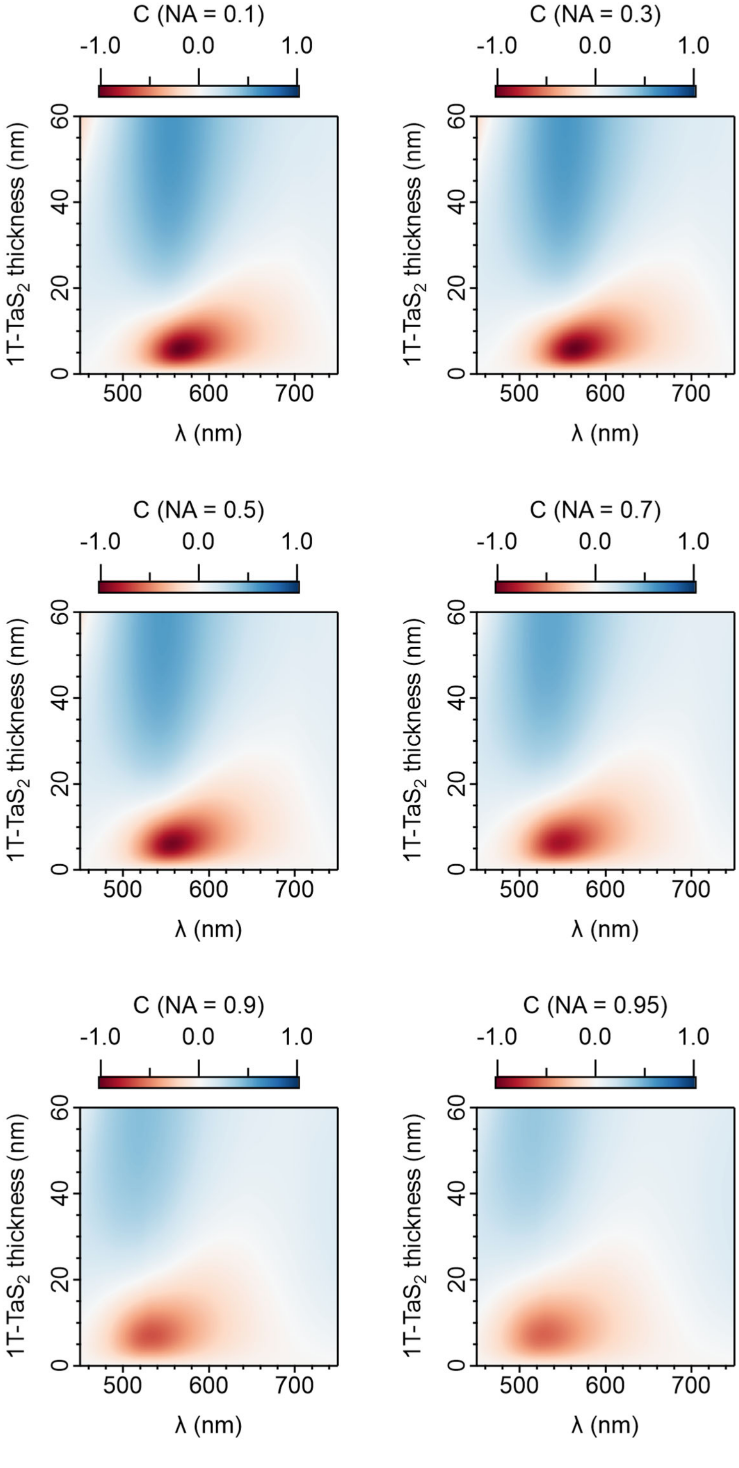

Figure S.3.- Optical contrast as a function of the incident wavelength and the 1T-TaS_2 thickness for different NAs and 285 nm of SiO_2.

1.5. Examples of experimental optical contrast of 1T-TaS_2 thin-layers on 285 nm SiO_2/Si substrates with an objective lens of NA = 0.9.

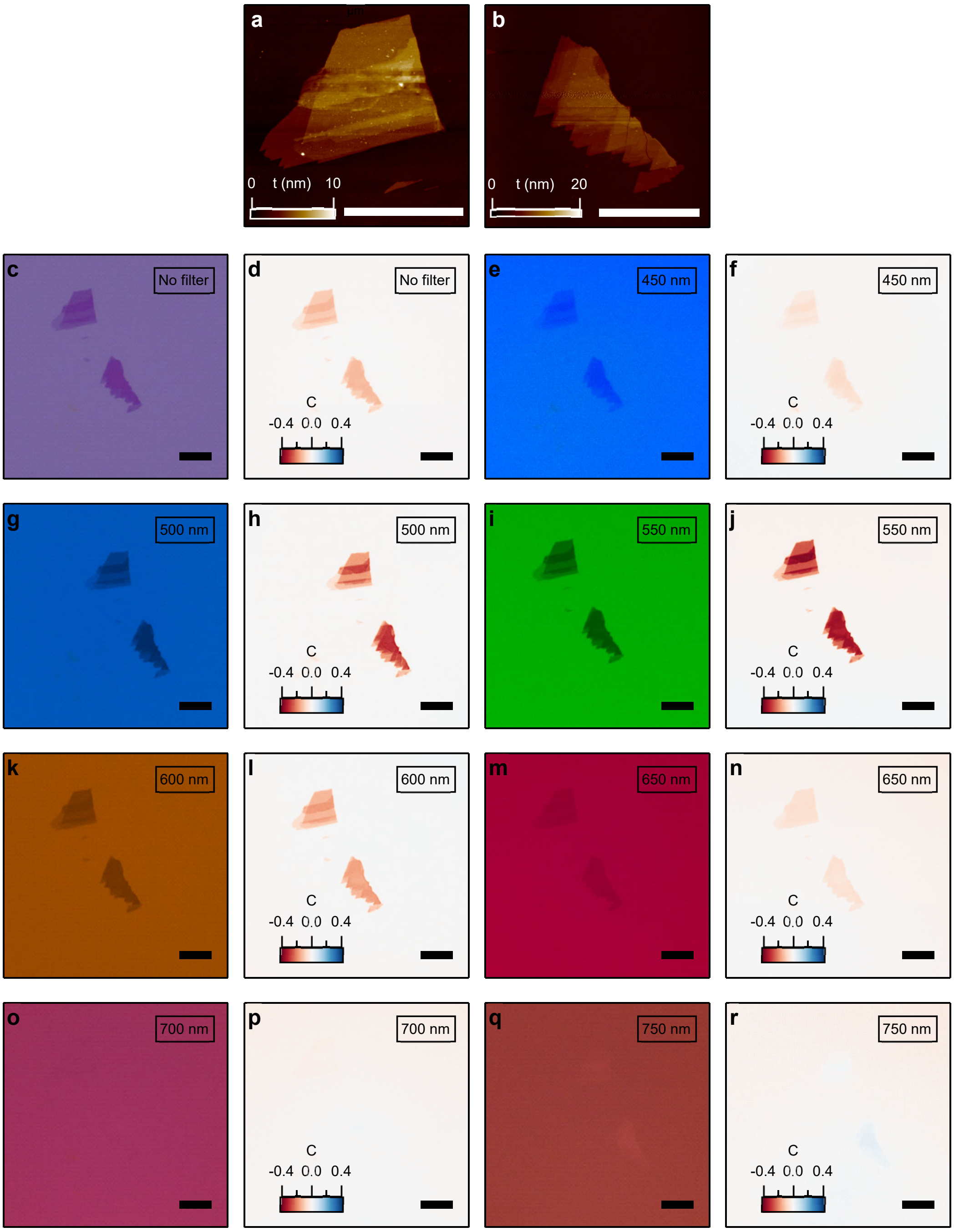

Figure S.4.- Experimental optical contrast of 1T-TaS_2 thin-layers: atomic force microscopy image of the flakes (panels a and b), optical microscopy images without filter (panel C) and with filters (panels e, g, i, k, m, o and q and s) and the corresponding experimental optical contrast (panels d, f, h, j, l, n, p and r). Scale bar: 10 µm.

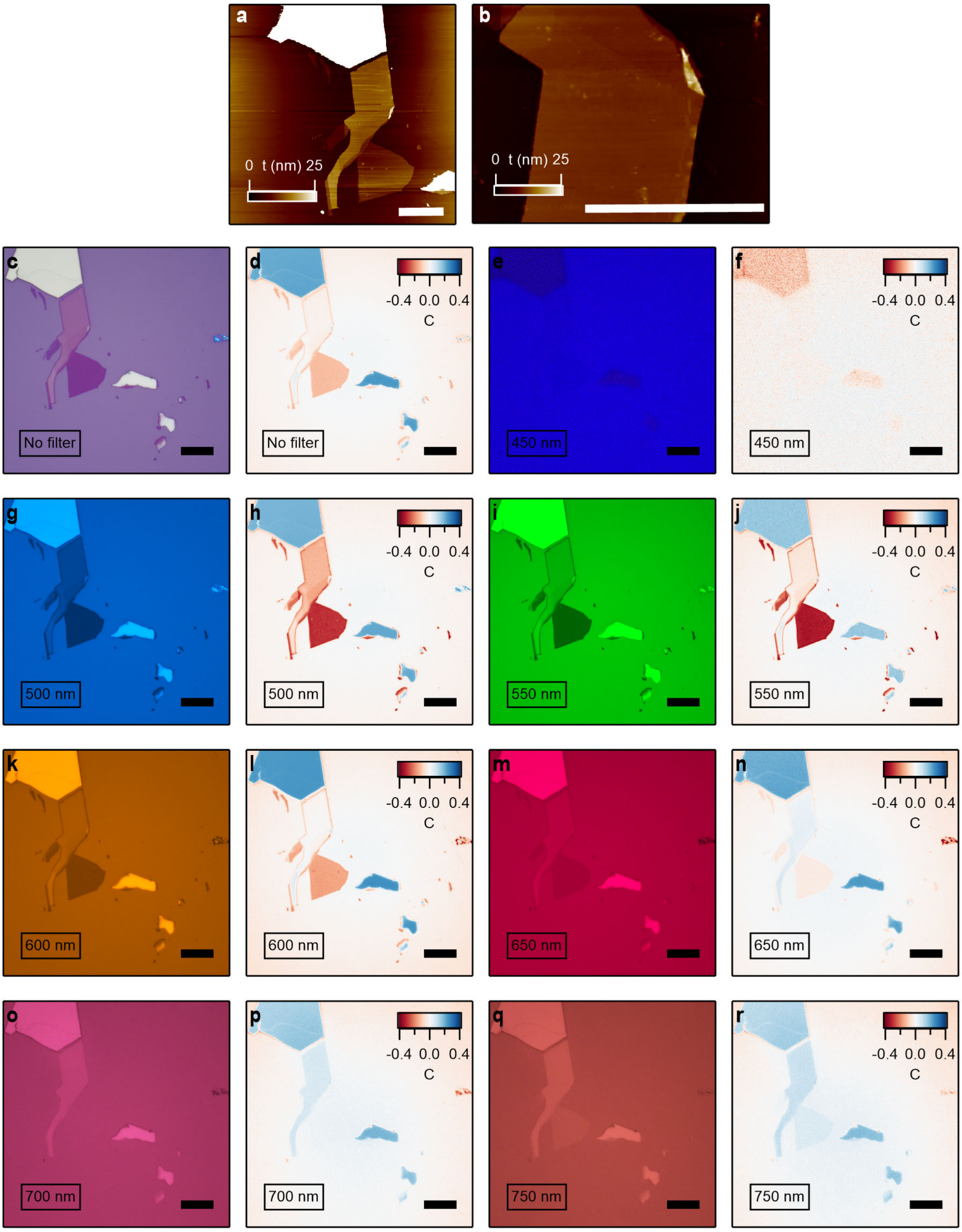

Figure S.5.- Experimental optical contrast of 1T-TaS_2 thicker-layers: atomic force microscopy image of the flakes (panels a and b), optical microscopy images without filter (panel C) and with filters (panels e, g, i, k, m, o and q and s) and the corresponding experimental optical contrast (panels d, f, h, j, l, n, p and r). Scale bar: 10 µm

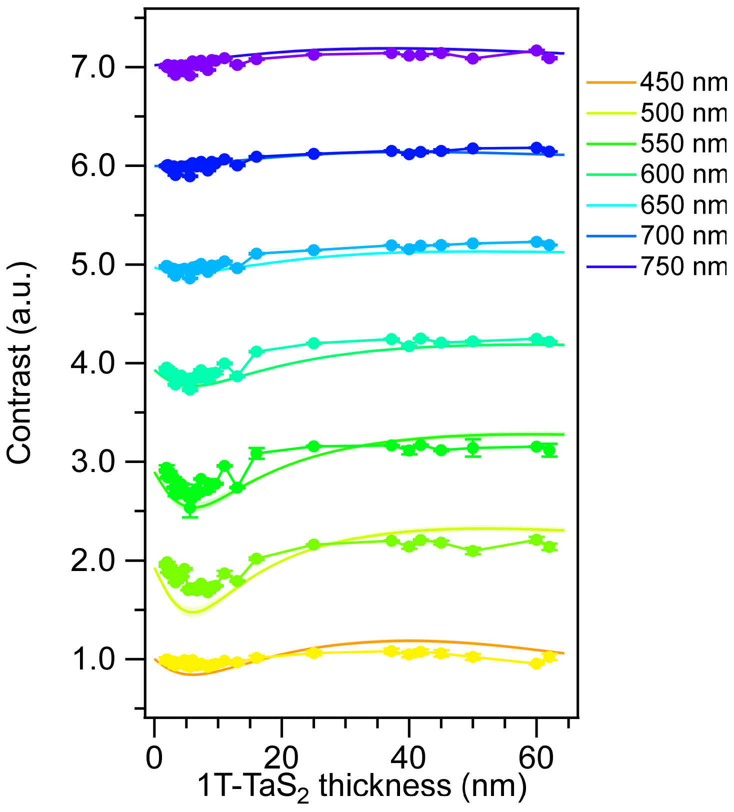

Figure S.6.- Theoretical contrast (solid lines) for thin-layers of 1T-TaS_2 on 285 nm SiO_2/Si substrates under illumination with different wavelengths and considering a NA of 0.9 together with experimental points (marks) obtained with an objective lens of NA = 0.9 and the corresponding light filters. For clarity, it has been added a vertical offset of 1.

2. Comparative of in-plane *vs.* out-of-plane devices.

Reported transport data of 1T-TaS_2 thin-layers (from Yu *et al.*[14] and Yoshida *et al.*[15]) measured in a typical in-plane configuration are compared with the out-of-plane measurements of this work in **Figures S.7** and **Figures S.8**, where previously reported transitions observed in bulk 1T-TaS_2 have been highlighted (see main manuscript). In addition, it is shown as well bulk data measured in-plane and out-of-plane (from Martino *et al.*[16]).

In order to compare all the measurements (Yu *et al.* present resistance values whereas Yoshida *et al.* show resistivity in Ω·cm and Martino *et al.* resistivity in mΩ·cm), the values are normalized to the highest temperature (HT) value, at *ca.* 400 K, thus R_{norm} = R(T)/R(HT). As general trend, the temperature variation of R_{norm} is several orders of magnitude lower in our out-of-plane measurements than in the reported in-plane ones. As well, the thermal variation of R_{norm} presents a sharper change at low-temperatures in the out-of-plane configuration whereas in the in-plane configuration R_{norm} increases for temperatures below 350 K. The change in resistance observed in the hysteresis at 350 K is larger in the in-plane configuration that in the out-of-plane one for bulk 1T-TaS_2.

In **Figure S.7** it is shown the temperature variation of R_{norm} in the full temperature range whereas in Figure S.8 it is presented the high-temperature regime, being observable in the thin-layers the N-CDW to I-CDW transition (350 K) but not the C-CDW to N-CDW one (200 K).

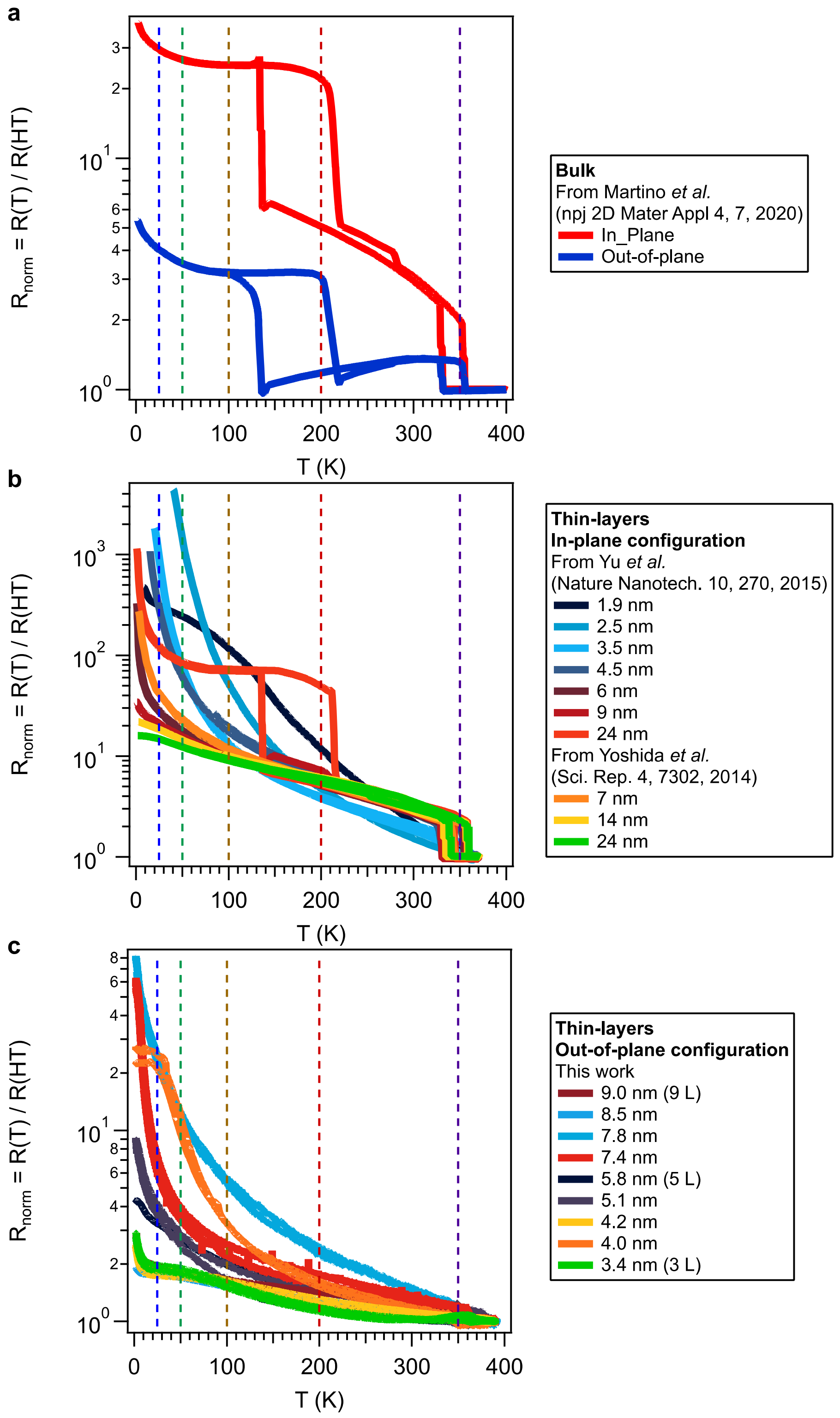

Figure S.7.- Comparative of in-plane and out-of-plane transport measurements on 1T-TaS_2 bulk and thin-layers. a) Reported values for bulk 1T-TaS_2 measured in-plane and out-of-plane. b) In-plane reported values for 1T-TaS_2 thin-layers. c) Out-of-plane transport measurements (this work) based on 1T-TaS_2 thin-layers and few-layer graphene van der Waals heterostructures (the 1T-TaS_2 thickness in nm was obtained by AFM whereas the number of layers in brackets was obtained by STEM images). Reported transitions temperatures on bulk 1T-TaS_2 have been highlighted with dashed lines (see main text).

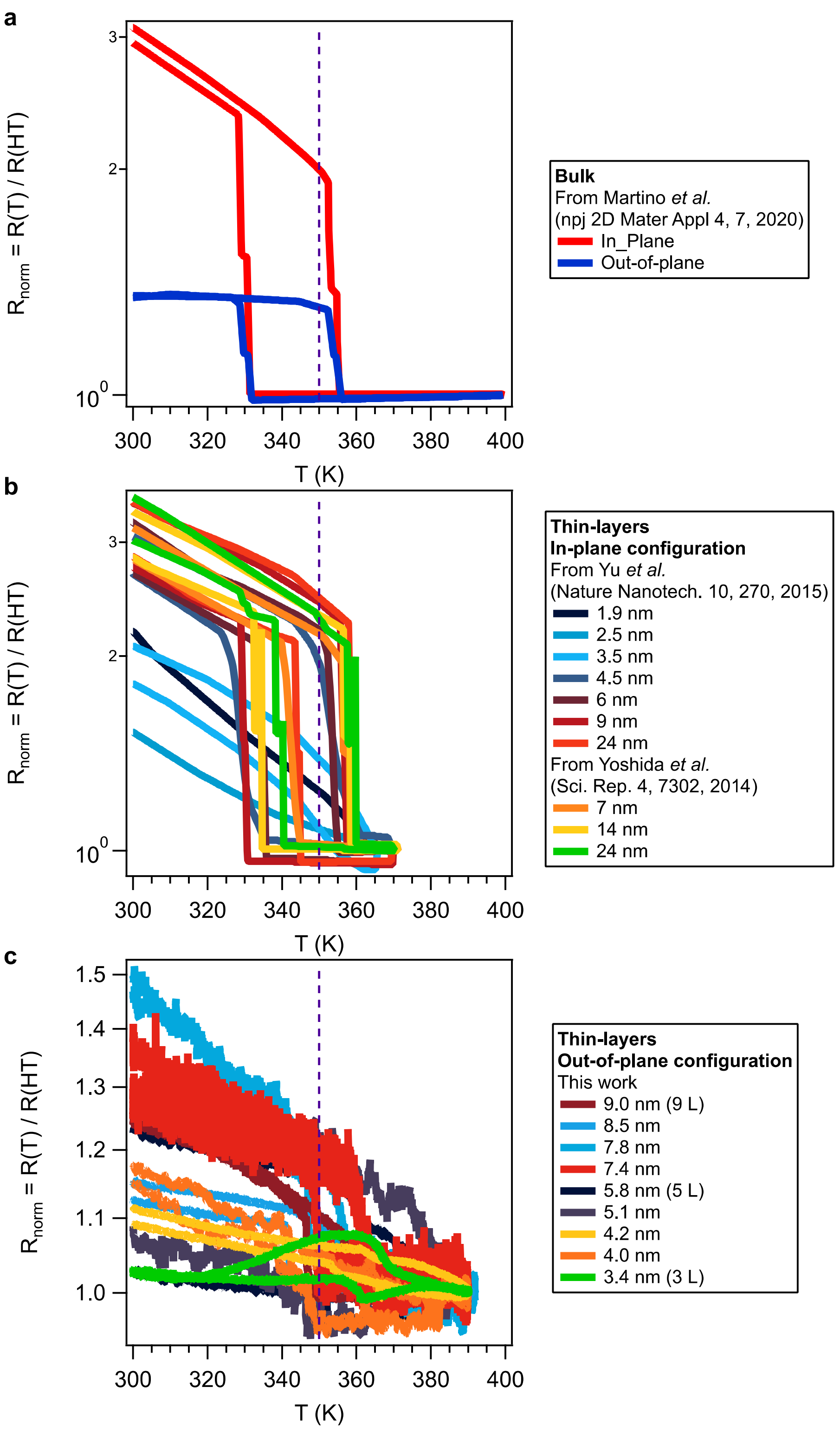

Figure S.8.- Comparative of in-plane and out-of-plane transport measurements on 1T-TaS_2 bulk and thin-layers in the high-temperature regime. a) Reported values for bulk 1T-TaS_2 measured in-plane and out-of-plane. b) In-plane reported values for 1T-TaS_2 thin-layers. c) Out-of-plane transport measurements (this work) based on 1T-TaS_2 thin-layers and few-layer graphene van der Waals heterostructures (the 1T-TaS_2 thickness in nm was obtained by AFM whereas the number of layers in brackets was obtained by STEM images). Reported transitions temperatures on bulk 1T-TaS_2 have been highlighted with dashed lines (see main text).

3. Junction geometrical factors, Arrhenius and hopping analysis.

The area of the junction and the thickness of the 1T-TaS_2 flake as well as the bottom and top few-layer graphene contacts measured by atomic force microscopy (AFM) for all the devices are summarized in **Table S.1**. As well, it is indicated the number of layers of 1T-TaS_2 were STEM cross sectional images have been performed together with the theoretical thickness in nm (considering the crystal structure of 1T-TaS_2,[17] the thickness of 1 layer of 1T-TaS_2 is 0.59 nm). It can be observed that the measured thickness by AFM is higher than the one observed by STEM. This is ascribed to the presence of adsorbates and residues from the exfoliation and transfer process that yield to a thickness higher than the real one in AFM measurements.[18]

Resistivity curves with the geometrical factors in **Table S.1** are shown in **Figure S.9**.

The different devices were fitted following an Arrhenius expression for the conductance:

$$G = G_0 e^{-\frac{E_a}{k_B T}}$$

where G_0 is a prefactor, E_a is the activation energy, k_B is the Boltzmann constant and T is the temperature.[19]

The fitting for the different devices (from **Figure S.10** to **Figure S.27**) were performed in the low temperature regime (below 10 K) and in the range of 200 K- 350 K (range of the N-CDW). The temperature of the intersection between the slopes obtained in the two previous limiting cases was defined as T_{cross}. For simplicity, key temperatures as 25 K, 50 K, 100 K, 200 K and 350 K have been marked. The obtained values are summarized in **Table S.2** (warming from 2 K to 400 K), **Table S.3** (cooling from 400 K to 2 K) and the mean values in **Table S.4**.

In addition, other transport mechanism as variable range or nearest-neighbor hopping have been considered. The conductance takes the form:[20]

$$G = G_0 \exp\left(-\frac{T_0}{T}\right)^x$$

being G_0 the residual conductance, T_0 the characteristic hopping temperature and x the hopping exponent. G_0 can be temperature independent or of the form $G_0 = AT^{-m}$, where A is a constant and m varies from 0.8 to 1 (we did not find significant changes by varying m from 0.8 to 1 and we show the results for m = 1).[20,21] The exponent x determines the scaling behavior. We consider the cases of x = 1/3 (2D-VRH), x = 1/4 (3D-VRH) and x = 1 (nearest-neighbor-hopping, NNH). We note that, formally, the analysis with x = 1 takes the same expression as the Arrhenius analysis. The different results are shown in **Figure S.28** – **Figure S.45**. Any of the models give rise to a single conductance mechanism for all the temperature range. However, due to the multiple transitions reported in $1T\text{-}TaS_2$, it is not possible at the moment to unveil the real transport mechanism.

3.1.- Junction geometrical factors.

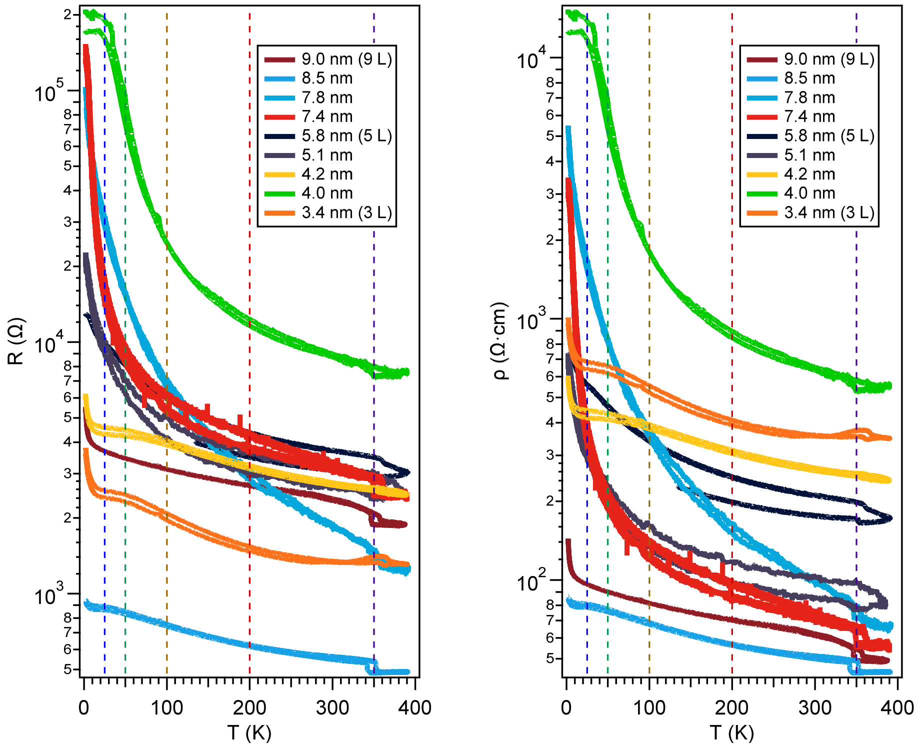

Figure S.9.- Resistance (left) and resistivity (right) for the different devices (the $1T\text{-}TaS_2$ thickness in nm was obtained by AFM whereas the number of layers in brackets was obtained by STEM images), considering the area and thickness of $1T\text{-}TaS_2$ by atomic force microscopy reported in Table S.1. The dashed lines highlight the different transitions described in the literature for bulk $1T\text{-}TaS_2$ (see main text).

Table S.1.- Estimated area of the junction (from optical microscopy pictures) and thickness for the bottom and top FLG contacts and $1T-TaS_2$ by atomic force microscopy. As well, it is indicated the number of layers of $1T-TaS_2$ were STEM cross sectional images have been performed with the corresponding thickness in nm (thickness of $1T-TaS_2$ monolayer is 0.59 nm).

Device	Area (μm^2)	Bottom contact thickness (nm)	Top contact thickness (nm)	$1T-TaS_2$ thickness by AFM (nm)	$1T-TaS_2$ layers by STEM	$1T-TaS_2$ thickness by STEM (nm)
A	2.35	1.2	1.0	9.0	9	5.3
B	9.05	2.0	1.5	3.4	3	1.8
C	1.68	1.0	4.2	7.4	-	-
D	3.30	3.5	2.0	5.8	5	3.0
E	1.65	5.2	4.2	5.1	-	-
F	7.74	4.6	4.5	8.5	-	-
G	4.15	3.5	4.4	7.8	-	-
H	4.08	3.3	1.3	4.2	-	-
I	2.91	3.7	1.4	4.0	-	-

3.2.- Arrhenius analysis.

Table S.2.- Summary of the values obtained for the different devices while sweeping the temperature from 2 K to 400 K at 1K/min. The conductance was modelled by an Arrhenius law in the low (LT; below 10 K) and high (HT; in the N-CDW range of 200 K - 350 K) temperature regimes. E_a is the activation energy, G_0 a prefactor and T_{cross} is the temperature where the fittings for the LT and HT regimes intersect. Errors are indicated in brackets.

Device [Warming: 2 K → 400 K]	E_a (meV)		G_0 (mS)		T_{cross} (K)
	LT	HT	LT	HT	
A	0.09198(8)	4.269(7)	0.3994(1.2)	0.8060(3)	69
B	0.0600(4)	6.506(6)	0.38223(6)	0.9903(3)	79
C	0.065(2)	10.840(2)	0.01035(8)	0.4881(4)	32
D	0.00196(3)	8.485(6)	0.07873(7)	0.37498(9)	63
E	0.0235(5)	7.54(4)	0.0514(2)	0.4275(8)	42
F	0.01532(3)	6.330(5)	1.0401(2)	2.2207(6)	95
G	0.0986(9)	23.56(6)	0.01796(6)	1.349(4)	63
H	0.0499(2)	8.389(5)	0.2158(2)	0.5007(2)	114.9
I	0.0006(3)	15.85(2)	0.005827(3)	0.2140(2)	51.1

Table S.3.- Summary of the values obtained for the different devices while sweeping the temperature from 400 K to 2 K at 1 K/min. The conductance was modelled by an Arrhenius law in the low (LT; below 10 K) and high (HT; in the N-CDW range of 200 K - 350 K) temperature regimes. E_a is the activation energy, G_0 a prefactor and T_{cross} is the temperature where the fittings for the LT and HT regimes intersect. Errors are indicated in brackets.

Device [Cooling: 400 K → 2 K]	E_a (meV)		G_0 (mS)		T_{cross} (K)
	LT	HT	LT	HT	
A	0.07647(9)	5.446(1.2)	0.29457(8)	0.5532(3)	99
B	0.051(3)	7.020(4)	0.3539(4)	0.9789(2)	79
C	0.052(2)	14.65(3)	0.00880(5)	0.5374(7)	41
D	-	6.124(4)	-	0.40878(8)	-
E	0.0160(9)	7.26(6)	0.05445(1.9)	0.501(2)	38
F	0.015607(5)	6.250(4)	1.1711(2)	2.3164(4)	106
G	0.0858(9)	27.29(5)	0.01554(6)	1.542(3)	69
H	0.0544(2)	8.032(3)	0.22239(2)	0.50464(8)	113.9
I	0.0119(3)	17.079(1.2)	0.005183(6)	0.2161(2)	52.8

Table S.4.- Mean values and standard deviations of the Arrhenius plot parameters for the different devices reported in Table S.2 and Table S.3.

Mean Values	E_a (meV)		G_0 (mS)		T_{cross} (K)
	LT	HT	LT	HT	
Warming: 2 K → 400 K]	0.05 ± 0.04	10 ± 6	0.2 ± 0.3	0.8 ± 0.6	70 ± 30
Cooling: 400 K → 2 K]	0.05 ± 0.03	11 ± 7	0.3 ± 0.4	0.8 ± 0.7	70 ± 30
Warming and cooling	0.05 ± 0.03	11 ± 7	0.3 ± 0.4	0.8 ± 0.6	70 ± 30

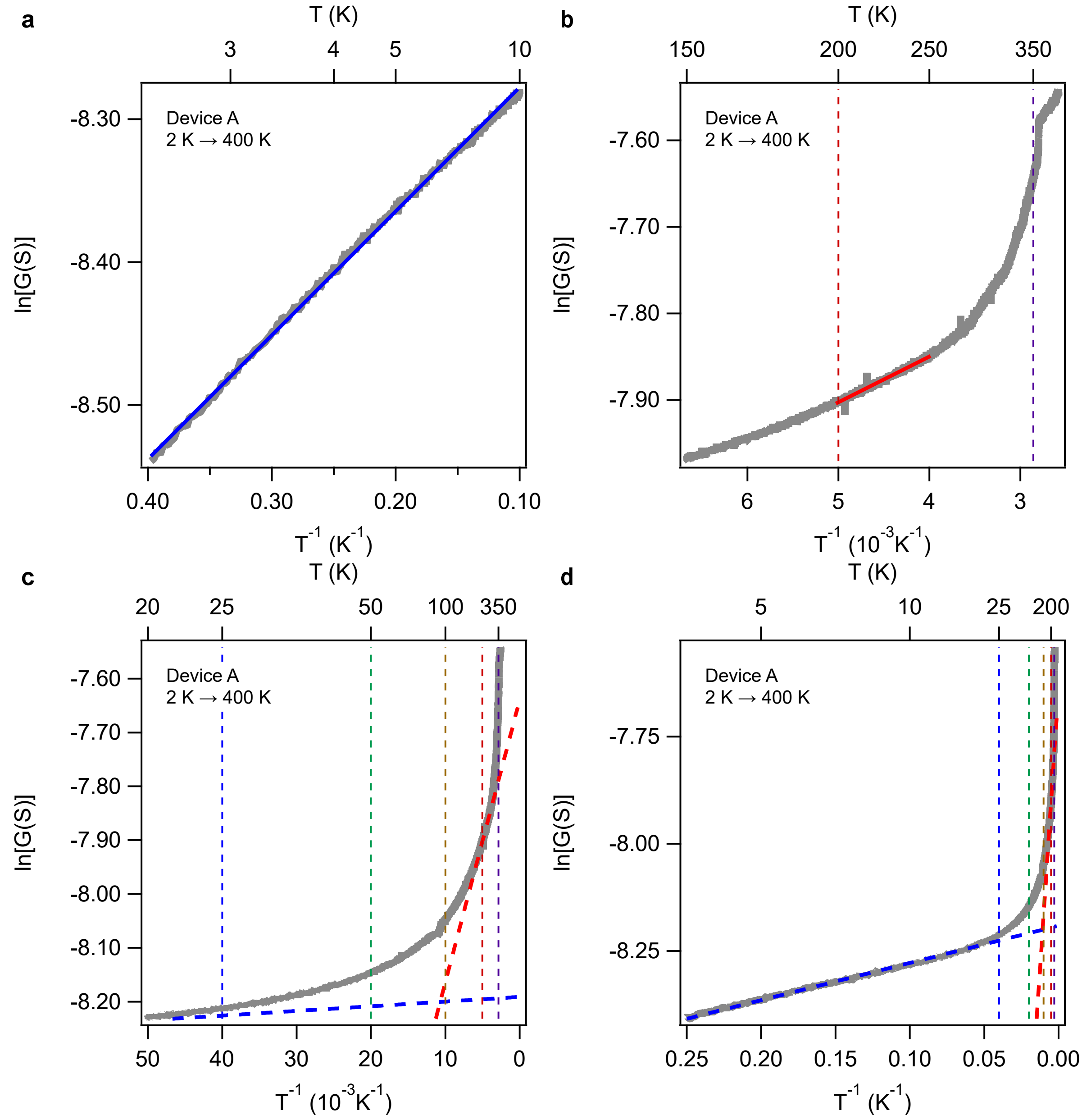

Figure S.10.- Arrhenius plot of the conductance (Device A) while sweeping the temperature from 2 K to 400 K at 1 K/min. a) Low-temperature regime with the fit shown in blue. b) High-temperature regime with the fit shown in red. c) Intermediate region and d) a broader temperature range where the previous fit values are prolonged in blue and red dashed lines. The intersection of both lines define T_{cross}. The temperatures of 25 K (blue), 50 K (green), 100 K (brown), 200 K (red) and 350 K (purple) have been marked as vertical dashed lines, where previously reports have observed transitions in bulk 1T-TaS_2 (see main text).

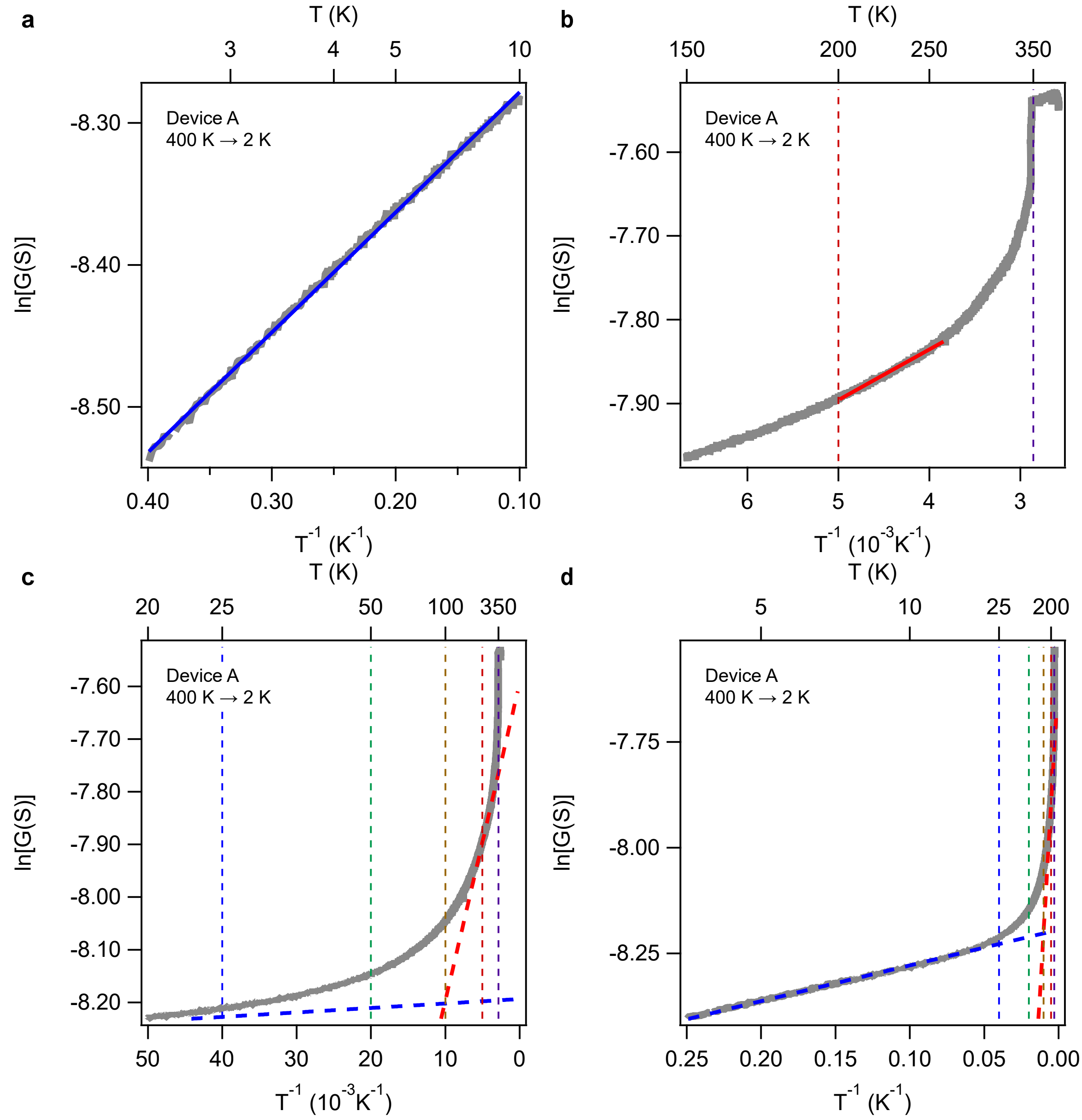

Figure S.11.- Arrhenius plot of the conductance (Device A) while sweeping the temperature from 400 K to 2 K at 1 K/min. a) Low-temperature regime with the fit shown in blue. b) High-temperature regime with the fit shown in red. c) Intermediate region and d) a broader temperature range where the previous fit values are prolonged in blue and red dashed lines. The intersection of both lines define T_{cross}. The temperatures of 25 K (blue), 50 K (green), 100 K (brown), 200 K (red) and 350 K (purple) have been marked as vertical dashed lines, where previously reports have observed transitions in bulk 1T-TaS_2 (see main text).

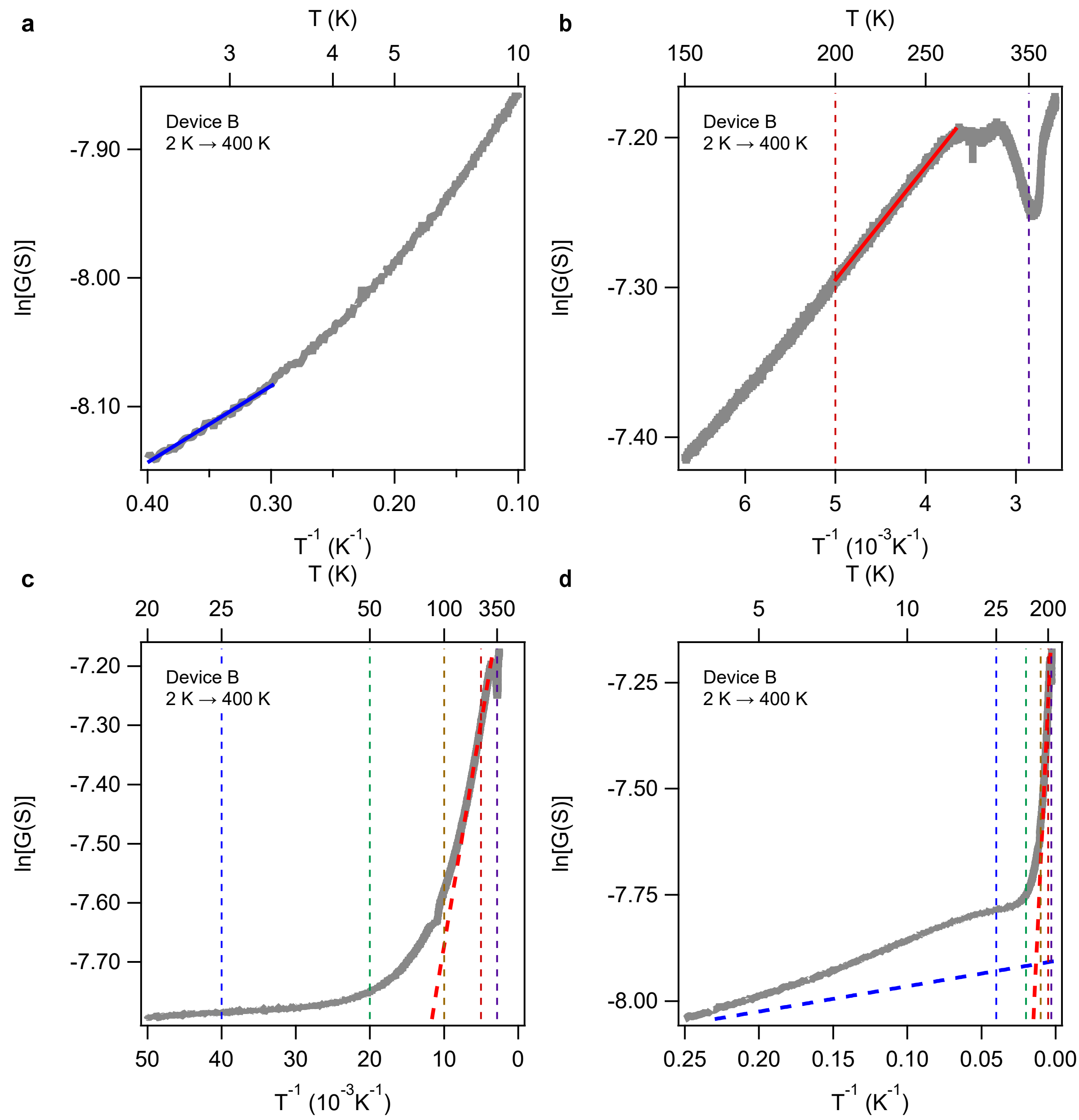

Figure S.12.- Arrhenius plot of the conductance (Device B) while sweeping the temperature from 2 K to 400 K at 1 K/min. a) Low-temperature regime with the fit shown in blue. b) High-temperature regime with the fit shown in red. c) Intermediate region and d) a broader temperature range where the previous fit values are prolonged in blue and red dashed lines. The intersection of both lines define T_{cross}. The temperatures of 25 K (blue), 50 K (green), 100 K (brown), 200 K (red) and 350 K (purple) have been marked as vertical dashed lines, where previously reports have observed transitions in bulk 1T-TaS_2 (see main text).

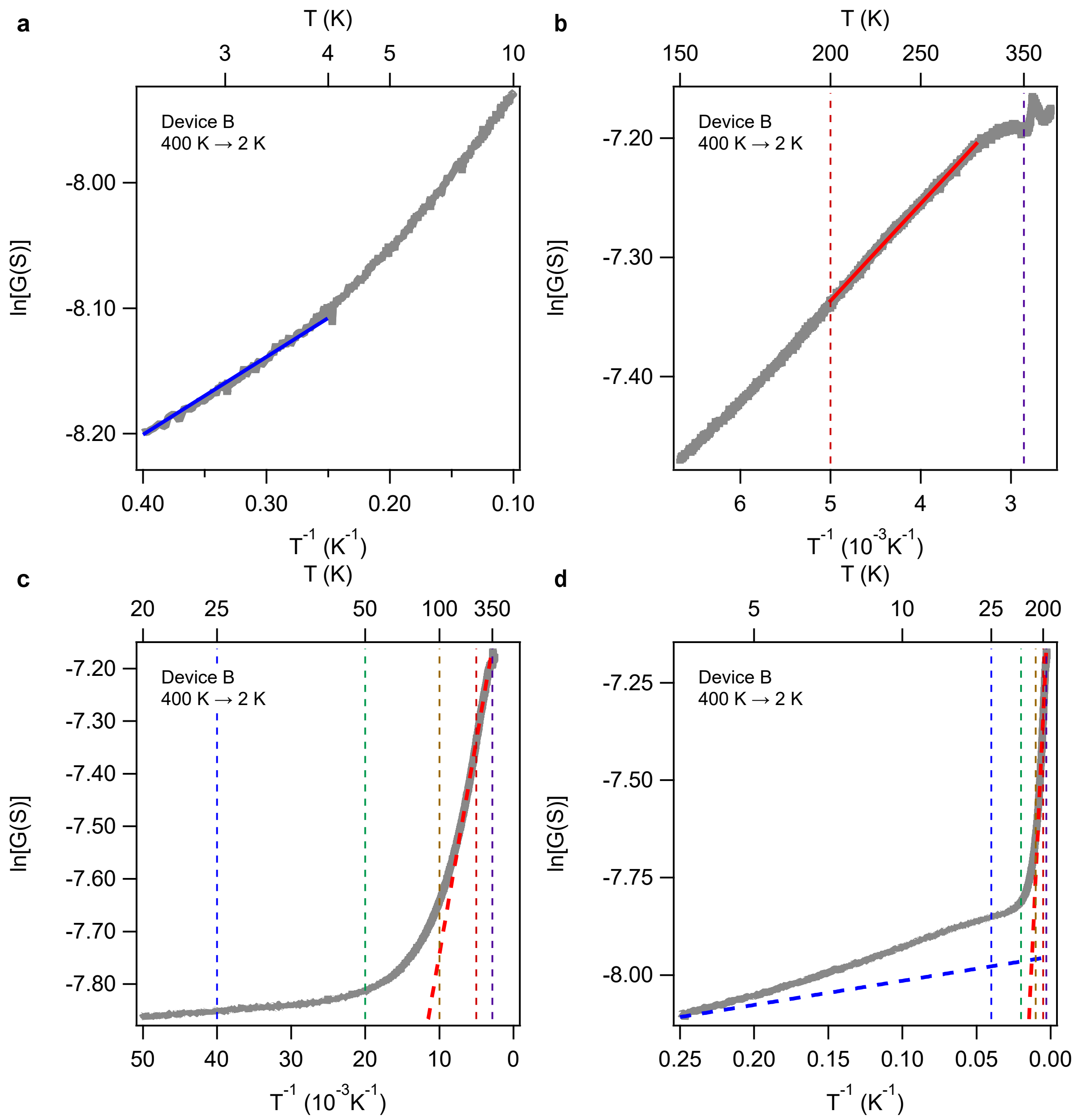

Figure S.13.- Arrhenius plot of the conductance (Device B) while sweeping the temperature from 400 K to 2 K at 1 K/min. a) Low-temperature regime with the fit shown in blue. b) High-temperature regime with the fit shown in red. c) Intermediate region and d) a broader temperature range where the previous fit values are prolonged in blue and red dashed lines. The intersection of both lines define T_{cross}. The temperatures of 25 K (blue), 50 K (green), 100 K (brown), 200 K (red) and 350 K (purple) have been marked as vertical dashed lines, where previously reports have observed transitions in bulk 1T-TaS_2 (see main text).

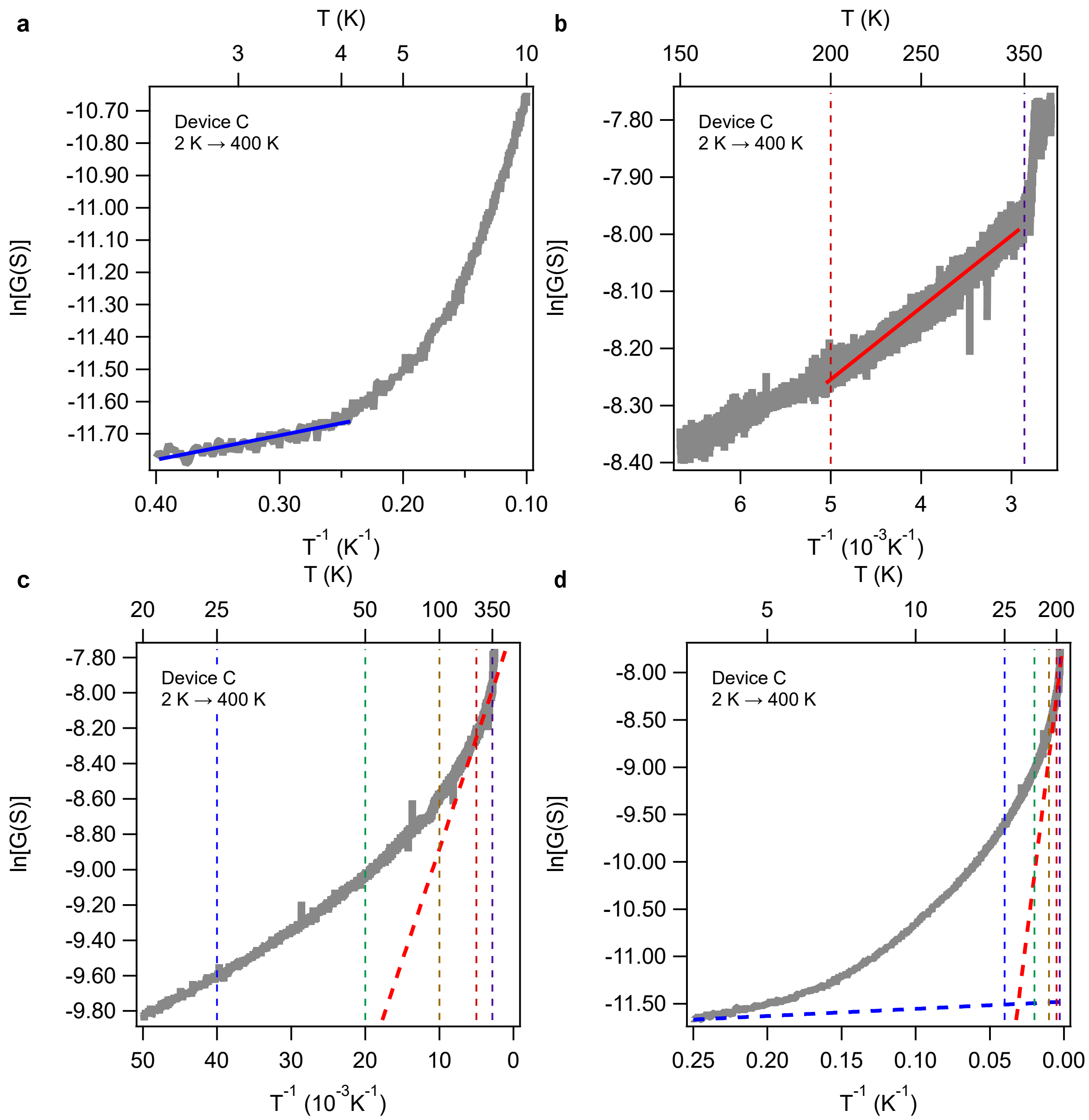

Figure S.14.- Arrhenius plot of the conductance (Device C) while sweeping the temperature from 2 K to 400 K at 1 K/min. a) Low-temperature regime with the fit shown in blue. b) High-temperature regime with the fit shown in red. c) Intermediate region and d) a broader temperature range where the previous fit values are prolonged in blue and red dashed lines. The intersection of both lines define T_{cross}. The temperatures of 25 K (blue), 50 K (green), 100 K (brown), 200 K (red) and 350 K (purple) have been marked as vertical dashed lines, where previously reports have observed transitions in bulk 1T-TaS_2 (see main text).

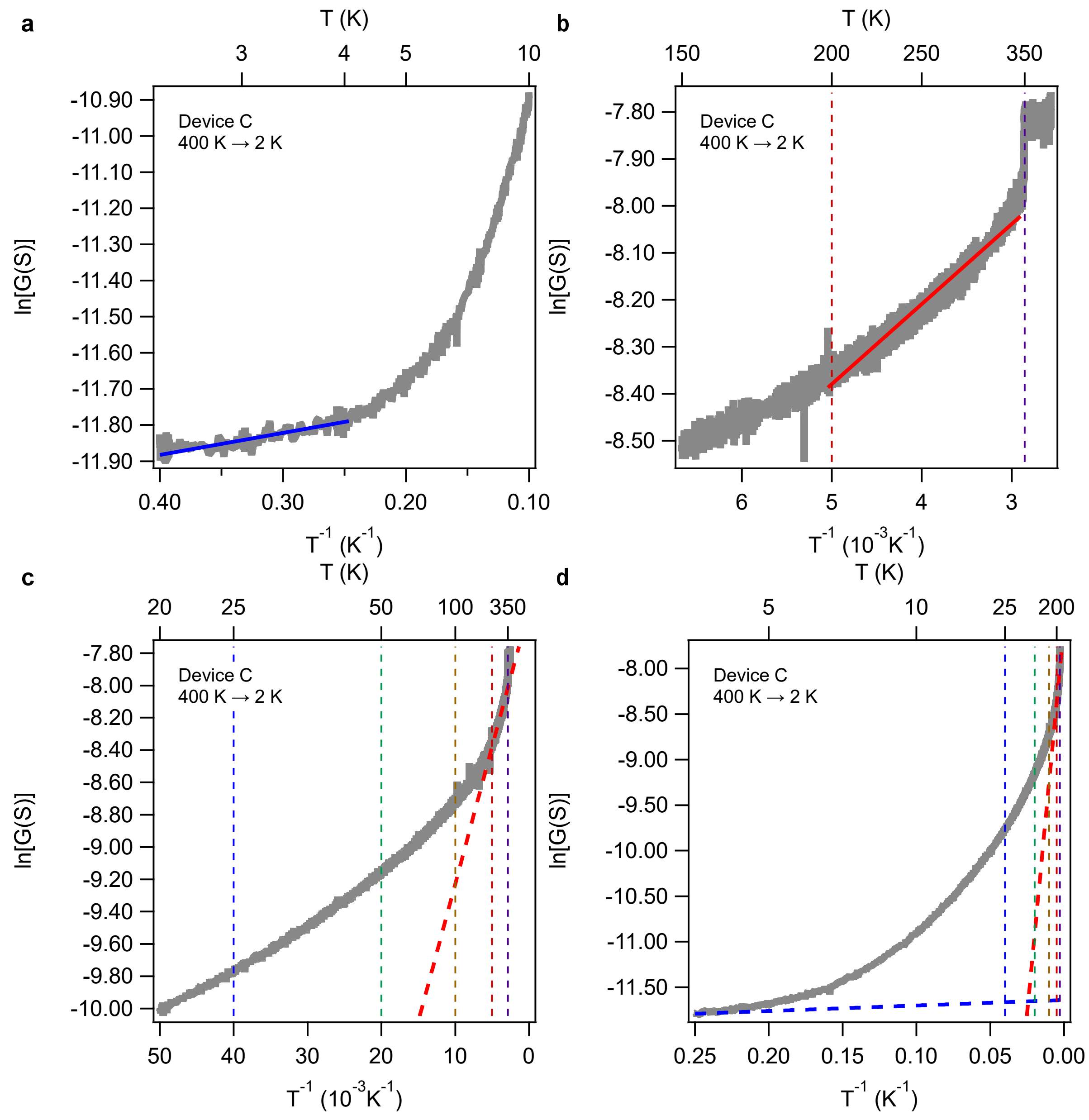

Figure S.15.- Arrhenius plot of the conductance (Device C) while sweeping the temperature from 400 K to 2 K at 1 K/min. a) Low-temperature regime with the fit shown in blue. b) High-temperature regime with the fit shown in red. c) Intermediate region and d) a broader temperature range where the previous fit values are prolonged in blue and red dashed lines. The intersection of both lines define T_{cross}. The temperatures of 25 K (blue), 50 K (green), 100 K (brown), 200 K (red) and 350 K (purple) have been marked as vertical dashed lines, where previously reports have observed transitions in bulk 1T-TaS_2 (see main text).

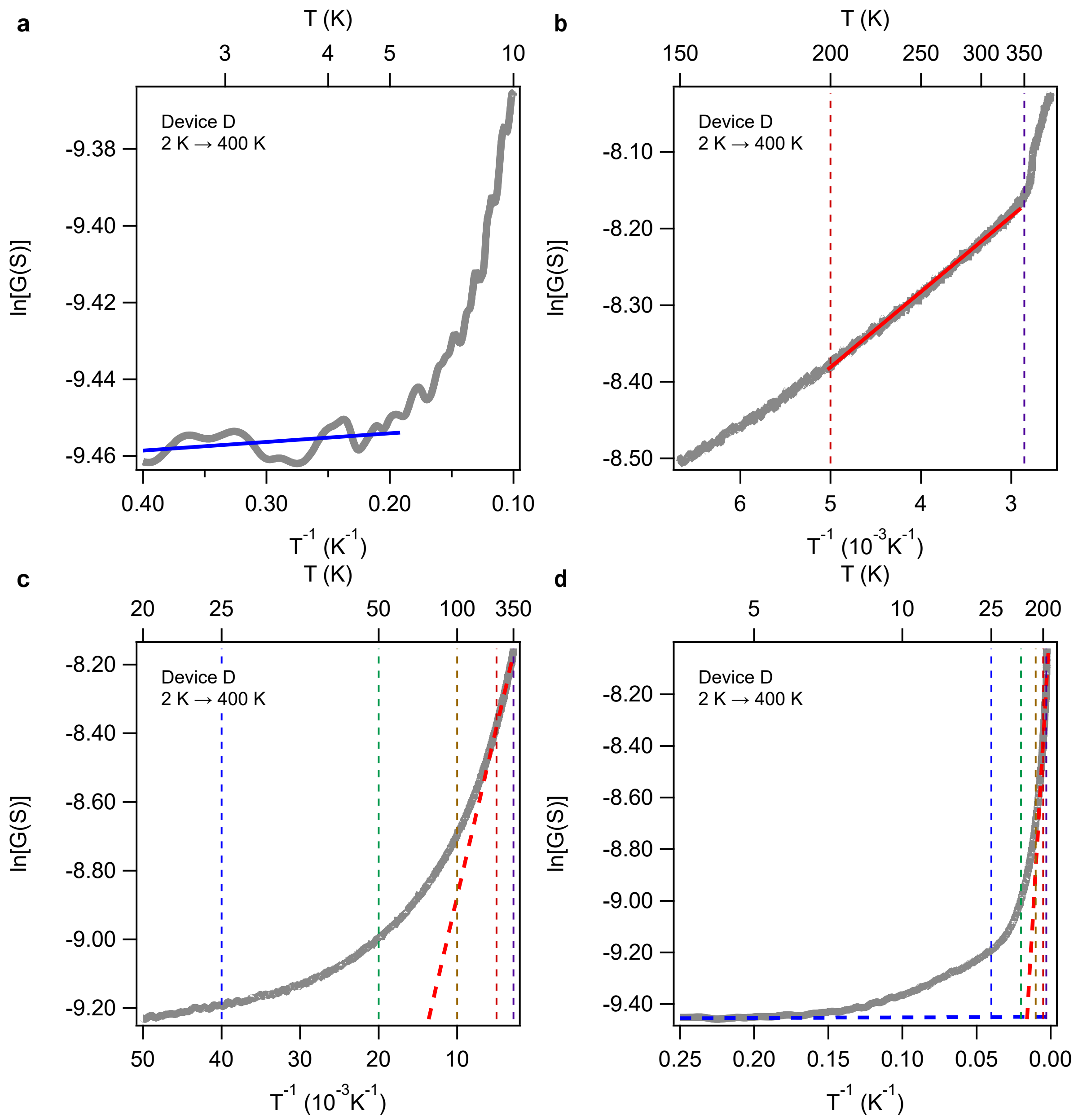

Figure S.16.- Arrhenius plot of the conductance (Device D) while sweeping the temperature from 2 K to 400 K at 1 K/min. a) Low-temperature regime with the fit shown in blue. b) High-temperature regime with the fit shown in red. c) Intermediate region and d) a broader temperature range where the previous fit values are prolonged in blue and red dashed lines. The intersection of both lines define T_{cross}. The temperatures of 25 K (blue), 50 K (green), 100 K (brown), 200 K (red) and 350 K (purple) have been marked as vertical dashed lines, where previously reports have observed transitions in bulk 1T-TaS_2 (see main text).

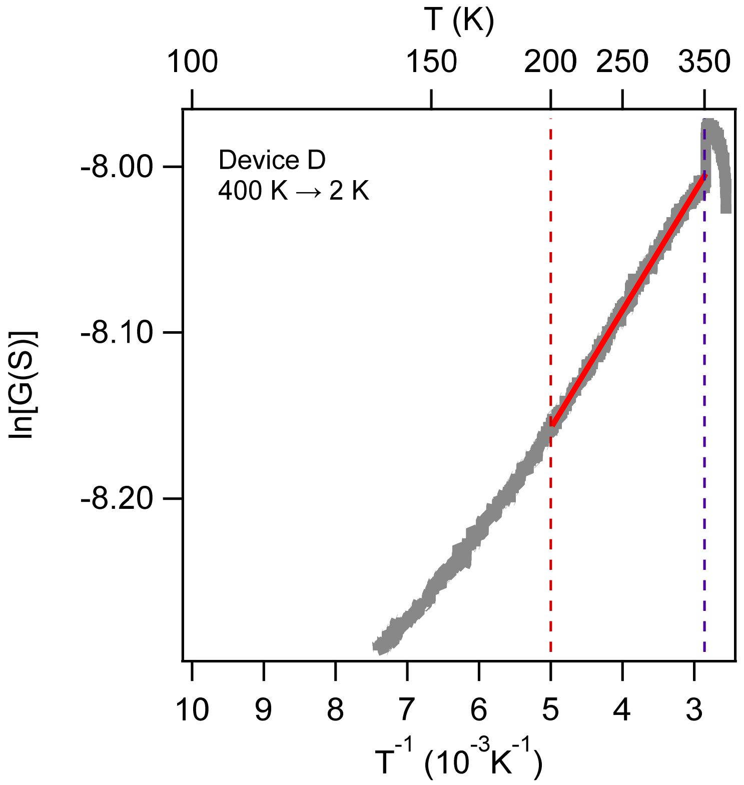

Figure S.17.- Arrhenius plot of the conductance (Device D) while sweeping the temperature from 400 K to 140 K at 1 K/min. The temperatures of 200 K (red) and 350 K (purple) have been marked as vertical dashed lines, where previously reports have observed transitions in bulk 1T-TaS_2 (see main text).

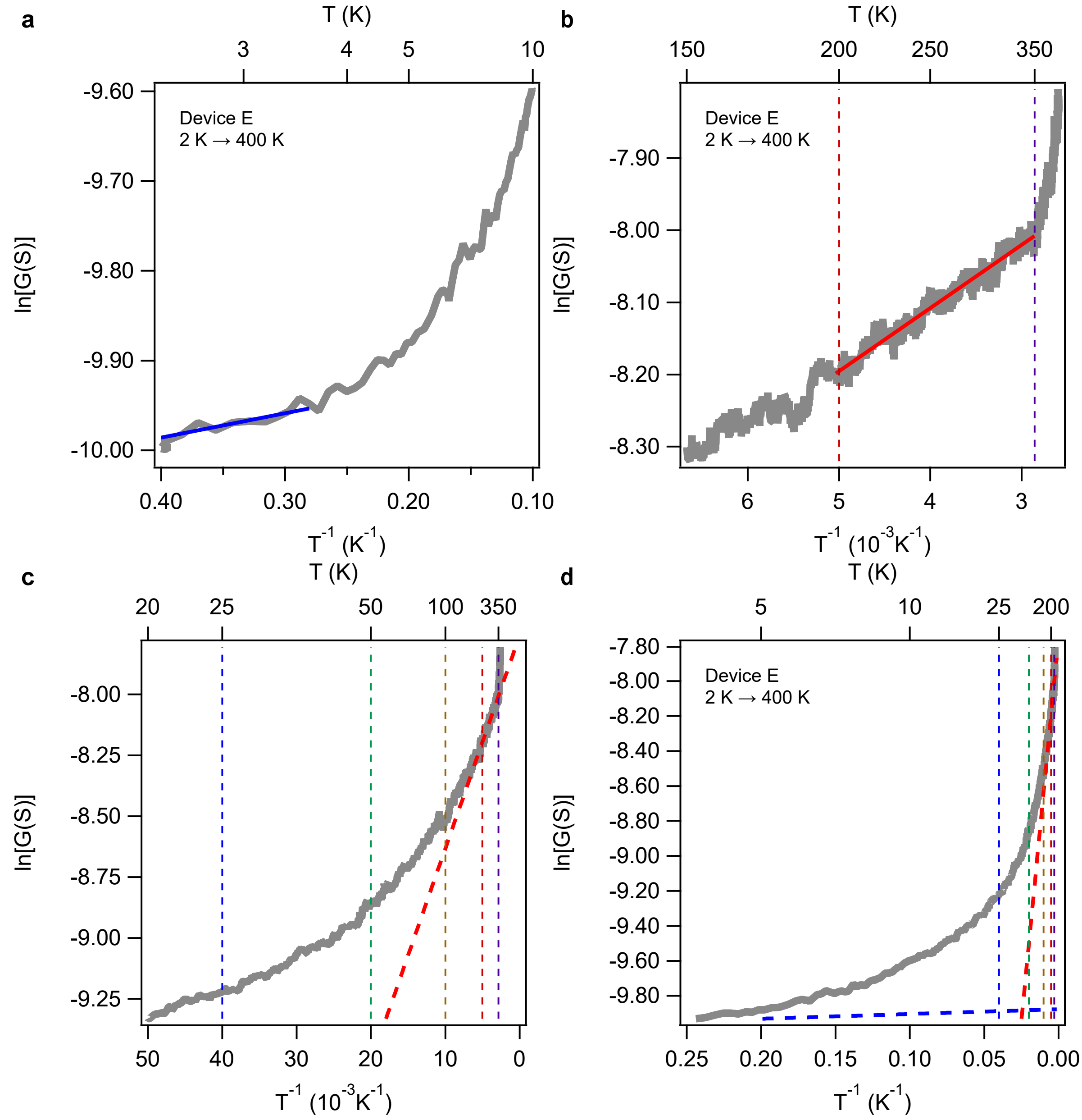

Figure S.18.- Arrhenius plot of the conductance (Device E) while sweeping the temperature from 2 K to 400 K at 1 K/min. a) Low-temperature regime with the fit shown in blue. b) High-temperature regime with the fit shown in red. c) Intermediate region and d) a broader temperature range where the previous fit values are prolonged in blue and red dashed lines. The intersection of both lines define T_{cross}. The temperatures of 25 K (blue), 50 K (green), 100 K (brown), 200 K (red) and 350 K (purple) have been marked as vertical dashed lines, where previously reports have observed transitions in bulk 1T-TaS_2 (see main text).

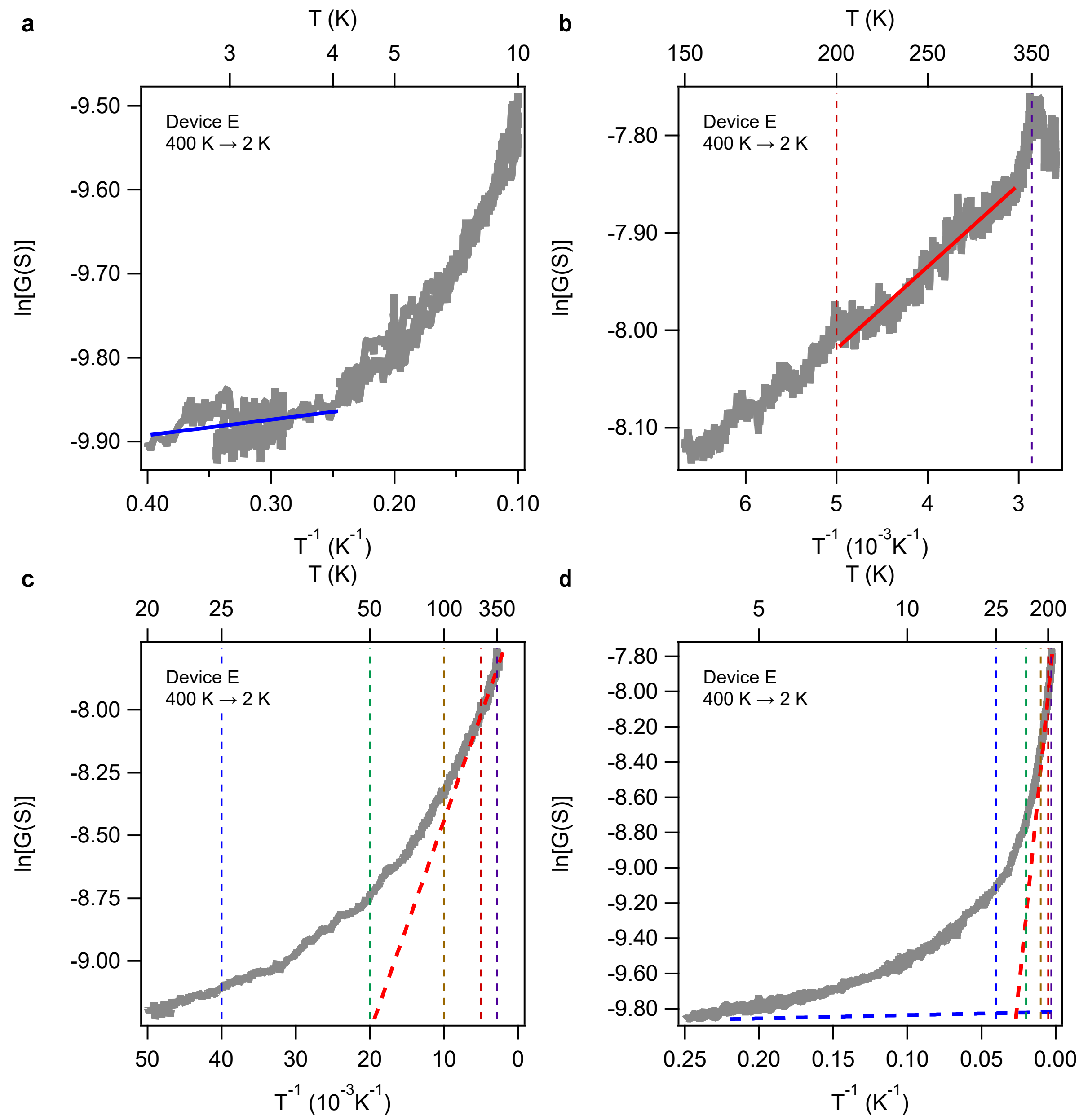

Figure S.19.- Arrhenius plot of the conductance (Device E) while sweeping the temperature from 400 K to 2 K at 1 K/min. a) Low-temperature regime with the fit shown in blue. b) High-temperature regime with the fit shown in red. c) Intermediate region and d) a broader temperature range where the previous fit values are prolonged in blue and red dashed lines. The intersection of both lines define T_{cross}. The temperatures of 25 K (blue), 50 K (green), 100 K (brown), 200 K (red) and 350 K (purple) have been marked as vertical dashed lines, where previously reports have observed transitions in bulk 1T-TaS_2 (see main text).

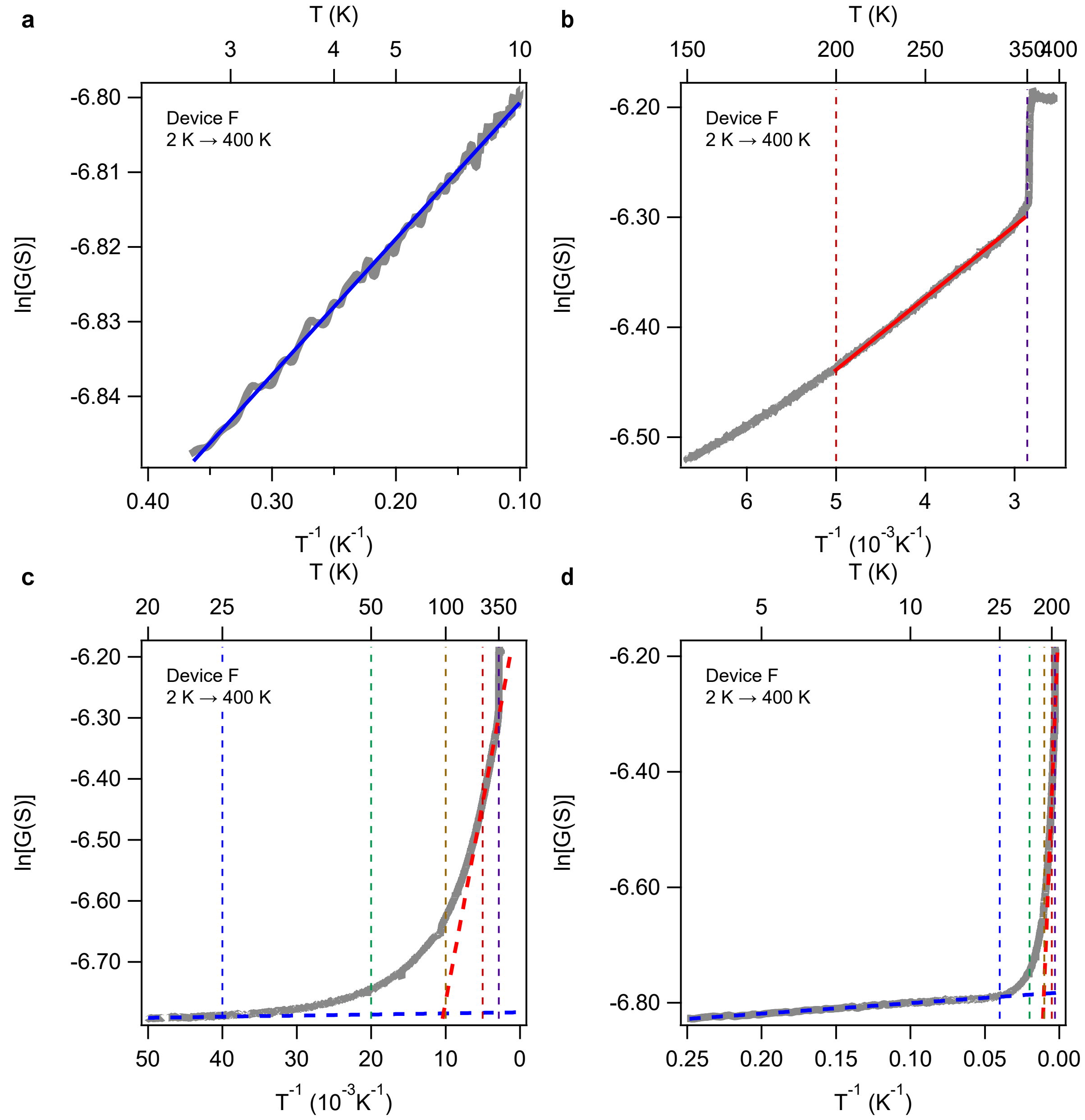

Figure S.20.- Arrhenius plot of the conductance (Device F) while sweeping the temperature from 2 K to 400 K at 1 K/min. a) Low-temperature regime with the fit shown in blue. b) High-temperature regime with the fit shown in red. c) Intermediate region and d) a broader temperature range where the previous fit values are prolonged in blue and red dashed lines. The intersection of both lines define T_{cross}. The temperatures of 25 K (blue), 50 K (green), 100 K (brown), 200 K (red) and 350 K (purple) have been marked as vertical dashed lines, where previously reports have observed transitions in bulk 1T-TaS_2 (see main text).

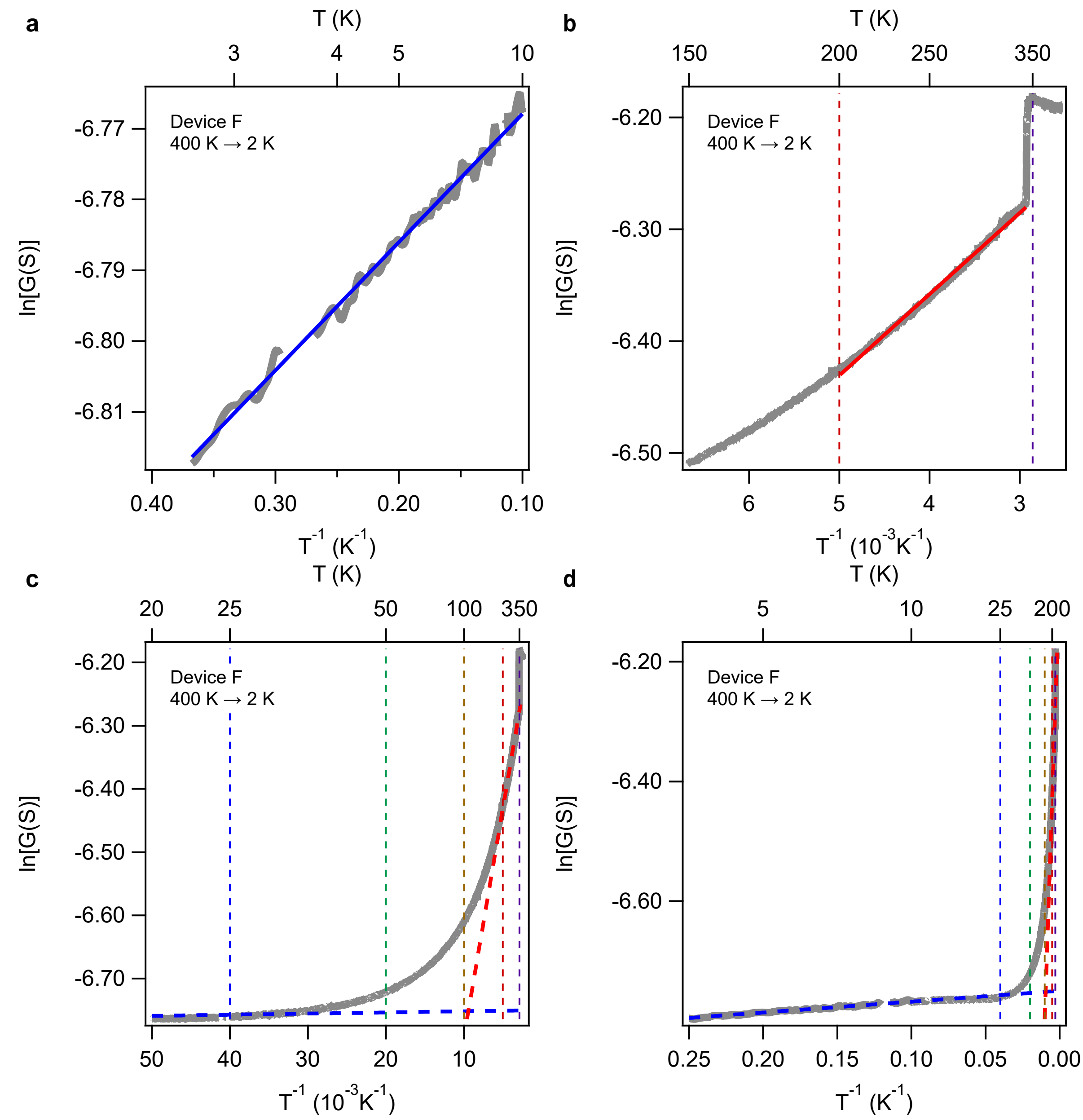

Figure S.21.- Arrhenius plot of the conductance (Device F) while sweeping the temperature from 400 K to 2 K at 1 K/min. a) Low-temperature regime with the fit shown in blue. b) High-temperature regime with the fit shown in red. c) Intermediate region and d) a broader temperature range where the previous fit values are prolonged in blue and red dashed lines. The intersection of both lines define T_{cross}. The temperatures of 25 K (blue), 50 K (green), 100 K (brown), 200 K (red) and 350 K (purple) have been marked as vertical dashed lines, where previously reports have observed transitions in bulk 1T-TaS_2 (see main text).

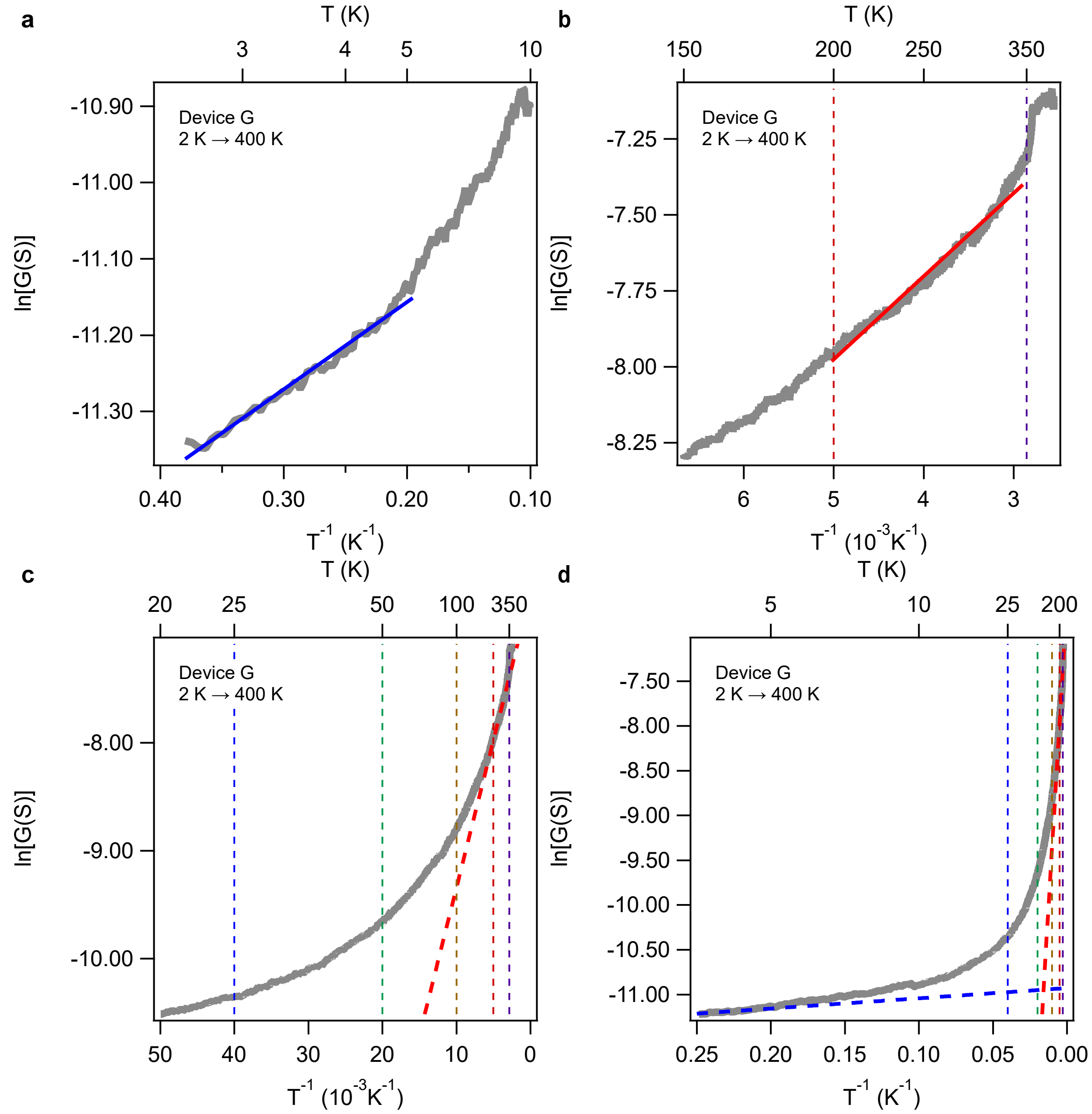

Figure S.22.- Arrhenius plot of the conductance (Device G) while sweeping the temperature from 2 K to 400 K at 1 K/min. a) Low-temperature regime with the fit shown in blue. b) High-temperature regime with the fit shown in red. c) Intermediate region and d) a broader temperature range where the previous fit values are prolonged in blue and red dashed lines. The intersection of both lines define T_{cross}. The temperatures of 25 K (blue), 50 K (green), 100 K (brown), 200 K (red) and 350 K (purple) have been marked as vertical dashed lines, where previously reports have observed transitions in bulk 1T-TaS_2 (see main text).

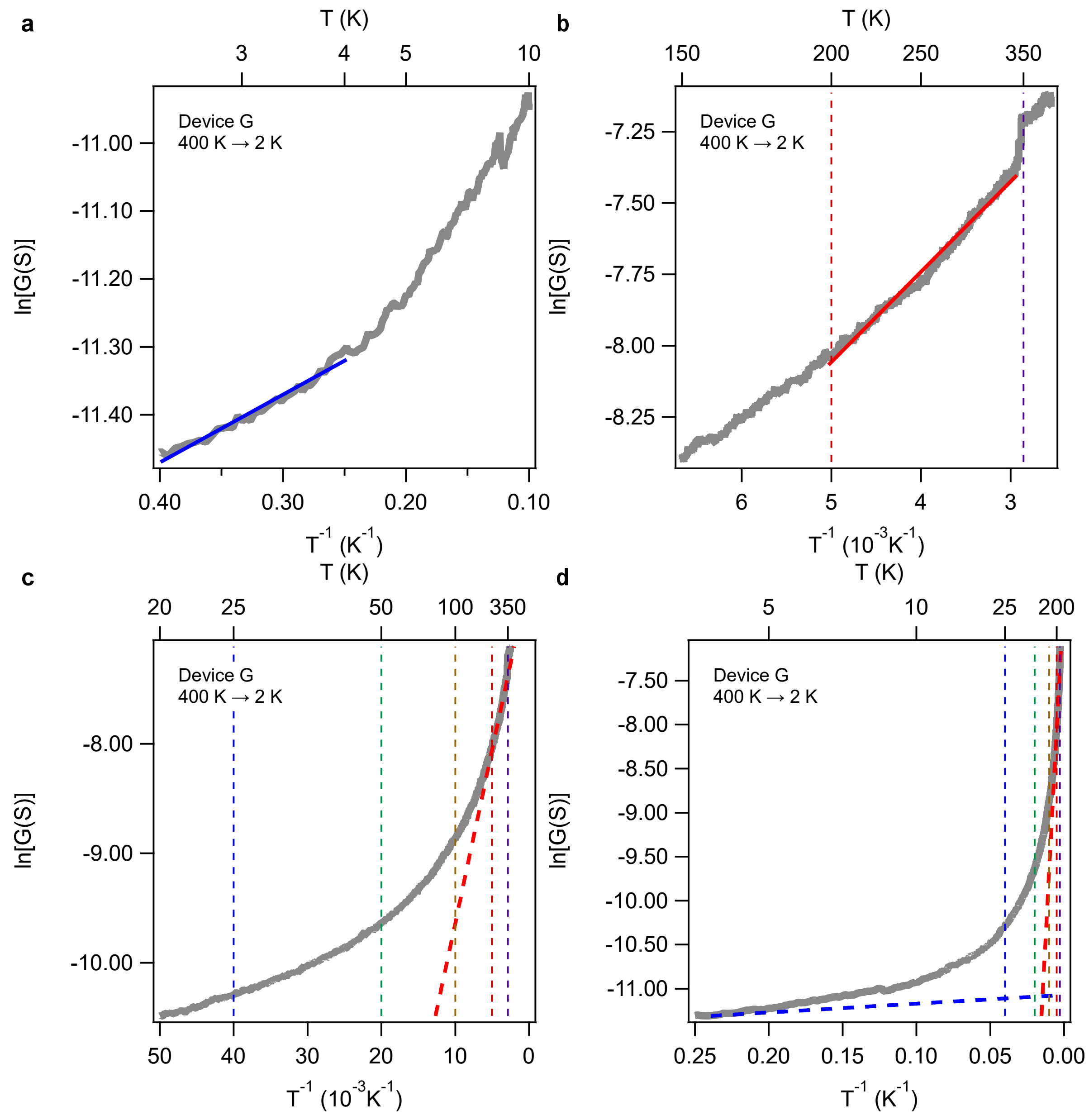

Figure S.23.- Arrhenius plot of the conductance (Device G) while sweeping the temperature from 400 K to 2 K at 1 K/min. a) Low-temperature regime with the fit shown in blue. b) High-temperature regime with the fit shown in red. c) Intermediate region and d) a broader temperature range where the previous fit values are prolonged in blue and red dashed lines. The intersection of both lines define T_{cross}. The temperatures of 25 K (blue), 50 K (green), 100 K (brown), 200 K (red) and 350 K (purple) have been marked as vertical dashed lines, where previously reports have observed transitions in bulk 1T-TaS_2 (see main text).

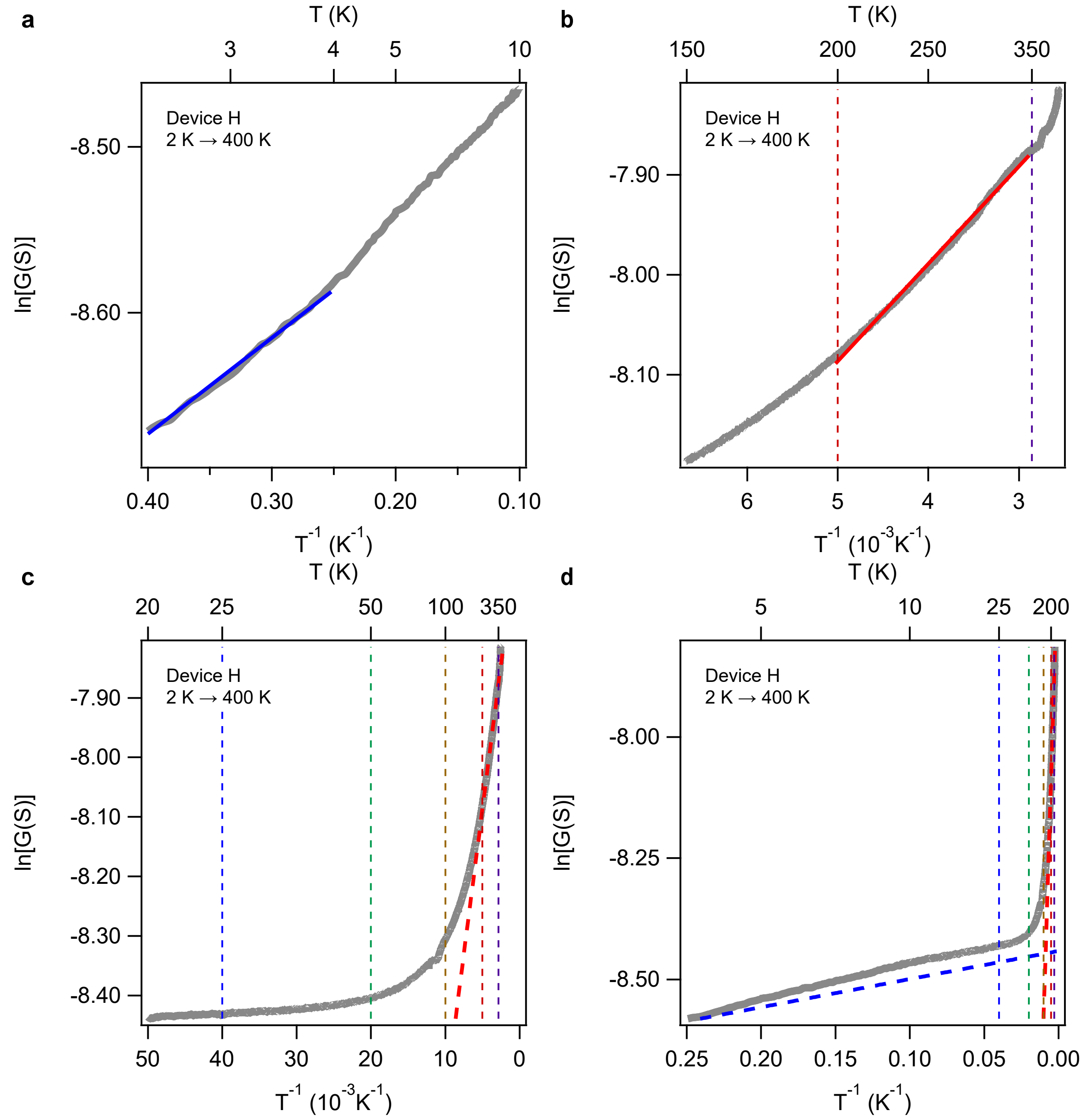

Figure S.24.- Arrhenius plot of the conductance (Device H) while sweeping the temperature from 2 K to 400 K at 1 K/min. a) Low-temperature regime with the fit shown in blue. b) High-temperature regime with the fit shown in red. c) Intermediate region and d) a broader temperature range where the previous fit values are prolonged in blue and red dashed lines. The intersection of both lines define T_{cross}. The temperatures of 25 K (blue), 50 K (green), 100 K (brown), 200 K (red) and 350 K (purple) have been marked as vertical dashed lines, where previously reports have observed transitions in bulk 1T-TaS_2 (see main text).

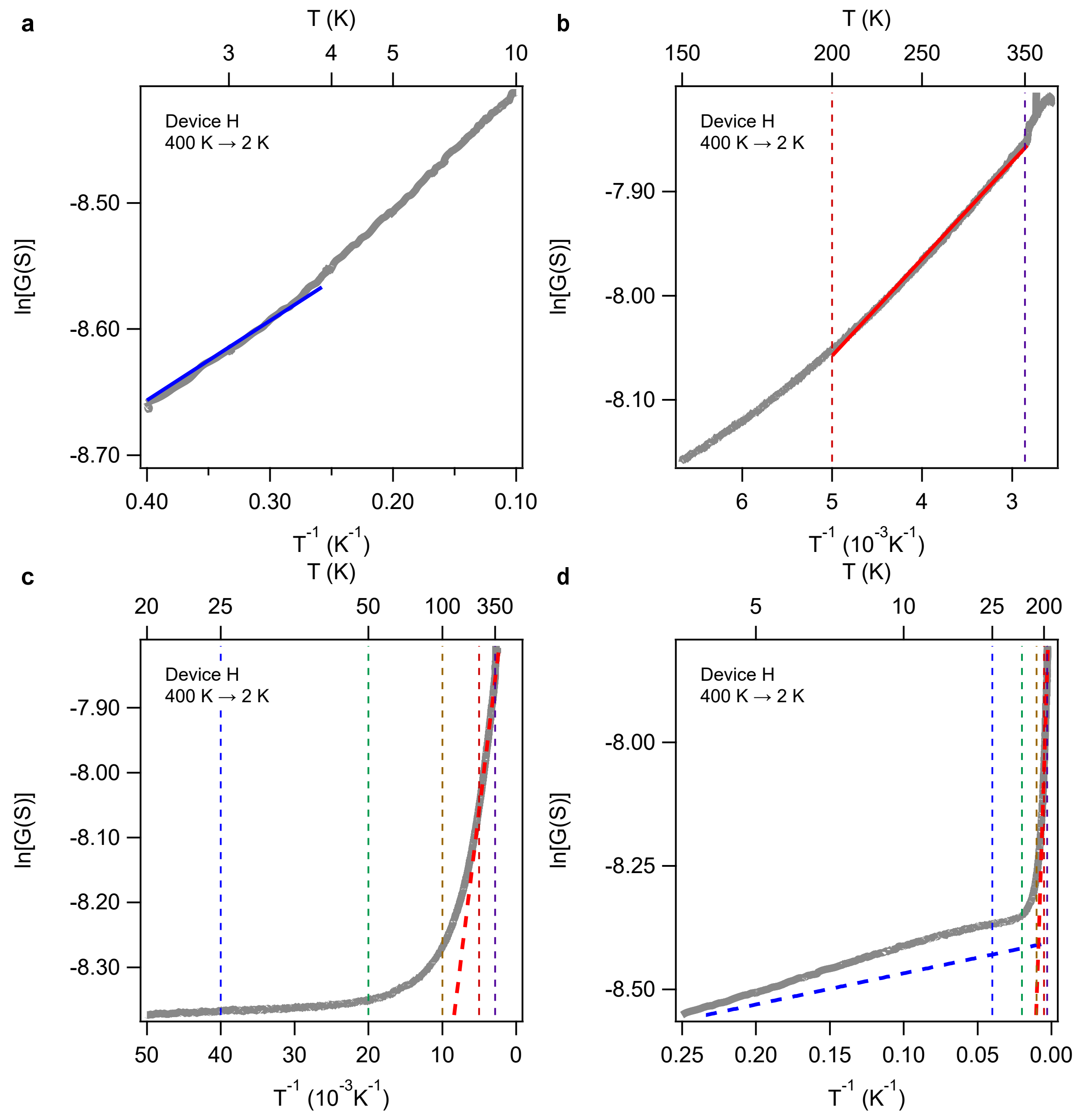

Figure S.25.- Arrhenius plot of the conductance (Device H) while sweeping the temperature from 400 K to 2 K at 1 K/min. a) Low-temperature regime with the fit shown in blue. b) High-temperature regime with the fit shown in red. c) Intermediate region and d) a broader temperature range where the previous fit values are prolonged in blue and red dashed lines. The intersection of both lines define T_{cross}. The temperatures of 25 K (blue), 50 K (green), 100 K (brown), 200 K (red) and 350 K (purple) have been marked as vertical dashed lines, where previously reports have observed transitions in bulk 1T-TaS_2 (see main text).

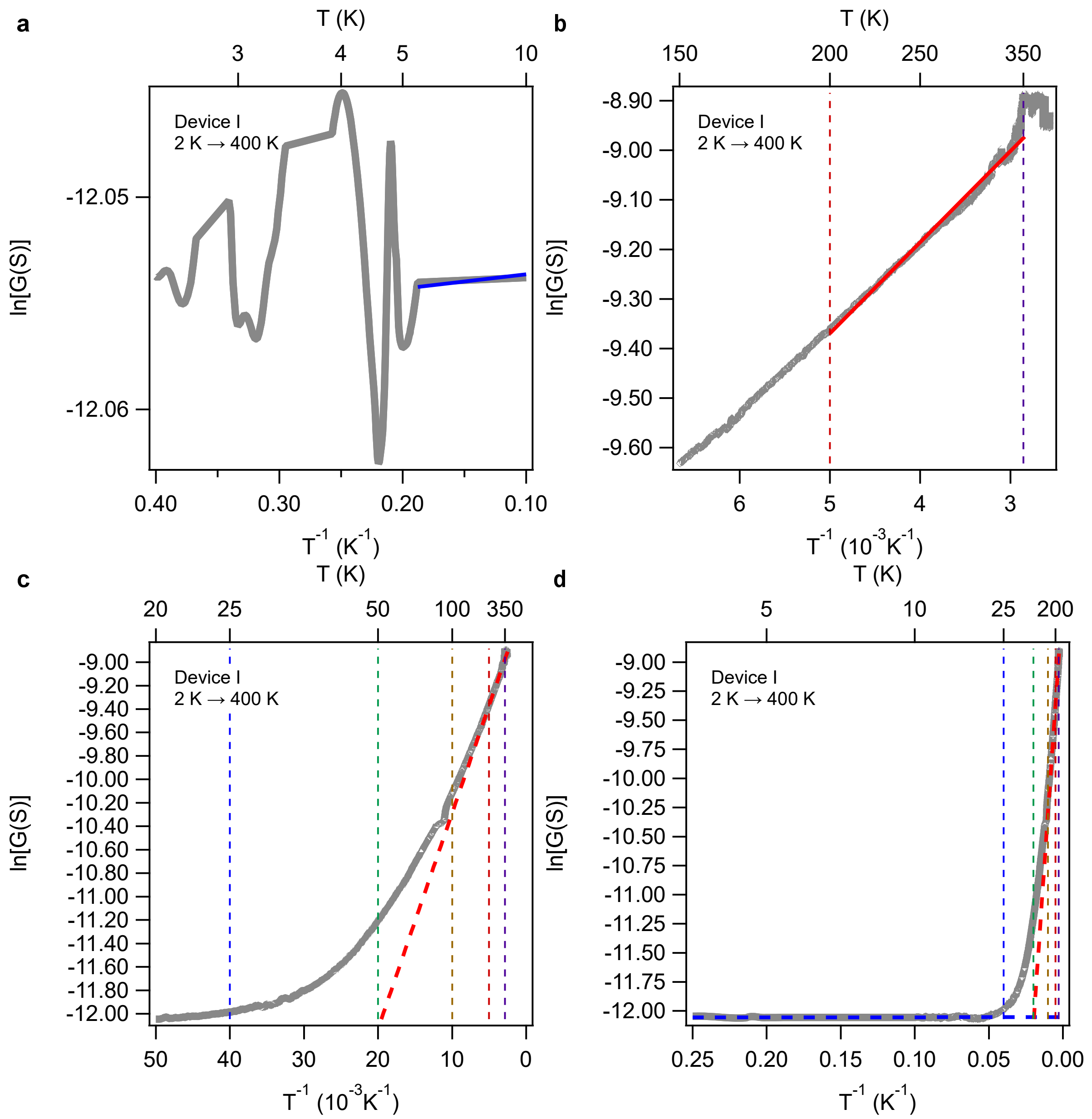

Figure S.26.- Arrhenius plot of the conductance (Device I) while sweeping the temperature from 2 K to 400 K at 1 K/min. a) Low-temperature regime with the fit shown in blue. b) High-temperature regime with the fit shown in red. c) Intermediate region and d) a broader temperature range where the previous fit values are prolonged in blue and red dashed lines. The intersection of both lines define T_{cross}. The temperatures of 25 K (blue), 50 K (green), 100 K (brown), 200 K (red) and 350 K (purple) have been marked as vertical dashed lines, where previously reports have observed transitions in bulk 1T-TaS_2 (see main text).

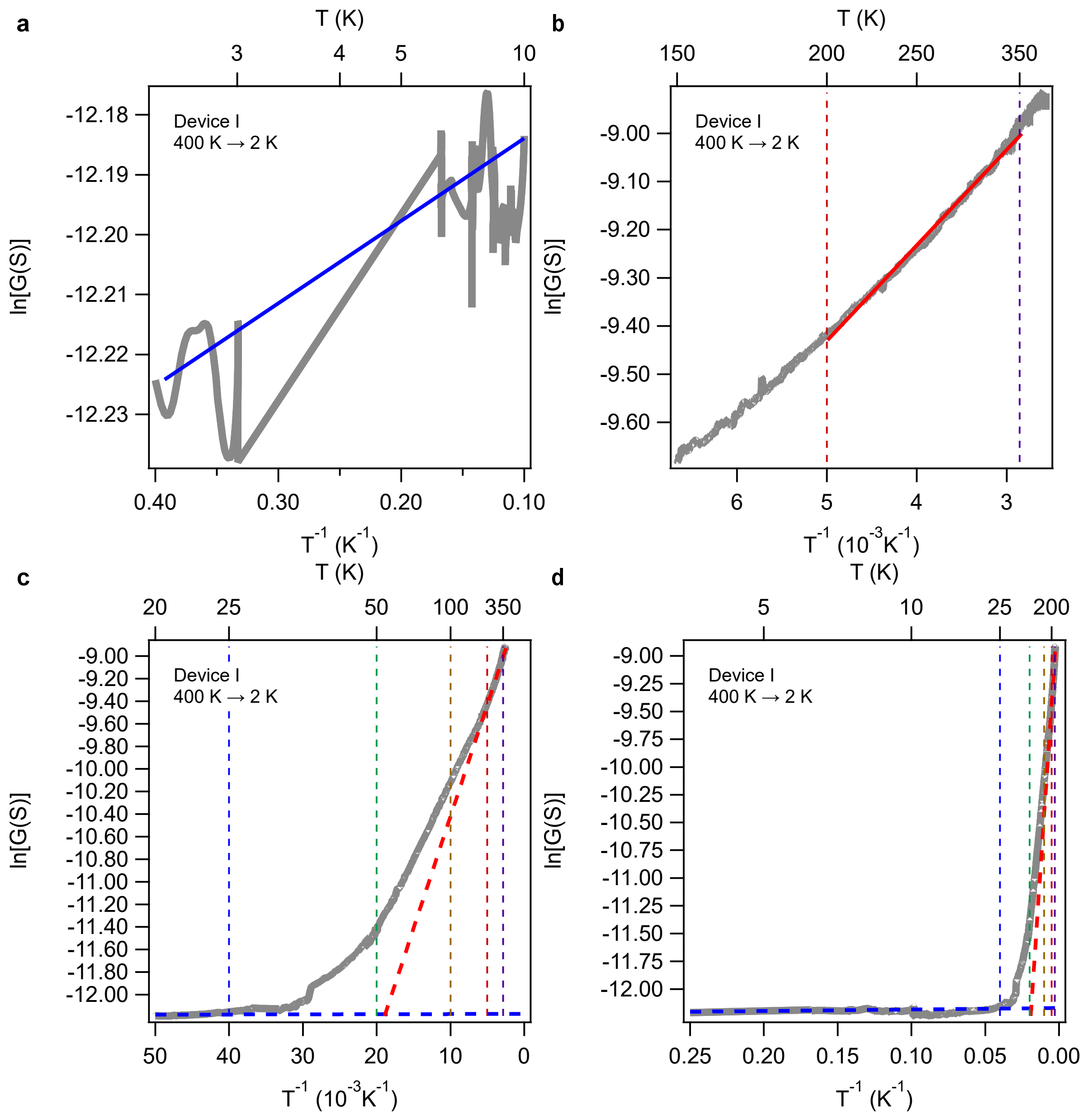

Figure S.27.- Arrhenius plot of the conductance (Device H) while sweeping the temperature from 400 K to 2 K at 1 K/min. a) Low-temperature regime with the fit shown in blue. b) High-temperature regime with the fit shown in red. c) Intermediate region and d) a broader temperature range where the previous fit values are prolonged in blue and red dashed lines. The intersection of both lines define T_{cross}. The temperatures of 25 K (blue), 50 K (green), 100 K (brown), 200 K (red) and 350 K (purple) have been marked as vertical dashed lines, where previously reports have observed transitions in bulk 1T-TaS_2 (see main text).

3.3.- Hopping analysis.

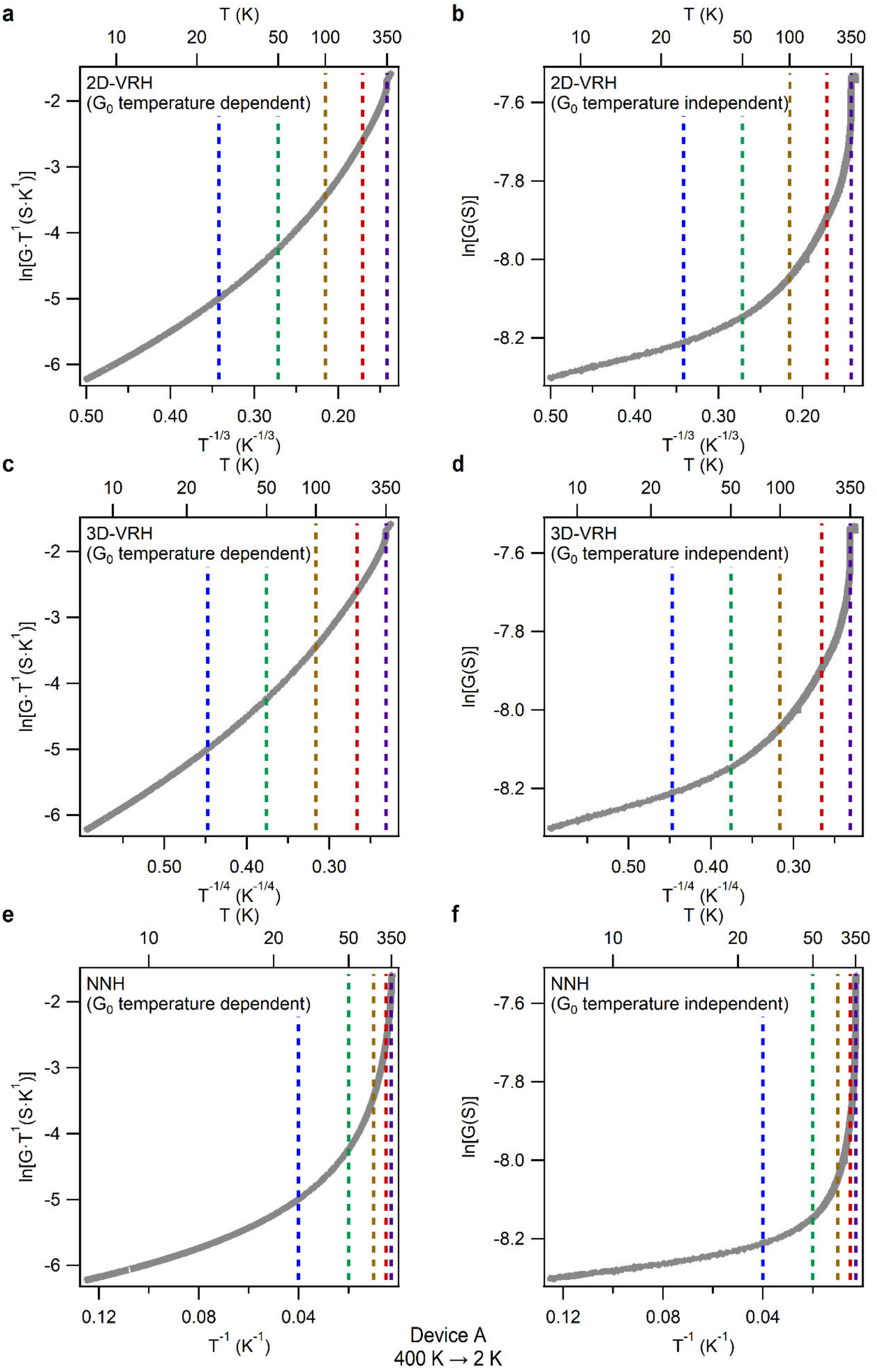

Figure S. 28.- VRH plots of the conductance. The temperatures of 25 K (blue), 50 K (green), 100 K (brown), 200 K (red) and 350 K (purple) have been marked as vertical dashed lines, where previously reports have observed transitions in bulk 1T-TaS_2 (see main text).

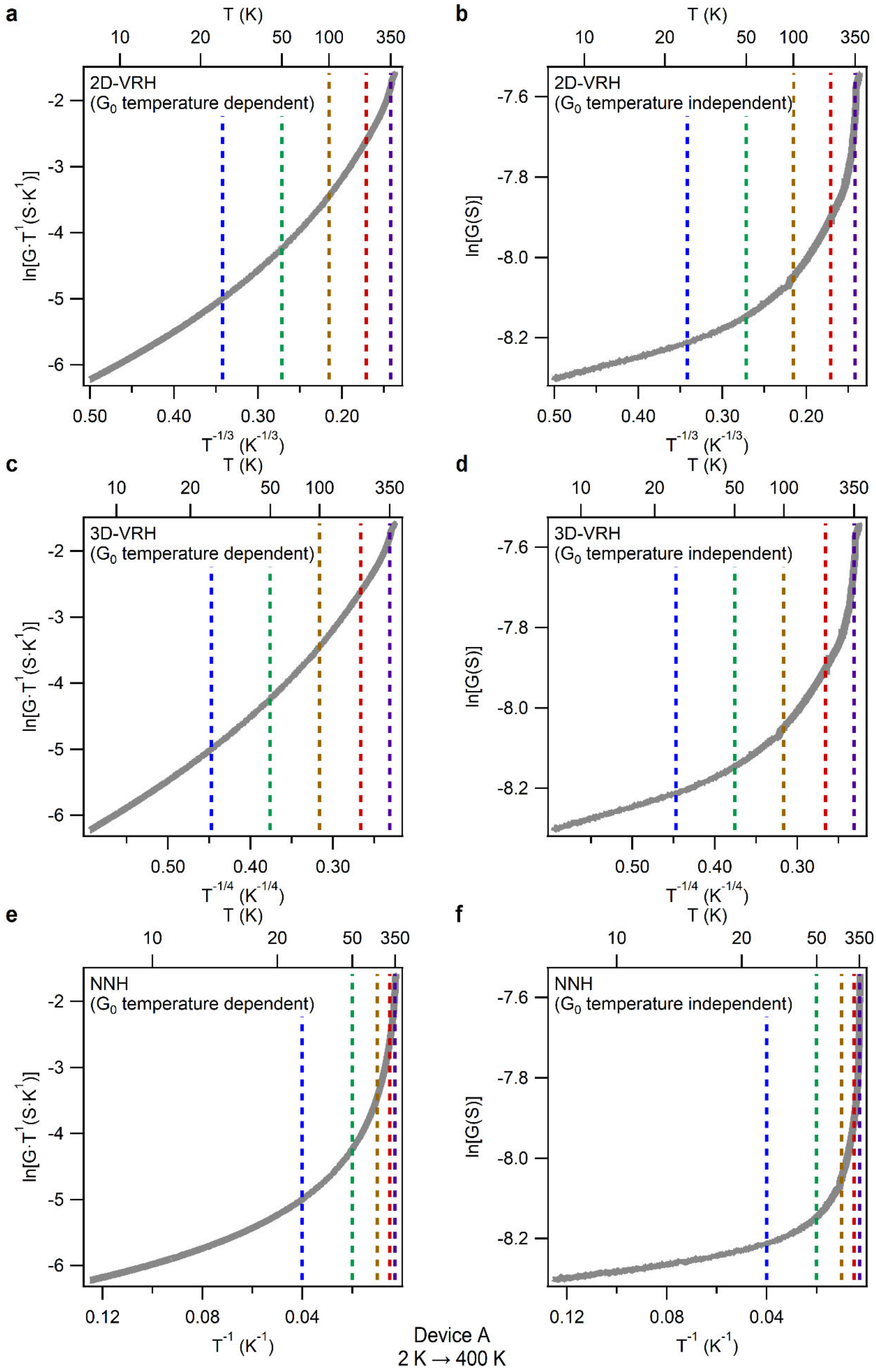

Figure S. 29.- VRH plots of the conductance. The temperatures of 25 K (blue), 50 K (green), 100 K (brown), 200 K (red) and 350 K (purple) have been marked as vertical dashed lines, where previously reports have observed transitions in bulk 1T-TaS_2 (see main text).

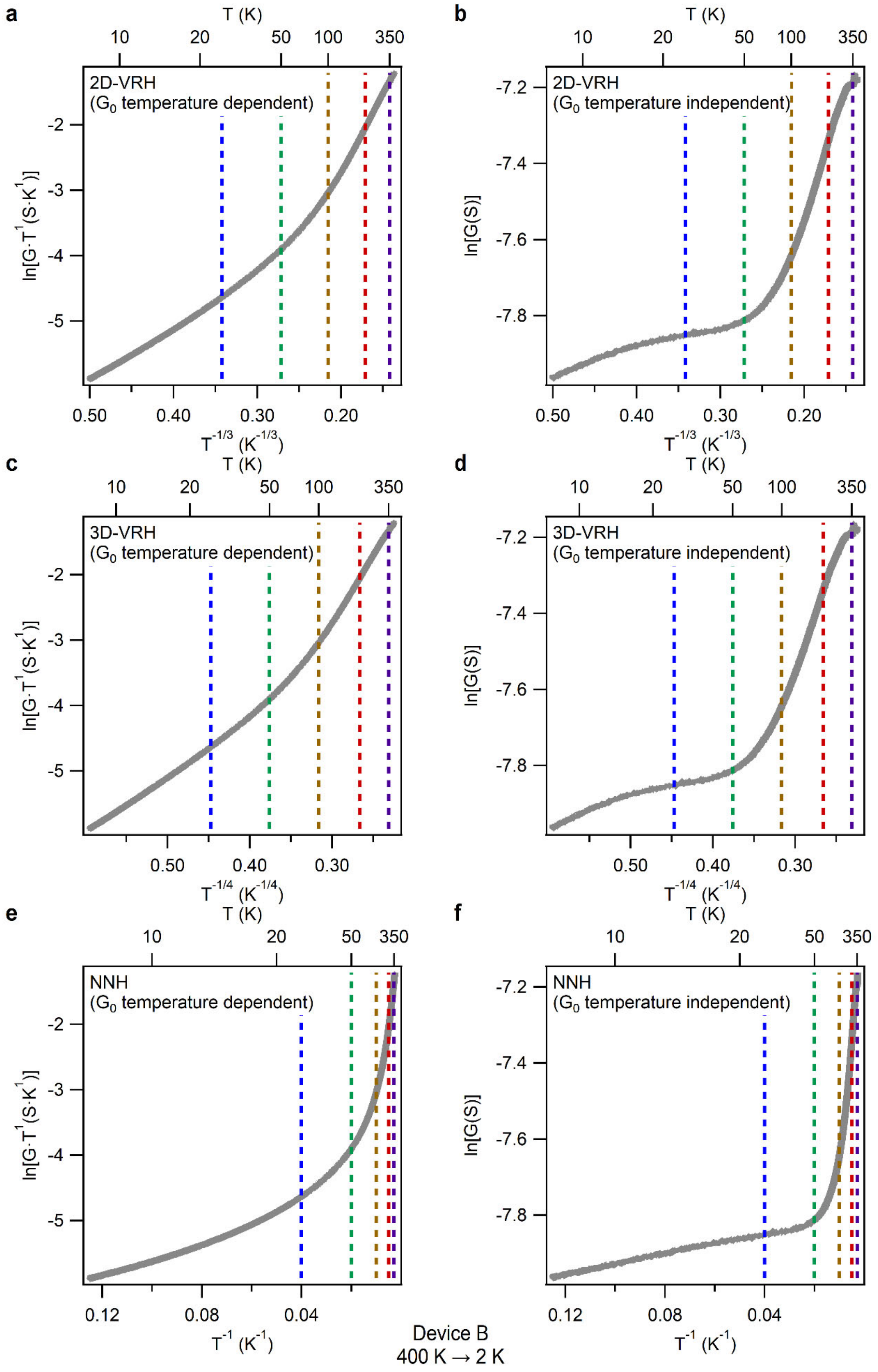

Figure S. 30.- VRH plots of the conductance. The temperatures of 25 K (blue), 50 K (green), 100 K (brown), 200 K (red) and 350 K (purple) have been marked as vertical dashed lines, where previously reports have observed transitions in bulk 1T-TaS_2 (see main text).

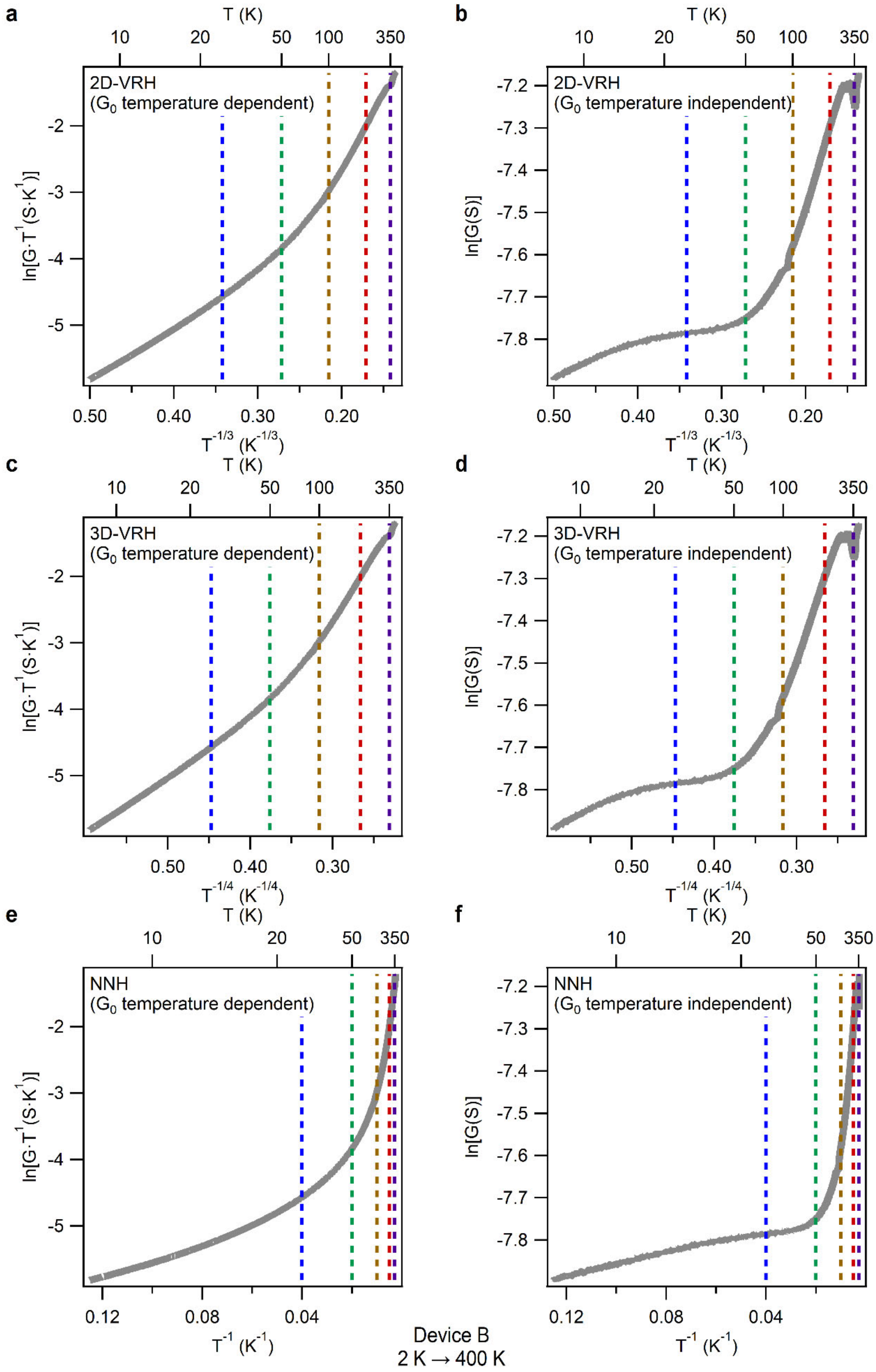

Figure S. 31.- VRH plots of the conductance. The temperatures of 25 K (blue), 50 K (green), 100 K (brown), 200 K (red) and 350 K (purple) have been marked as vertical dashed lines, where previously reports have observed transitions in bulk 1T-TaS_2 (see main text).

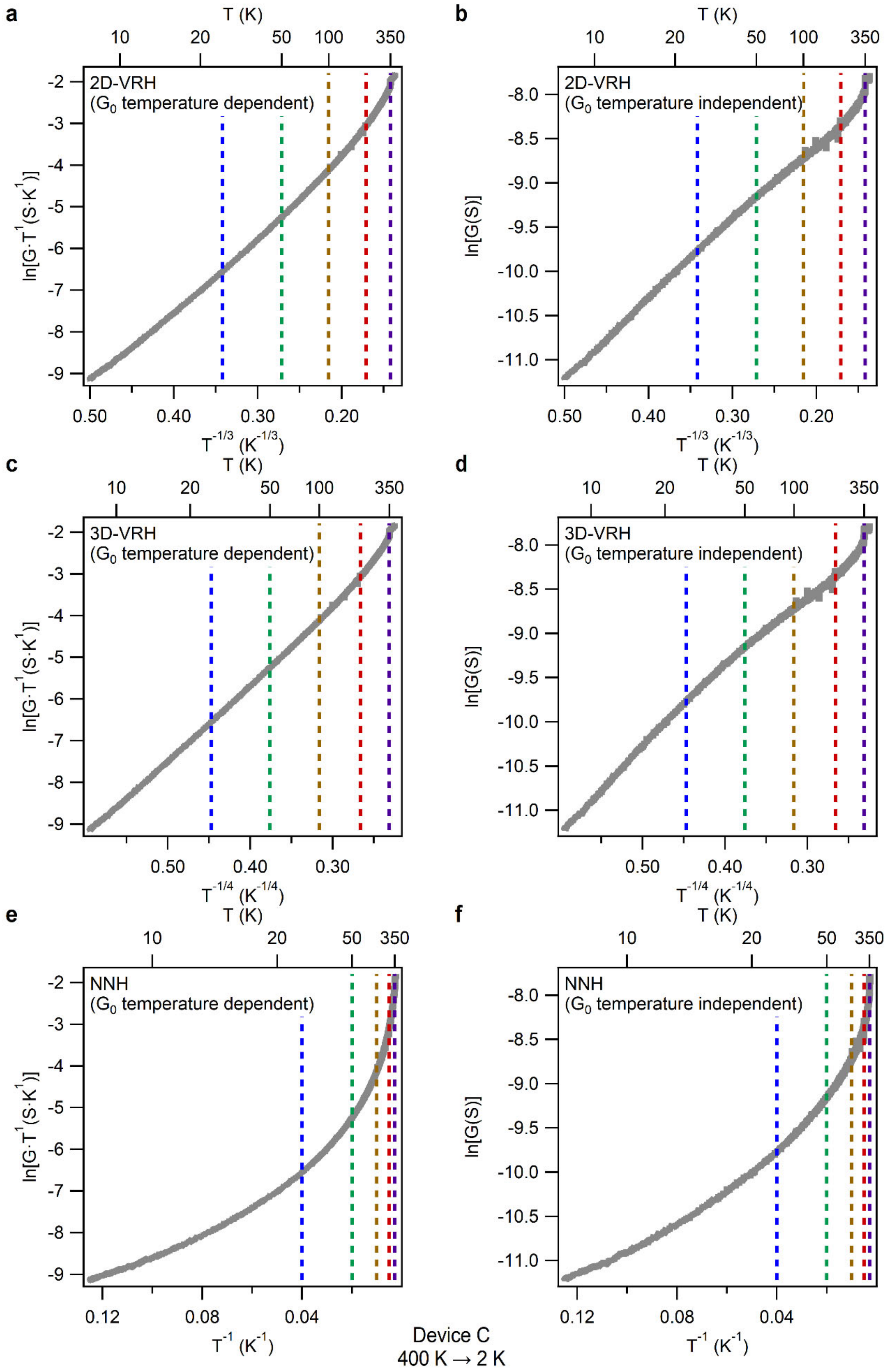

Figure S. 32.- VRH plots of the conductance. The temperatures of 25 K (blue), 50 K (green), 100 K (brown), 200 K (red) and 350 K (purple) have been marked as vertical dashed lines, where previously reports have observed transitions in bulk 1T-TaS_2 (see main text).

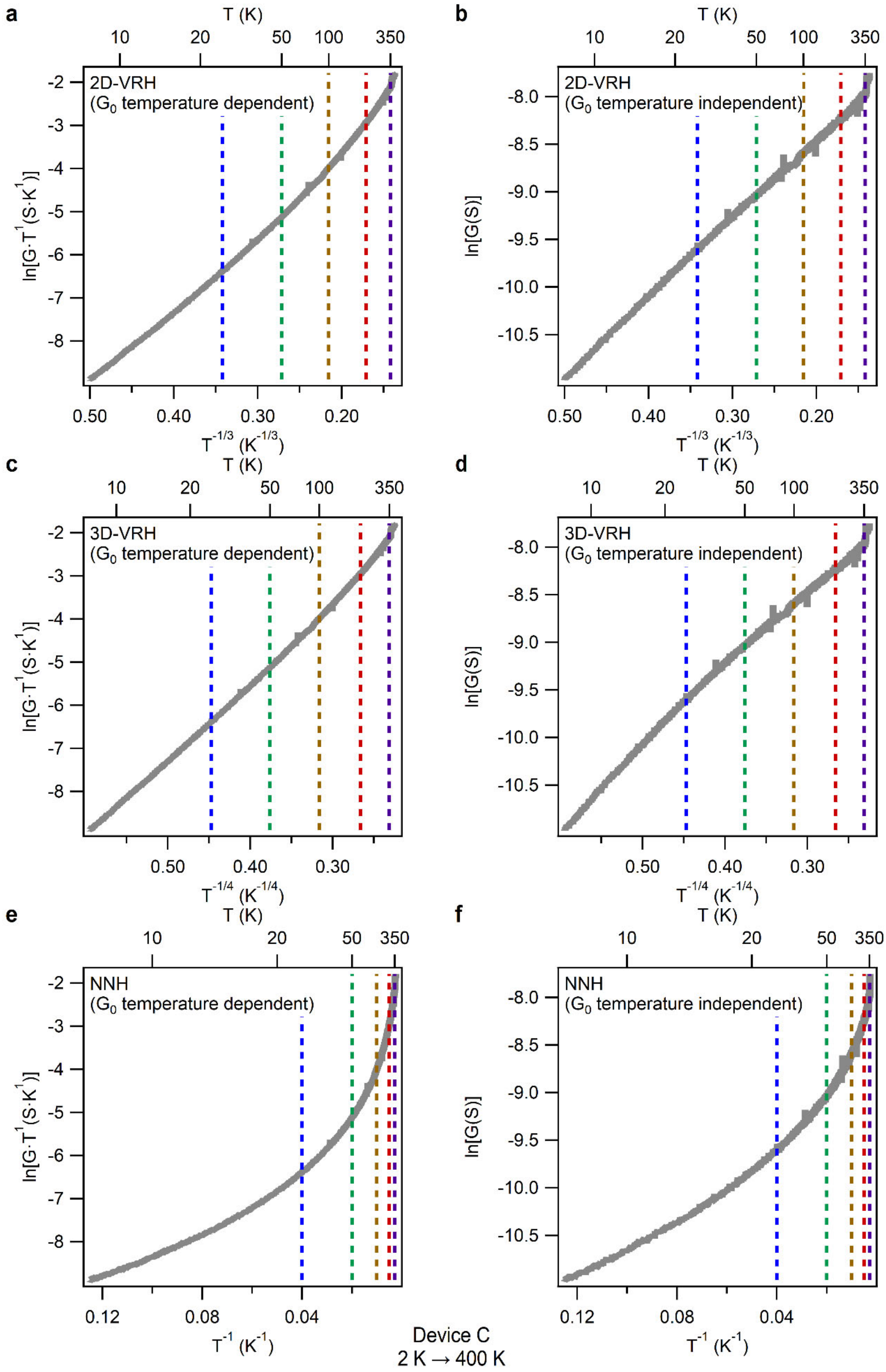

Figure S. 33.- VRH plots of the conductance. The temperatures of 25 K (blue), 50 K (green), 100 K (brown), 200 K (red) and 350 K (purple) have been marked as vertical dashed lines, where previously reports have observed transitions in bulk 1T-TaS_2 (see main text).

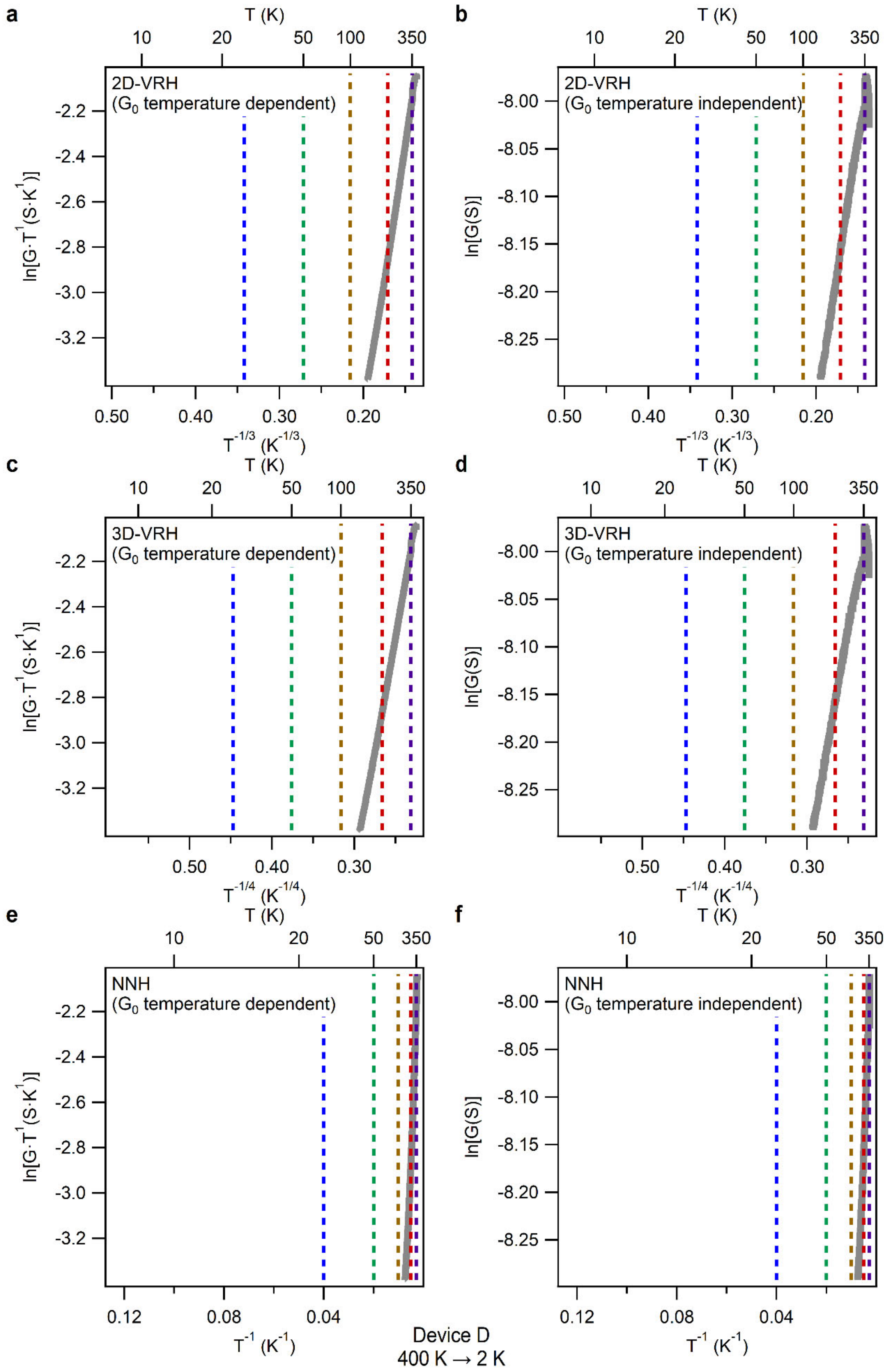

Figure S. 34.- VRH plots of the conductance. The temperatures of 25 K (blue), 50 K (green), 100 K (brown), 200 K (red) and 350 K (purple) have been marked as vertical dashed lines, where previously reports have observed transitions in bulk 1T-TaS_2 (see main text).

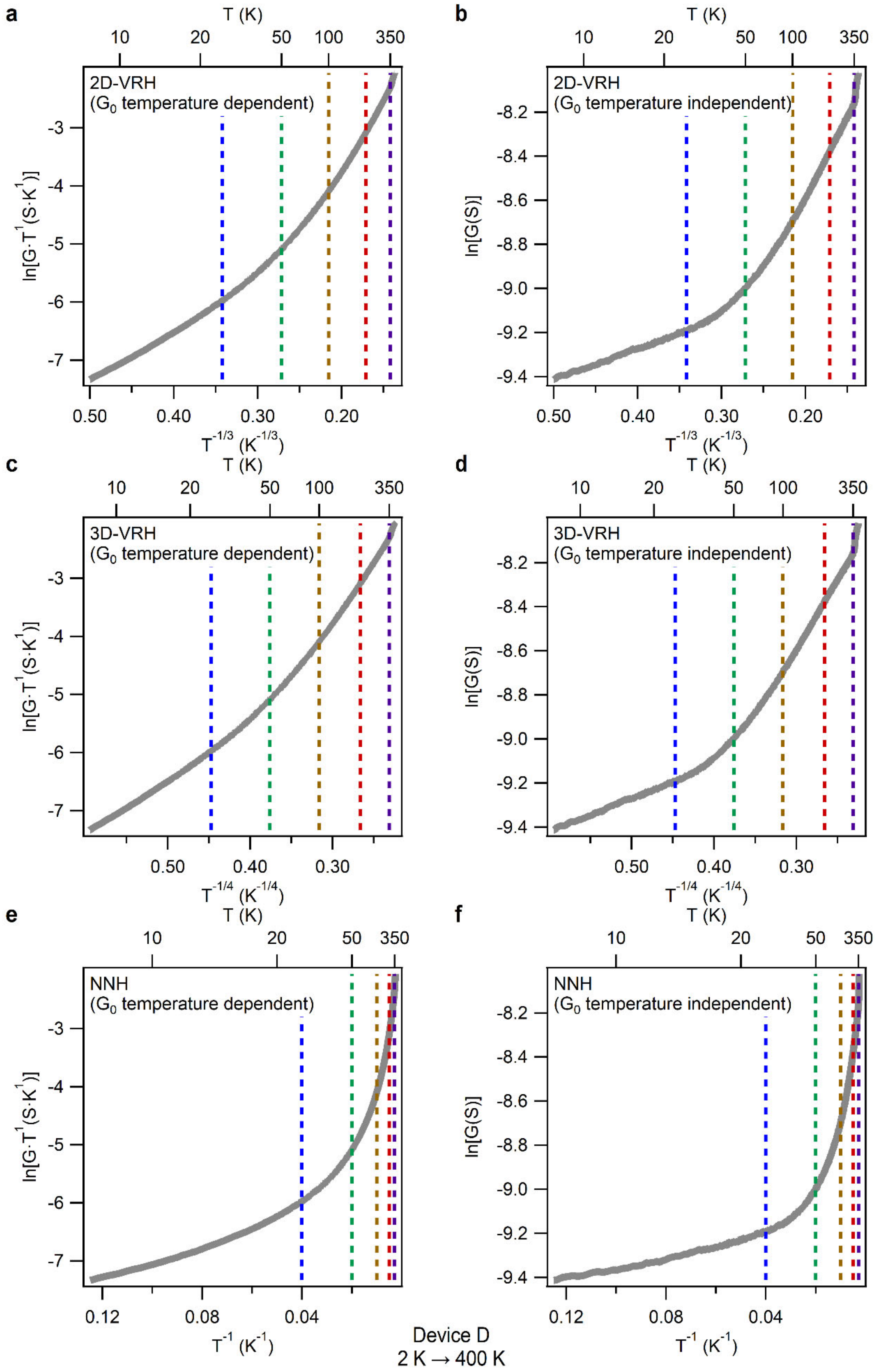

Figure S. 35.- VRH plots of the conductance. The temperatures of 25 K (blue), 50 K (green), 100 K (brown), 200 K (red) and 350 K (purple) have been marked as vertical dashed lines, where previously reports have observed transitions in bulk 1T-TaS_2 (see main text).

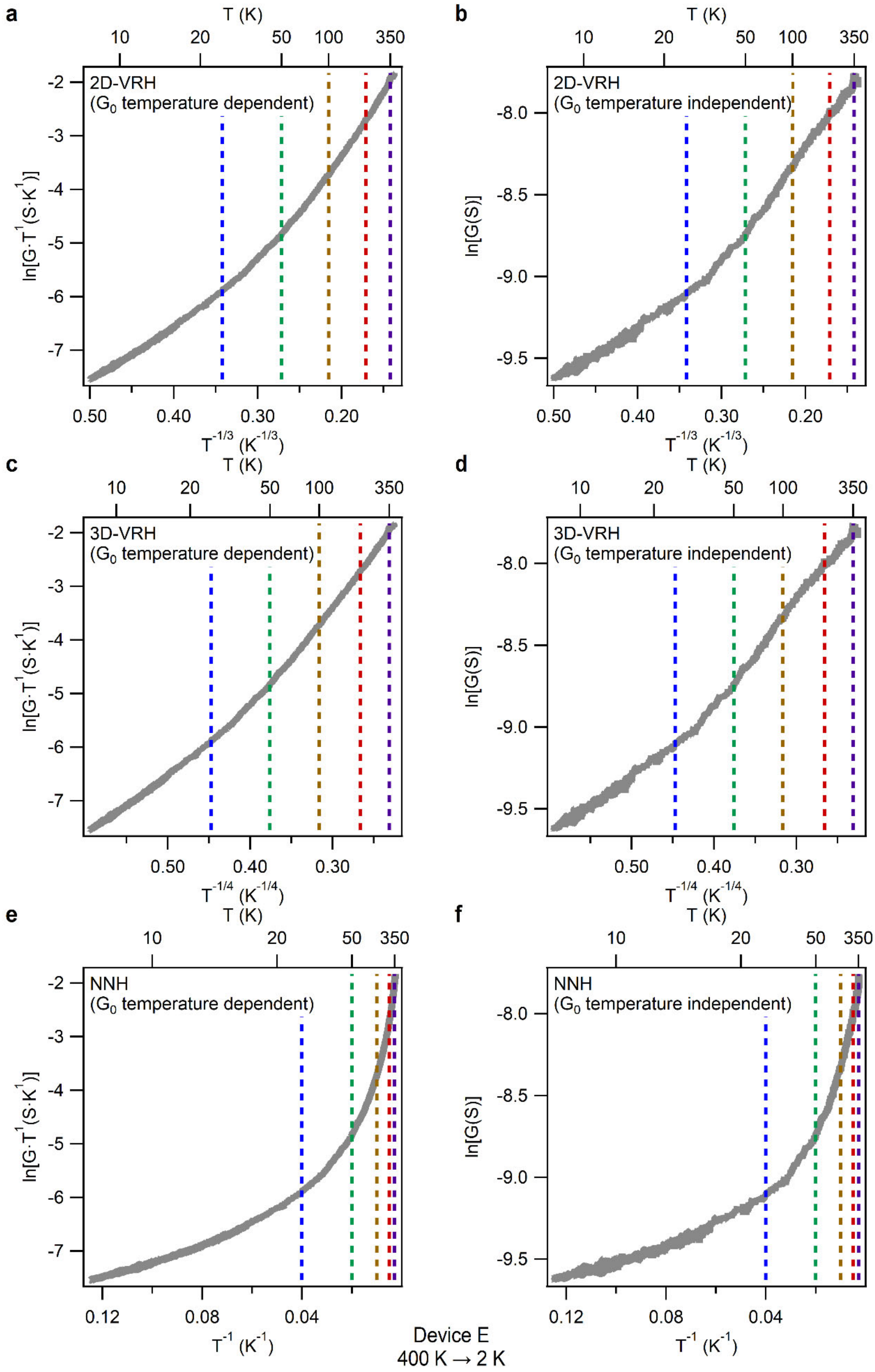

Figure S. 36.- VRH plots of the conductance. The temperatures of 25 K (blue), 50 K (green), 100 K (brown), 200 K (red) and 350 K (purple) have been marked as vertical dashed lines, where previously reports have observed transitions in bulk 1T-TaS2 (see main text).

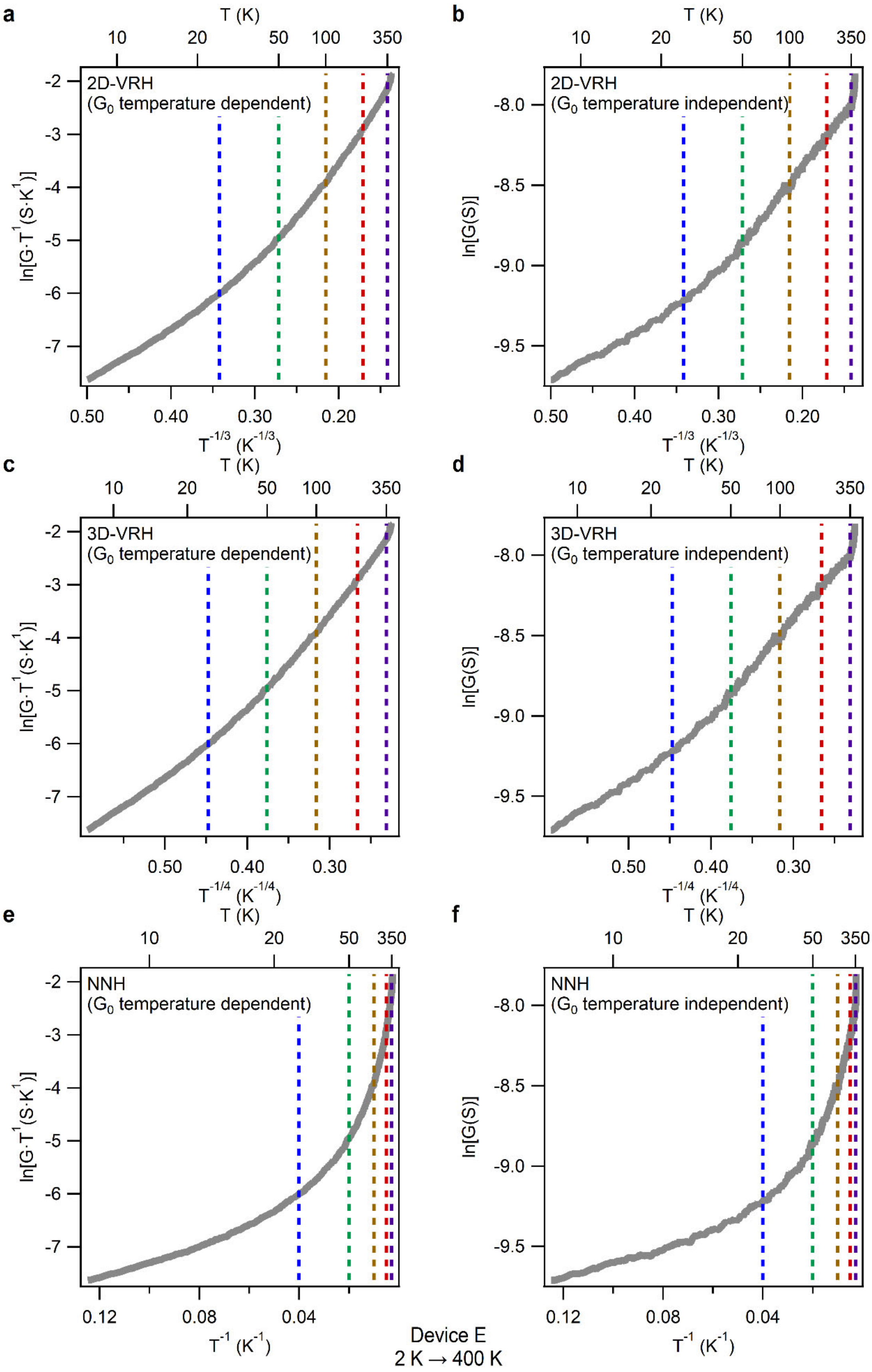

Figure S. 37.- VRH plots of the conductance. The temperatures of 25 K (blue), 50 K (green), 100 K (brown), 200 K (red) and 350 K (purple) have been marked as vertical dashed lines, where previously reports have observed transitions in bulk 1T-TaS_2 (see main text).

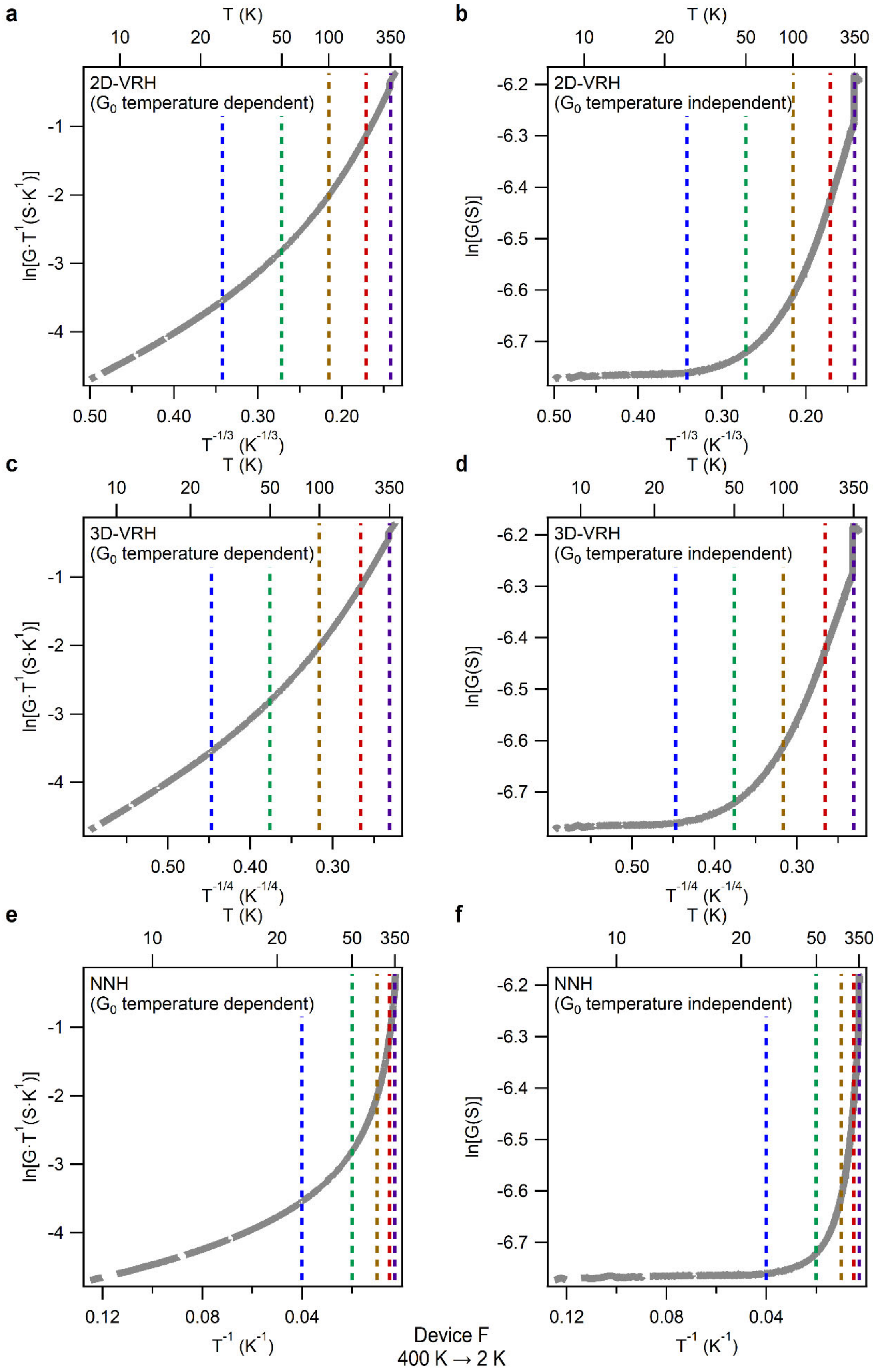

Figure S. 38.- VRH plots of the conductance. The temperatures of 25 K (blue), 50 K (green), 100 K (brown), 200 K (red) and 350 K (purple) have been marked as vertical dashed lines, where previously reports have observed transitions in bulk 1T-TaS_2 (see main text).

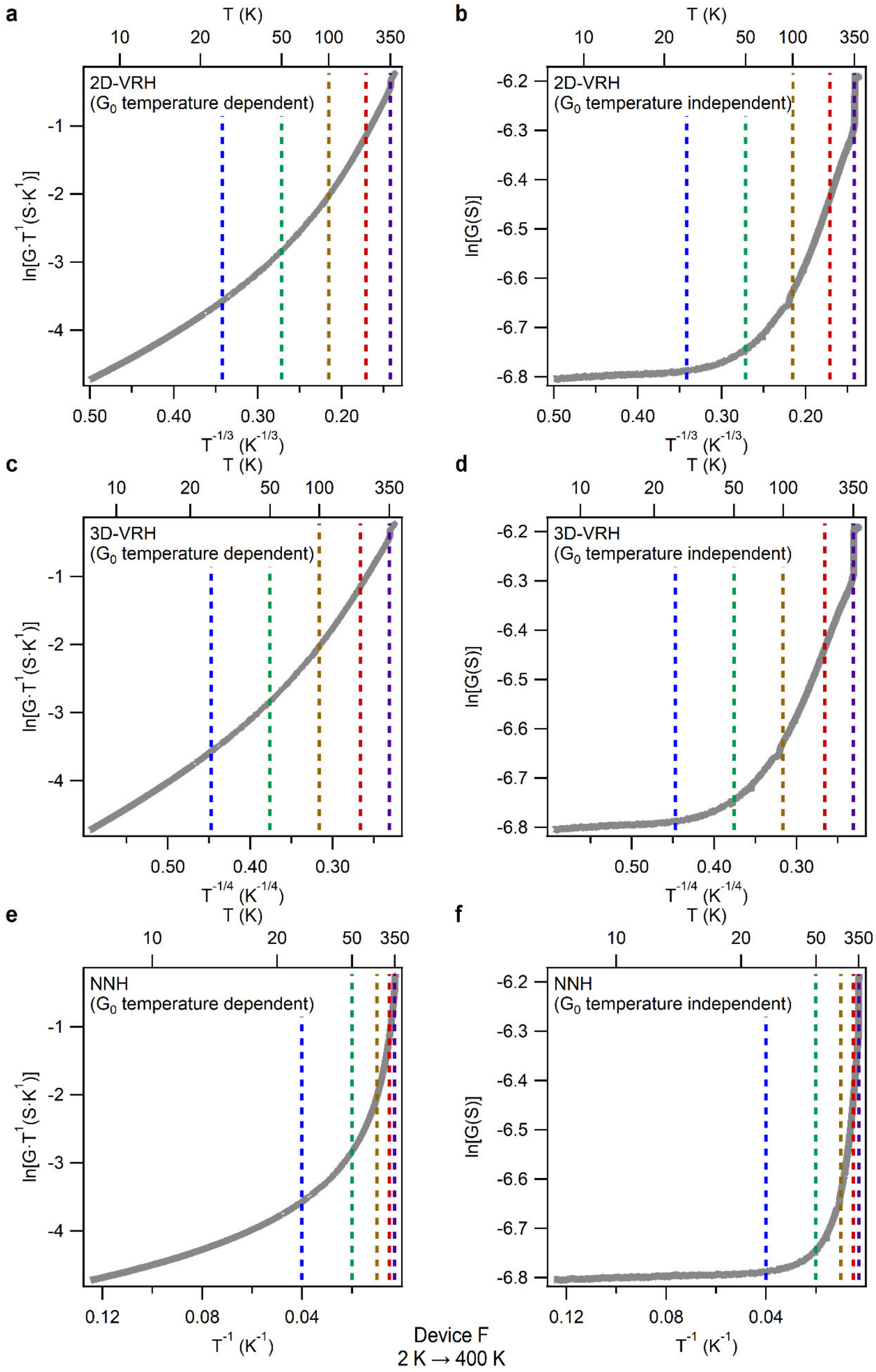

Figure S. 39.- VRH plots of the conductance. The temperatures of 25 K (blue), 50 K (green), 100 K (brown), 200 K (red) and 350 K (purple) have been marked as vertical dashed lines, where previously reports have observed transitions in bulk 1T-TaS_2 (see main text).

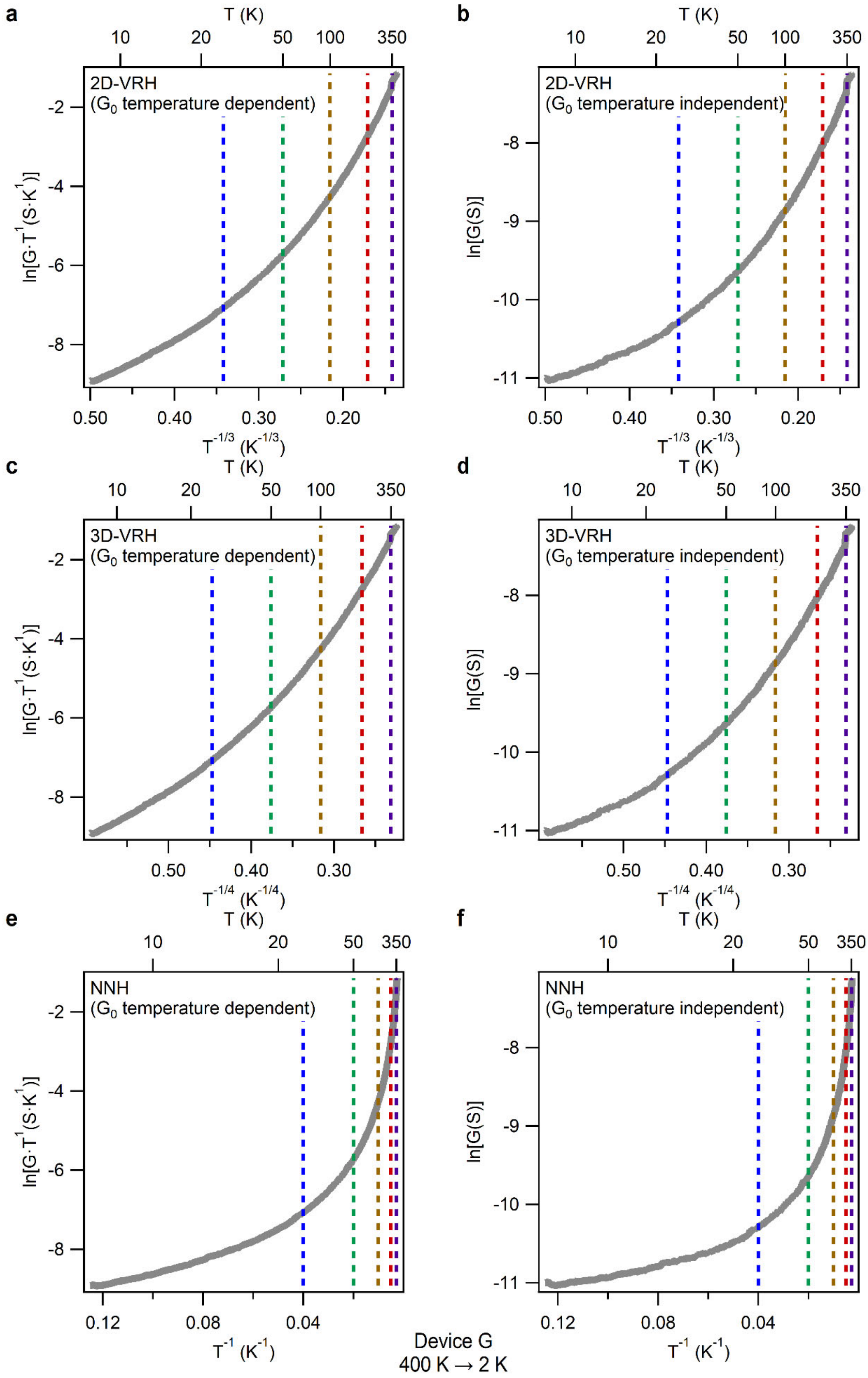

Figure S. 40.- VRH plots of the conductance. The temperatures of 25 K (blue), 50 K (green), 100 K (brown), 200 K (red) and 350 K (purple) have been marked as vertical dashed lines, where previously reports have observed transitions in bulk 1T-TaS_2 (see main text).

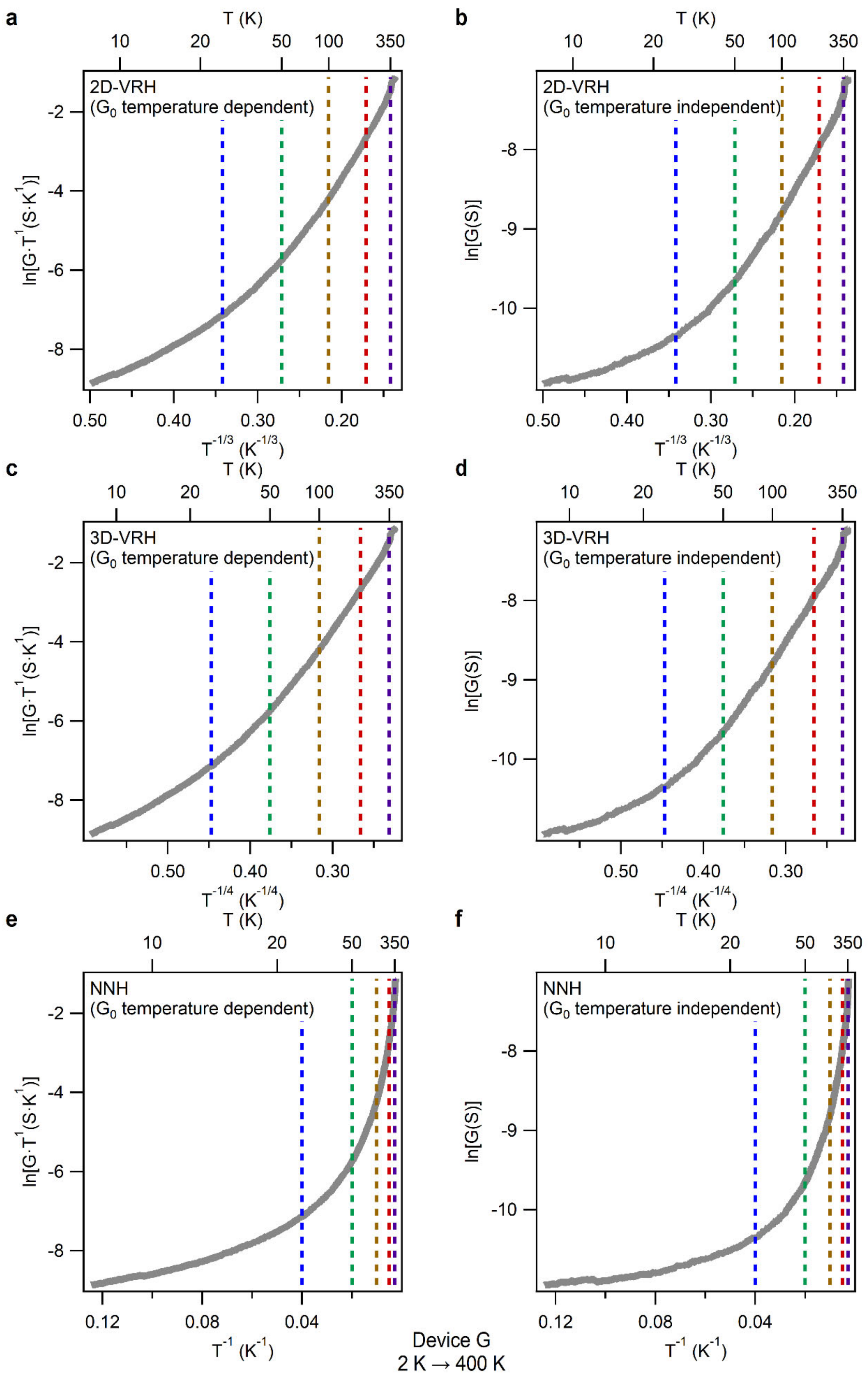

Figure S. 41.- VRH plots of the conductance. The temperatures of 25 K (blue), 50 K (green), 100 K (brown), 200 K (red) and 350 K (purple) have been marked as vertical dashed lines, where previously reports have observed transitions in bulk 1T-TaS_2 (see main text).

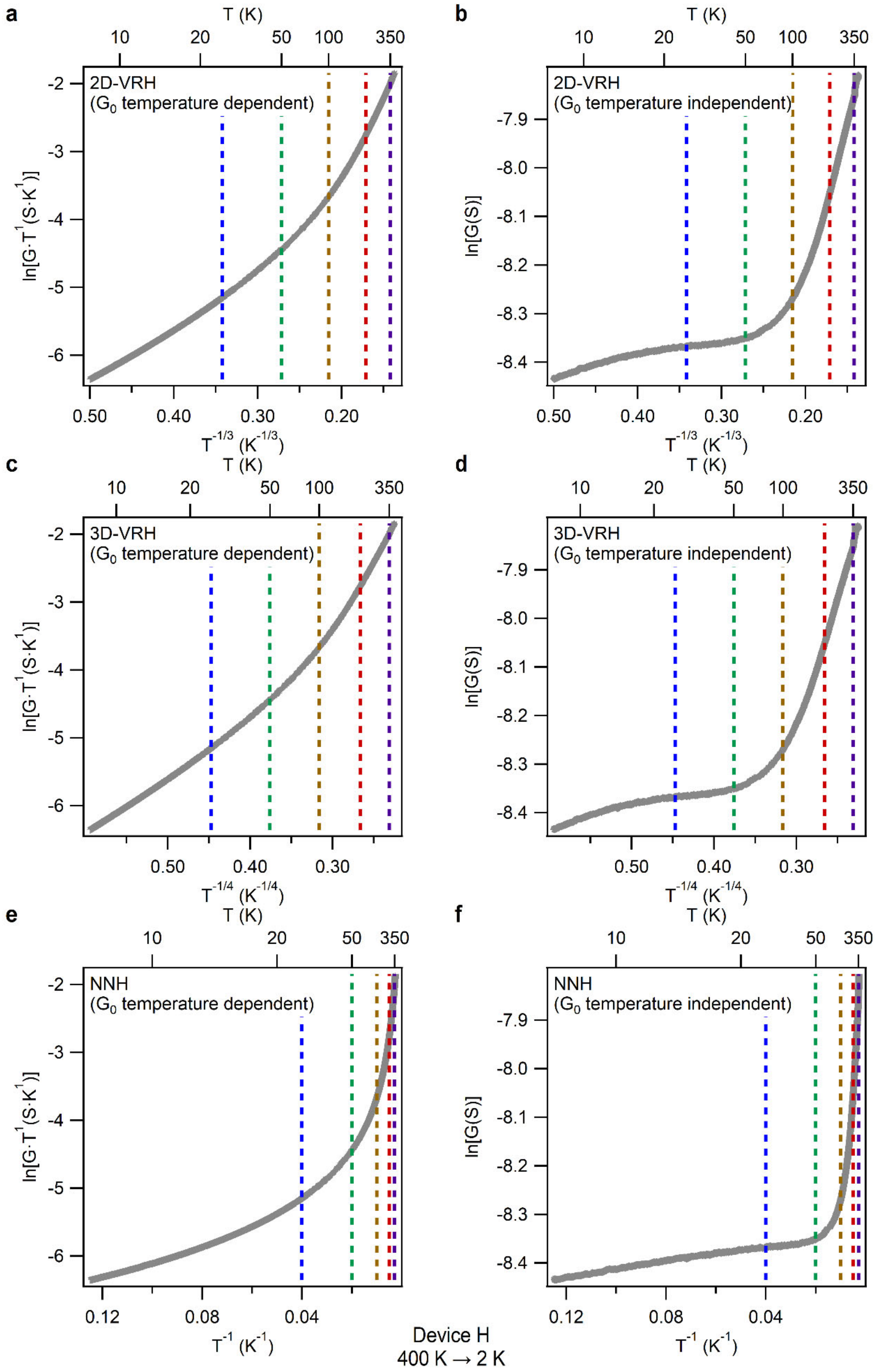

Figure S. 42.- VRH plots of the conductance. The temperatures of 25 K (blue), 50 K (green), 100 K (brown), 200 K (red) and 350 K (purple) have been marked as vertical dashed lines, where previously reports have observed transitions in bulk 1T-TaS_2 (see main text).

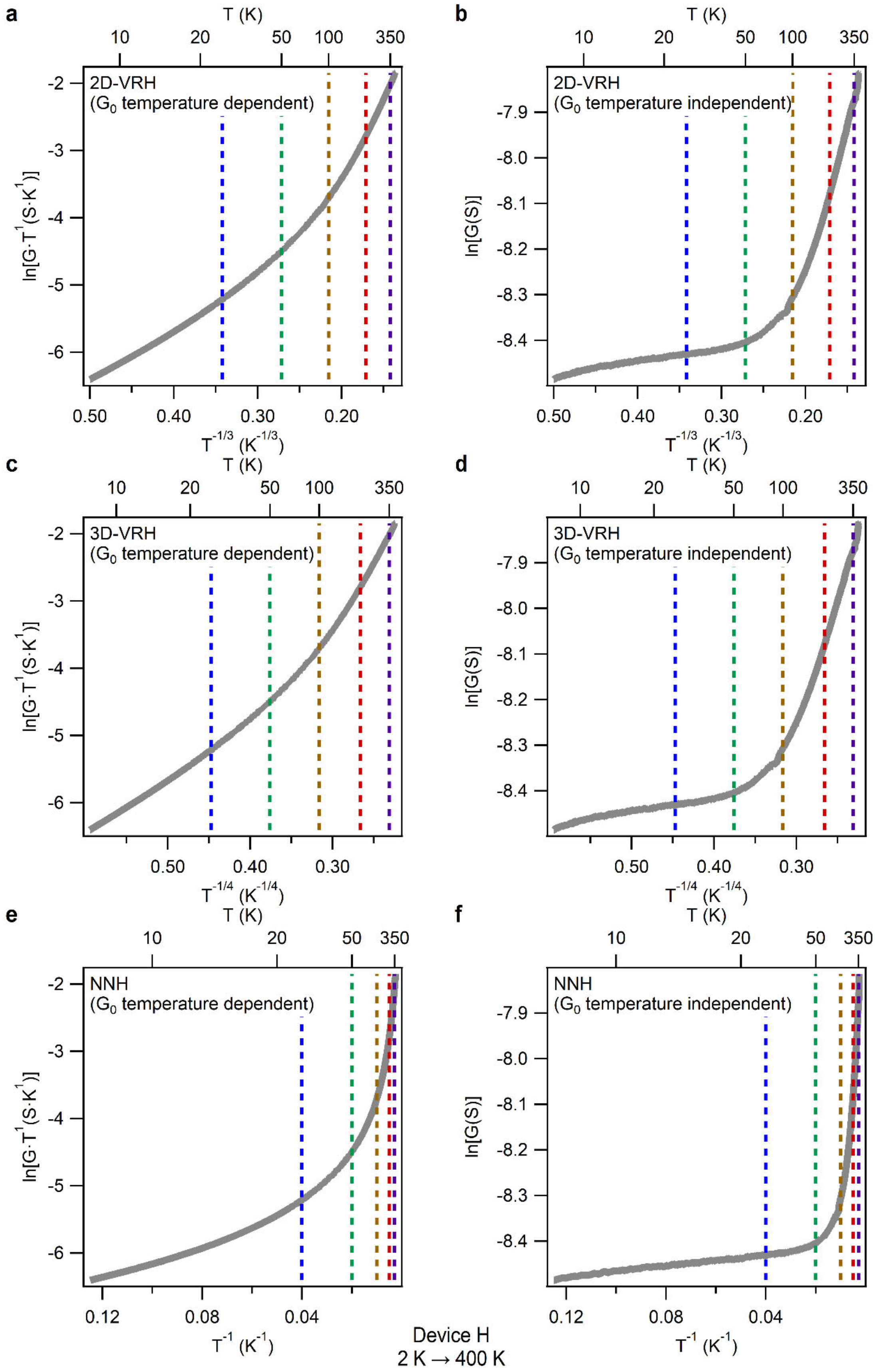

Figure S. 43.- VRH plots of the conductance. The temperatures of 25 K (blue), 50 K (green), 100 K (brown), 200 K (red) and 350 K (purple) have been marked as vertical dashed lines, where previously reports have observed transitions in bulk 1T-TaS_2 (see main text).

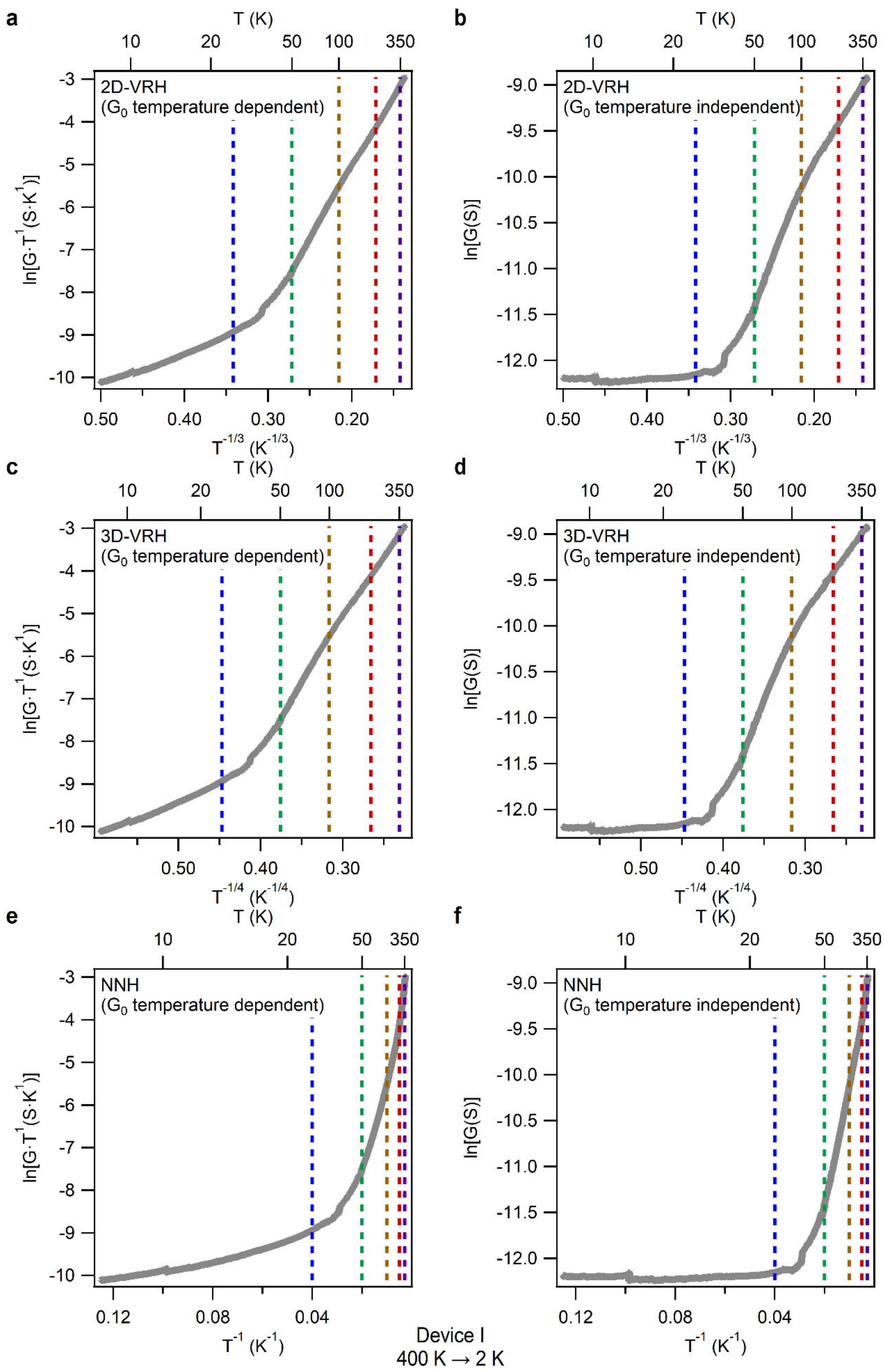

Figure S. 44.- VRH plots of the conductance. The temperatures of 25 K (blue), 50 K (green), 100 K (brown), 200 K (red) and 350 K (purple) have been marked as vertical dashed lines, where previously reports have observed transitions in bulk 1T-TaS_2 (see main text).

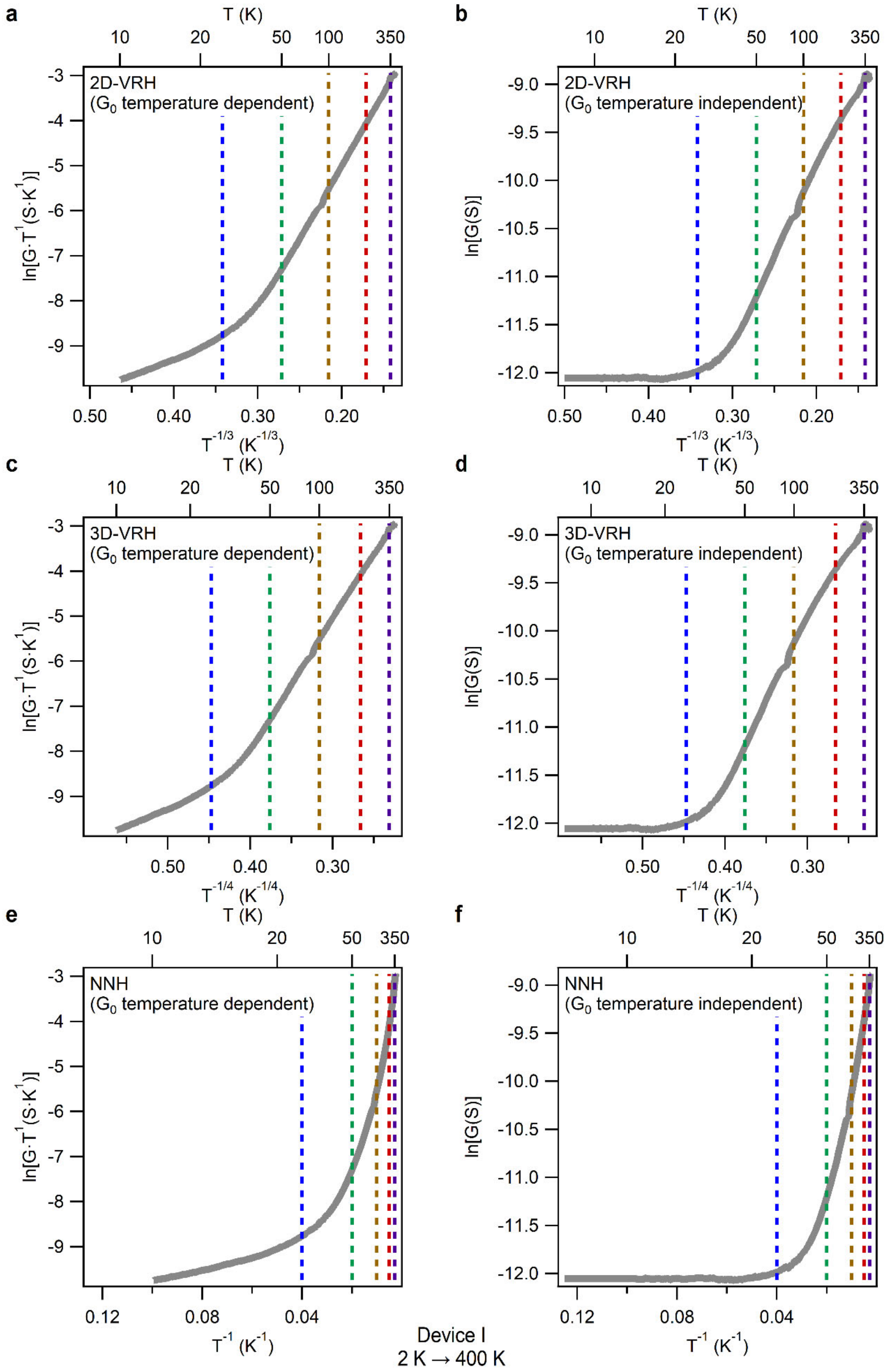

Figure S. 45.- VRH plots of the conductance. The temperatures of 25 K (blue), 50 K (green), 100 K (brown), 200 K (red) and 350 K (purple) have been marked as vertical dashed lines, where previously reports have observed transitions in bulk 1T-TaS_2 (see main text).

4. Measurements on the FLG/TaS_2/ FLG graphene devices.

4.1. Device A.

Device

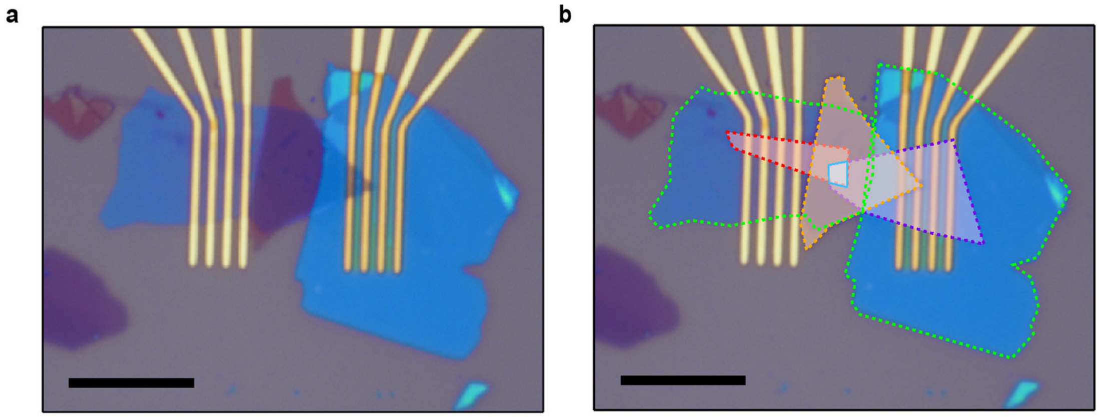

Figure S.46.- Optical picture of the device. For simplicity, the top graphite contact has been highlighted in red, the bottom graphite contact in purple and the 1T-TaS_2 barrier in orange. h-BN is marked in green. The junction (blue area) is the overlapped area formed by the three different materials. Scale bar: 10 µm.

Transport measurements

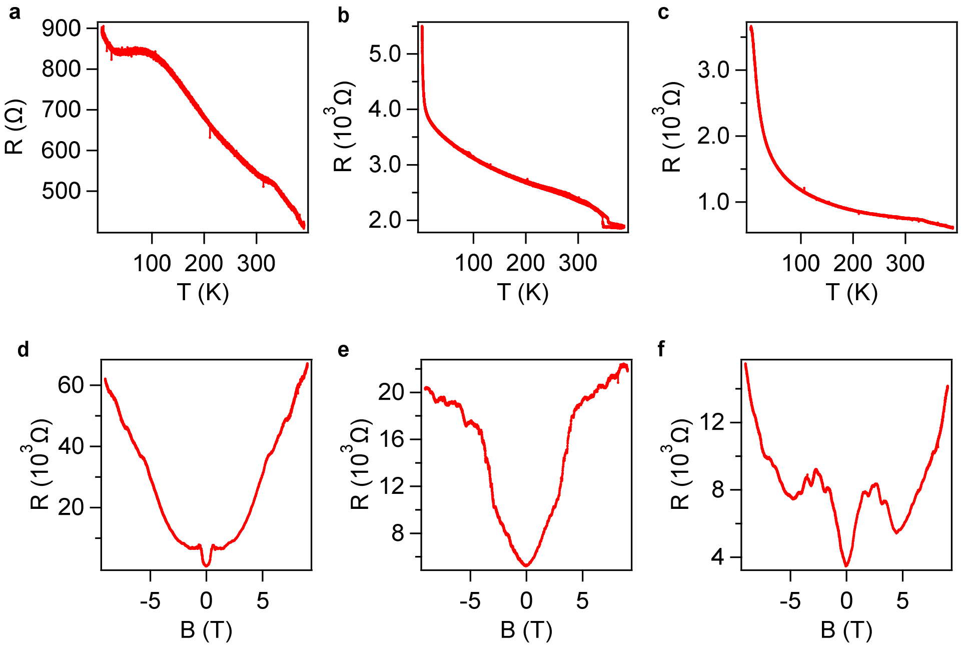

Figure S.47.- Dependence of the resistance as a function of the temperature (a, b, c) and the field at 2 K (d, e, f) for the bottom graphite contact (a, d), the junction (b, e) and the top graphite contact (c, f).

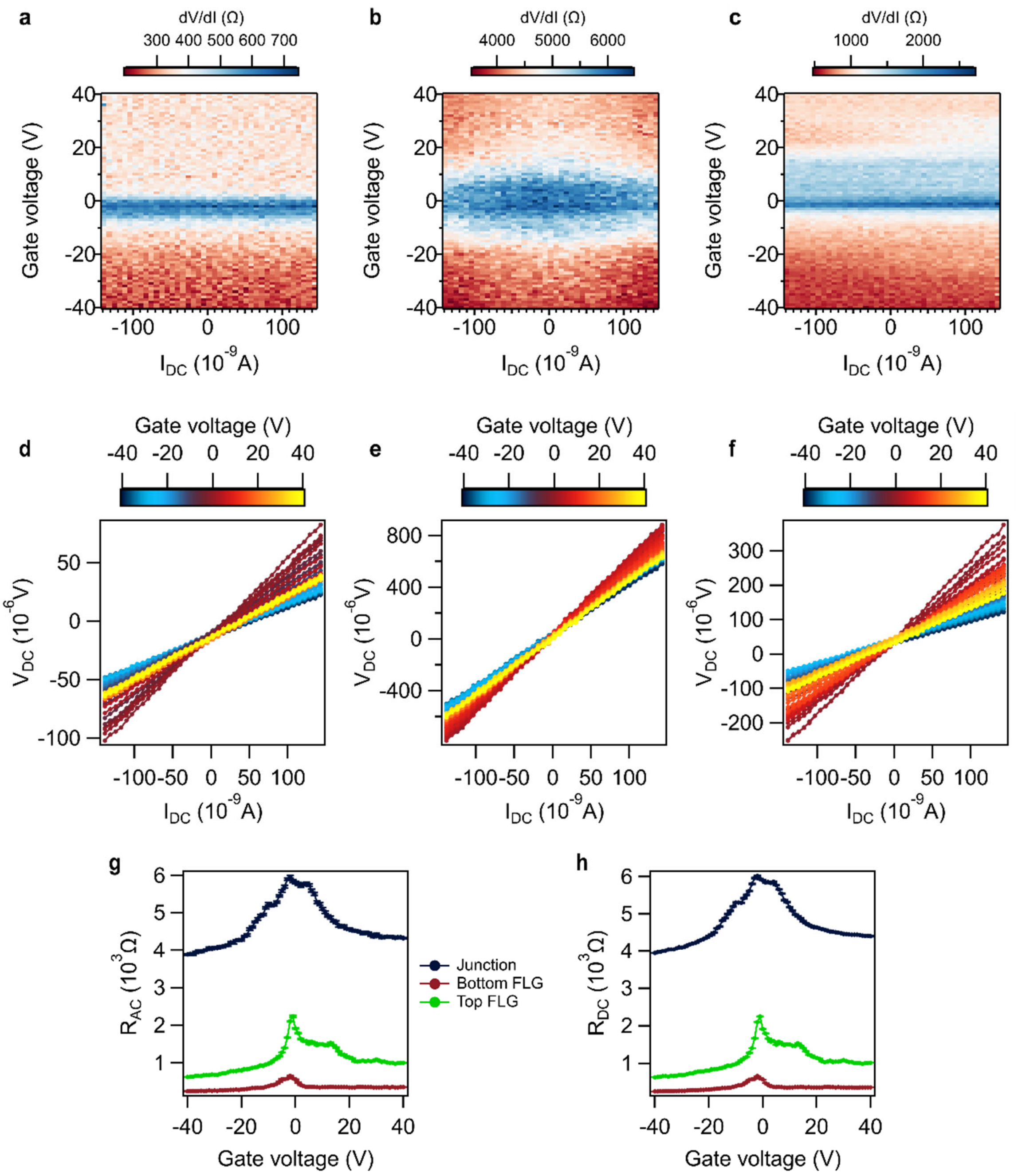

Figure S.48.- (a, b and c) AC Differential resistance of the bottom FLG (a), the junction (b) and the top FLG contact (c) for different gate voltages. (d, e and f) DC IV curves for different gate voltages for the bottom FLG (d), the junction (e) and the top FLG contact (f). (g) Resistance obtained by averaging the AC dV/dI data shown in a, b and c. (h) Resistance obtained by fitting to Ohm´s law the DC IV curves shown in d, e and f.

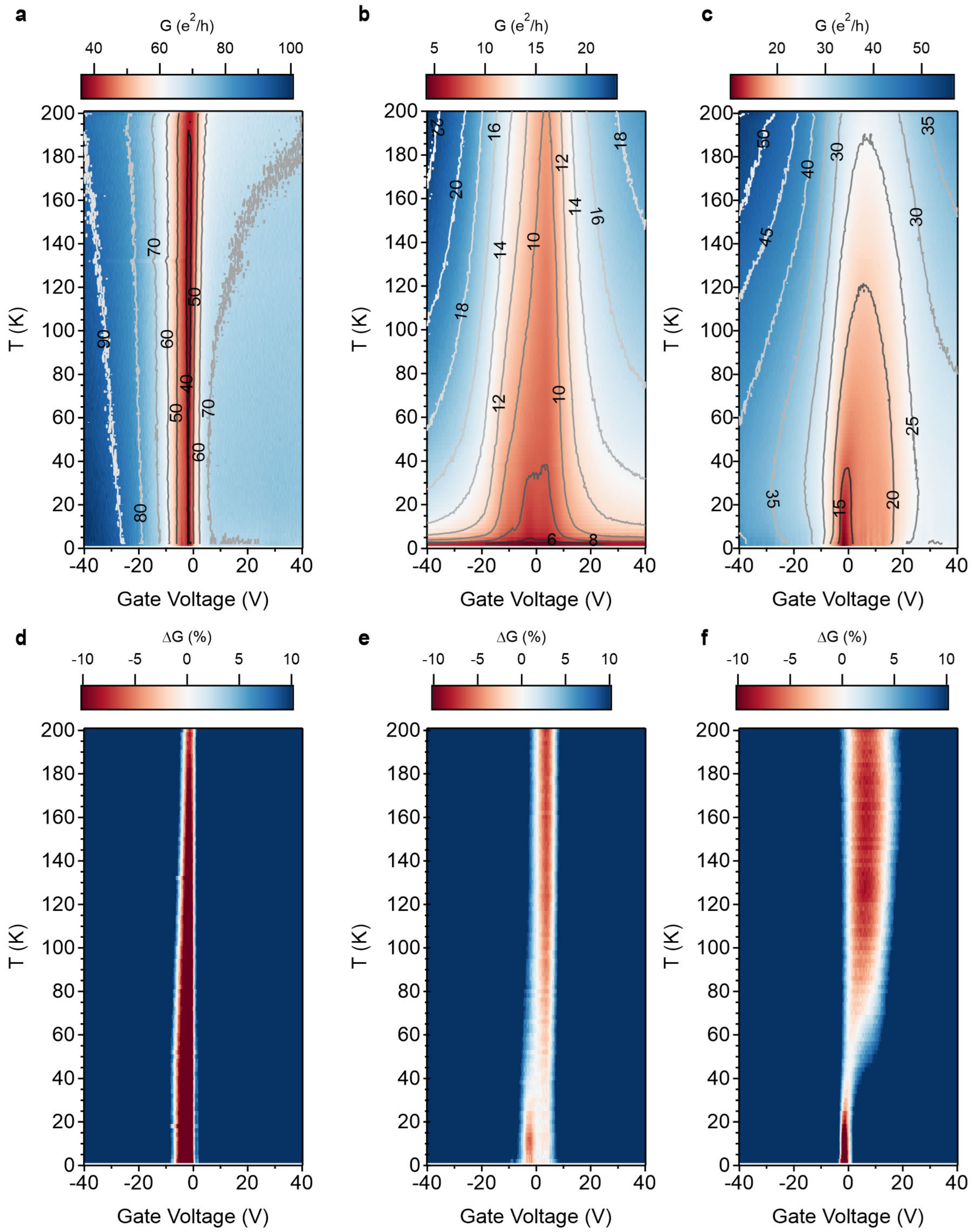

Figure S.49.- Gate and temperature dependence of the conductance (a, b and c) and ΔG (d, e and f) for the bottom FLG (a and d), the junction (b and e) and the top FLG (c and f).

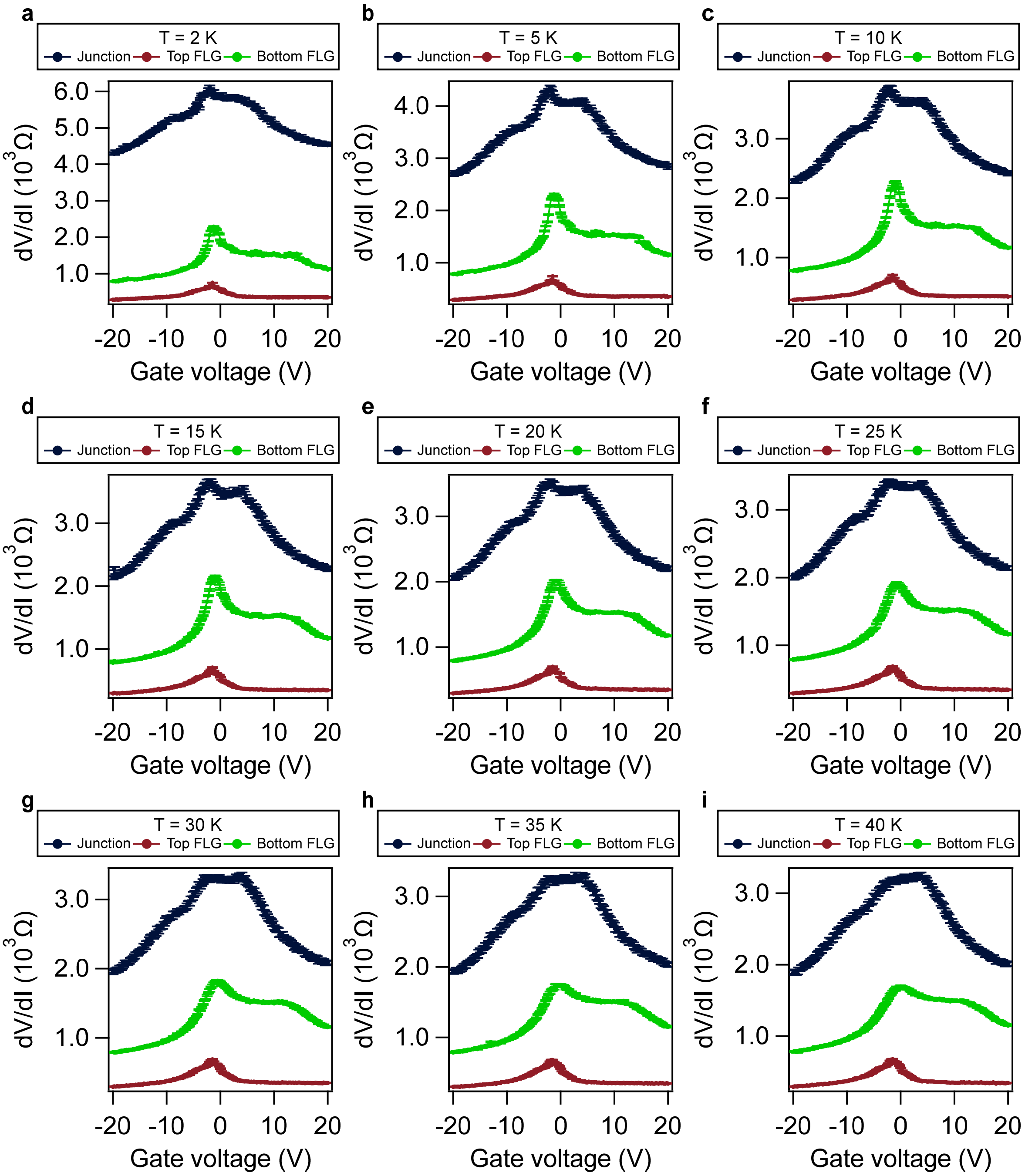

Figure S.50.- AC differential resistance (AC bias of 500 mV and an external resistance of 100 MΩ) for the junction and the top and bottom FLG contacts at different temperatures.

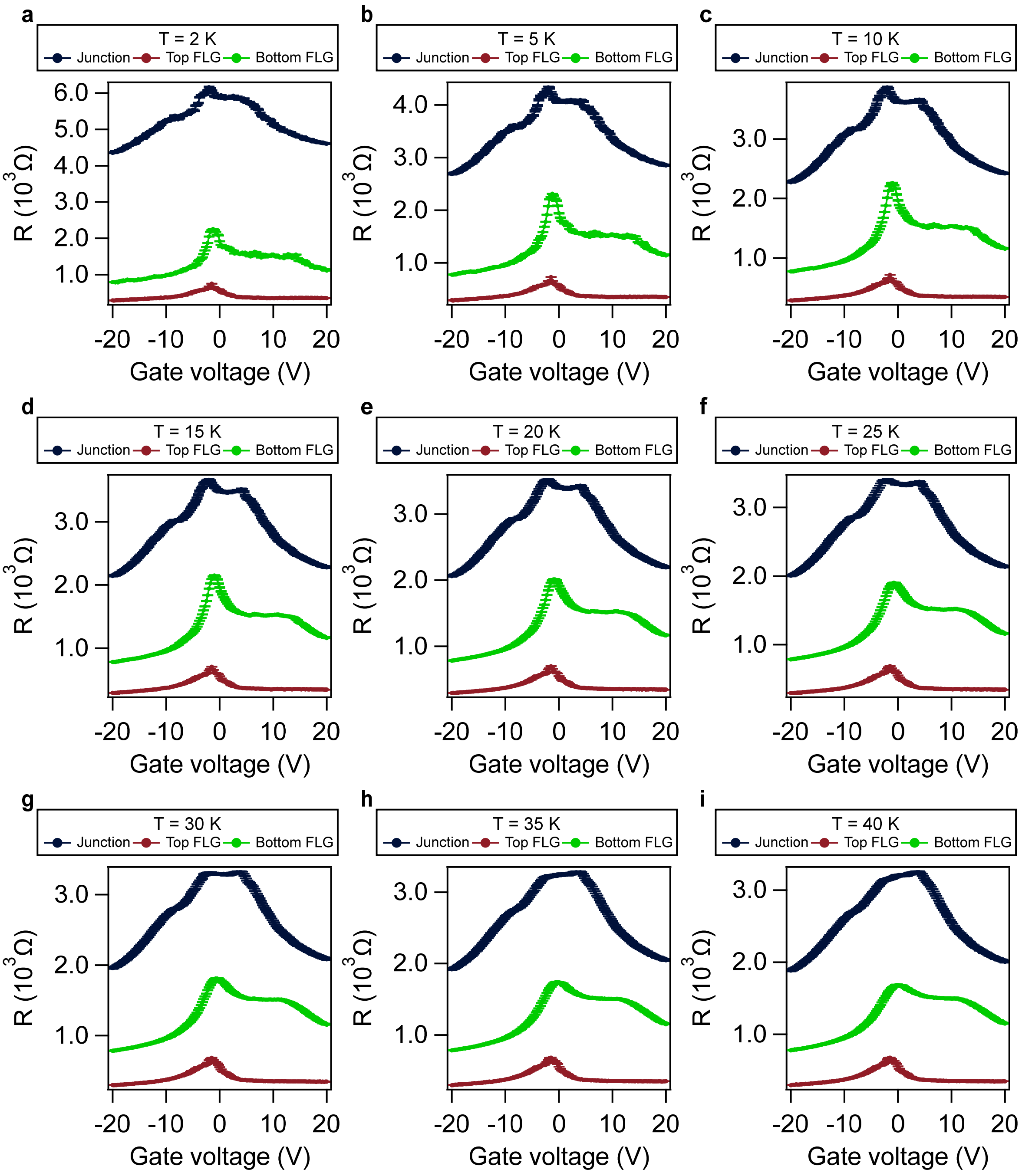

Figure S.51.- Fitted resistance (DC) for the junction and the top and bottom FLG contacts at different temperatures.

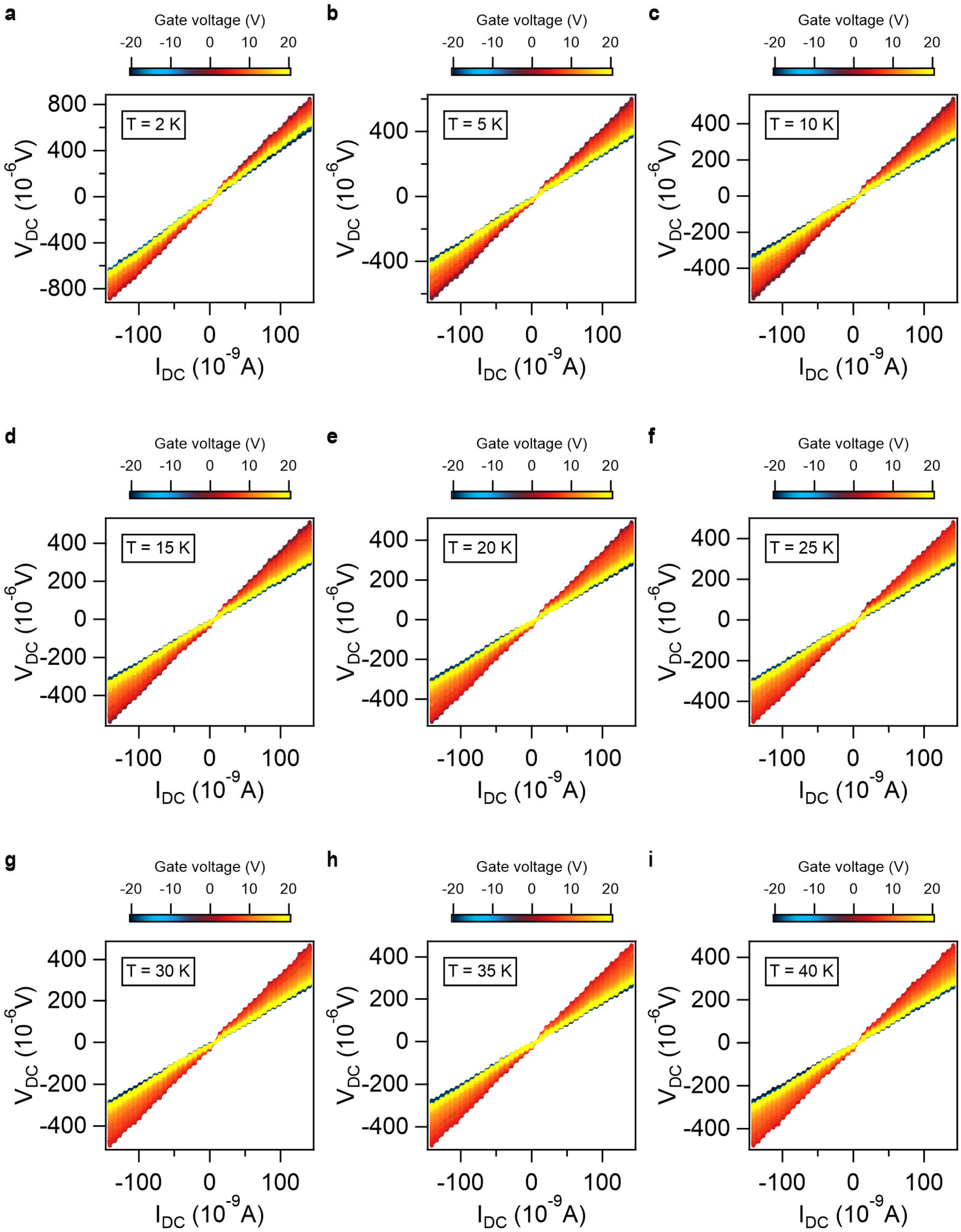

Figure S.52.- Set of DC IV curves at different temperatures for the junction.

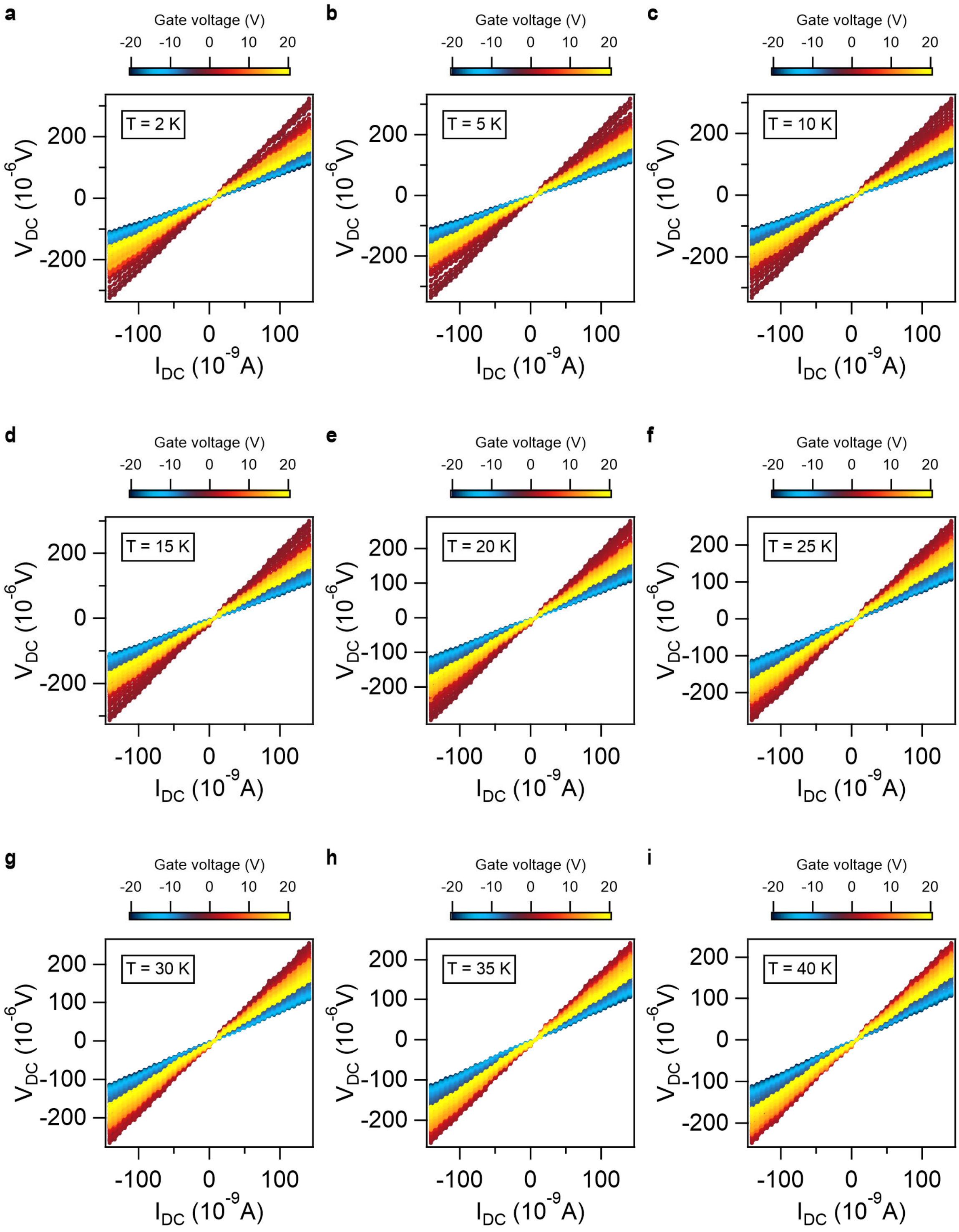

Figure S.53.- Set of DC IV curves at different temperatures for the top FLG.

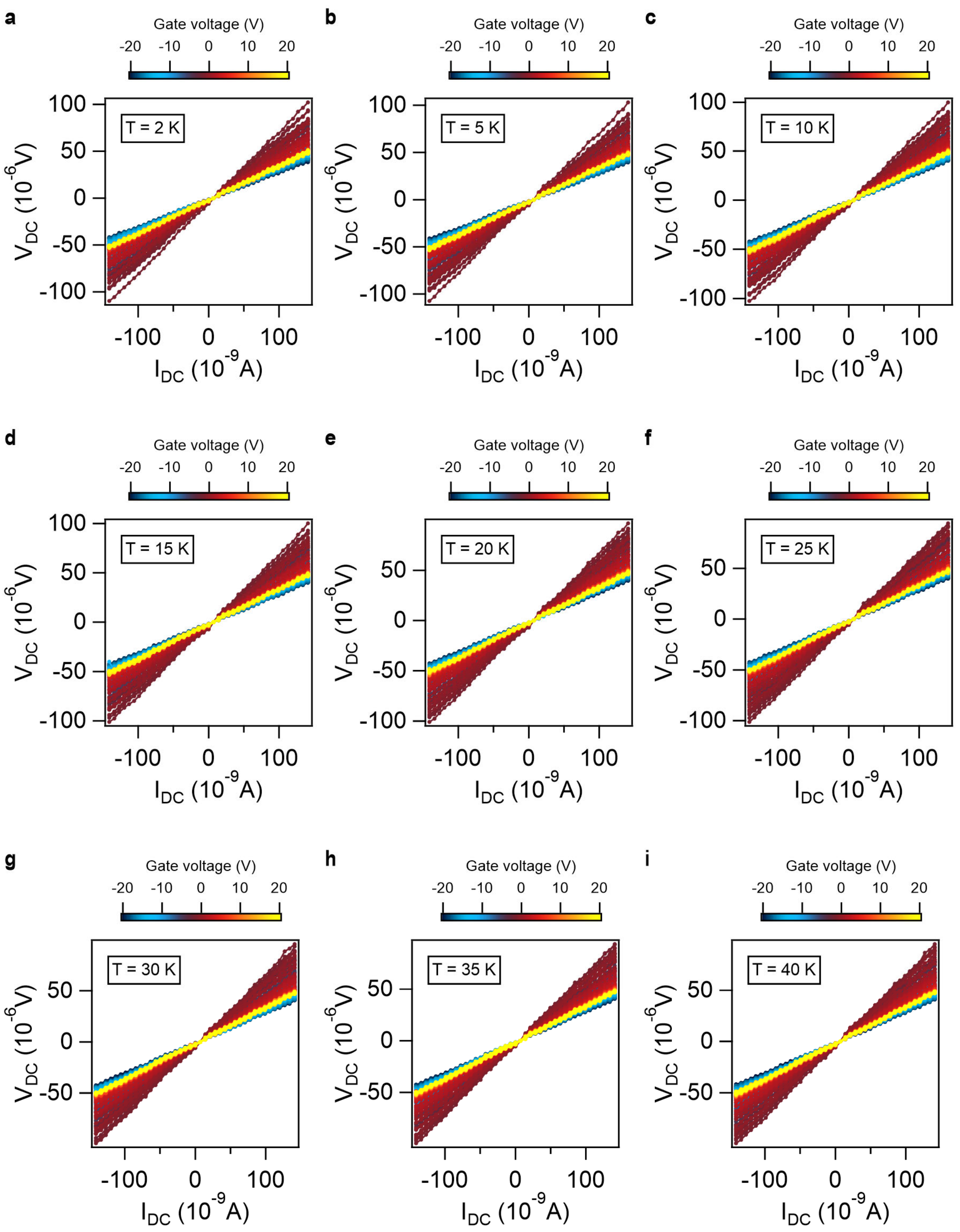

Figure S.54.- Set of DC IV curves at different temperatures for the bottom FLG.

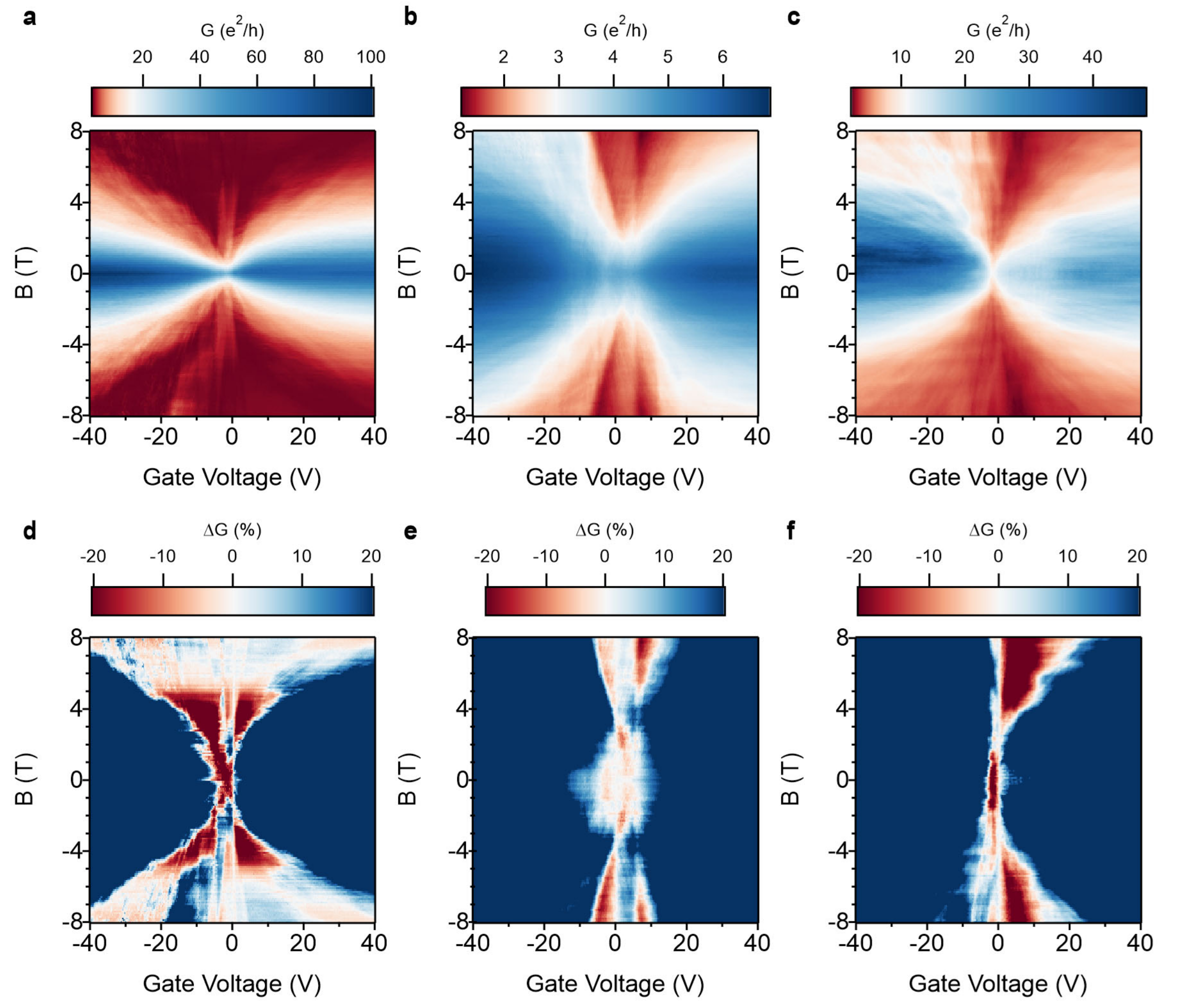

Figure S.55.- Gate and field dependence at 2K of the conductance (a, b and c) and ΔG (d, e and f) for the bottom FLG (a and d), the junction (b and e) and the top FLG (c and f).

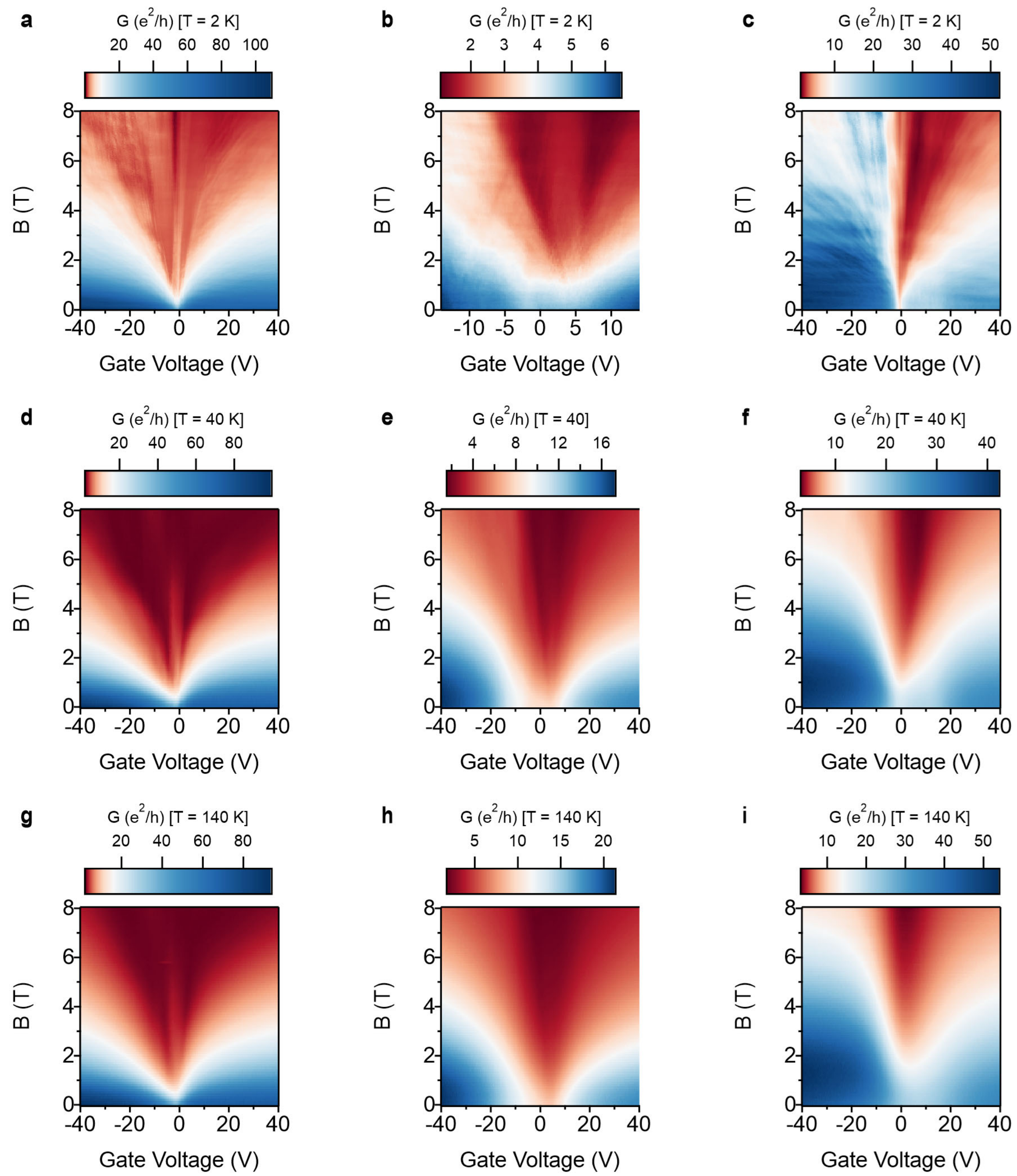

Figure S.56.- Gate and field dependence of the conductance at 2 K (a, b and c), 40 K (d, e and f) and 140 K (g, h and i) for the bottom FLG contact (a, d and g), junction (b, e and h) and top FLG contact (c, f and i).

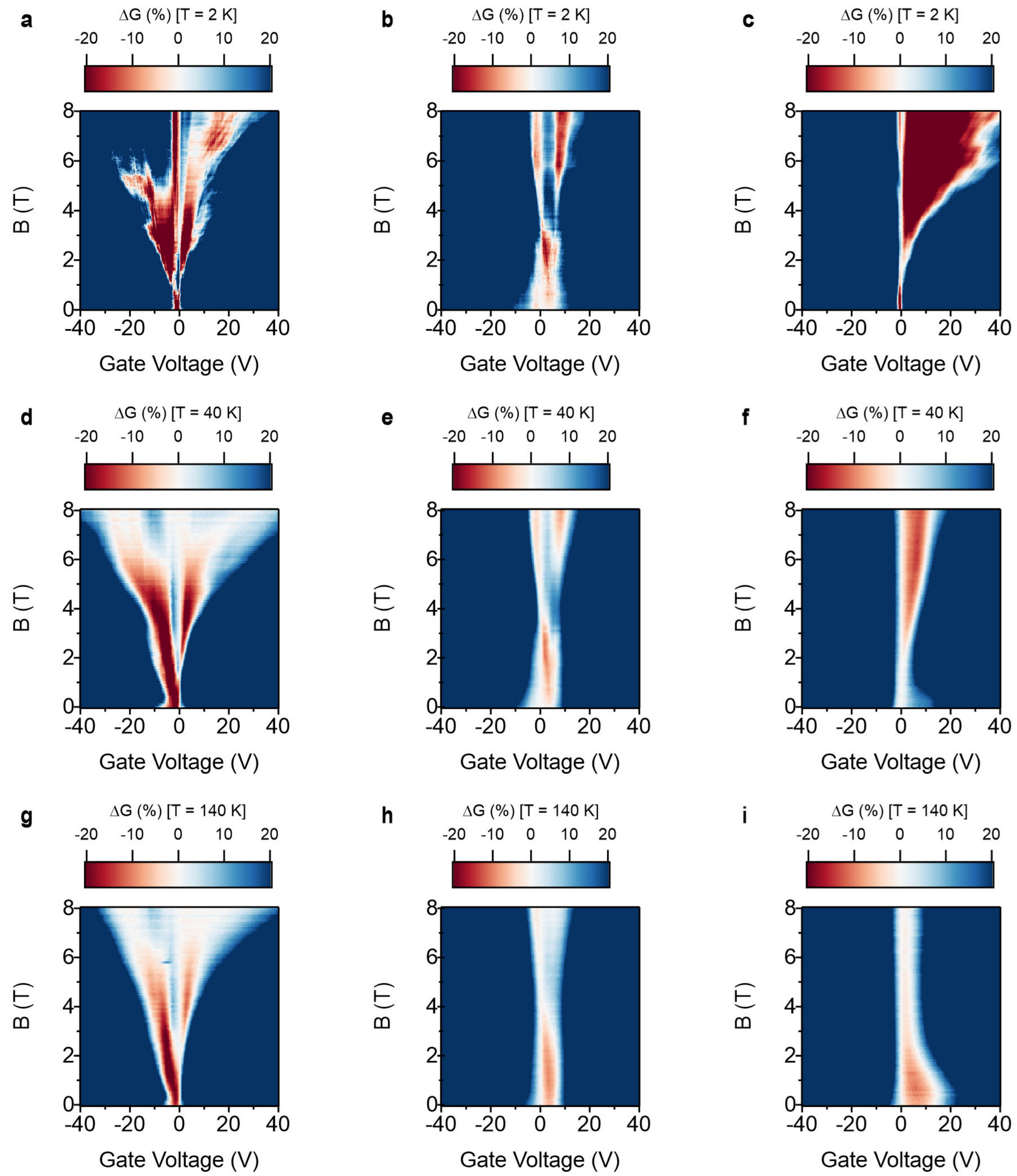

Figure S.57.- Gate and field dependence of ΔG at 2 K (a, b and c), 40 K (d, e and f) and 140 K (g, h and i) for the bottom FLG contact (a, d and g), junction (b, e and h) and top FLG contact (c, f and i).

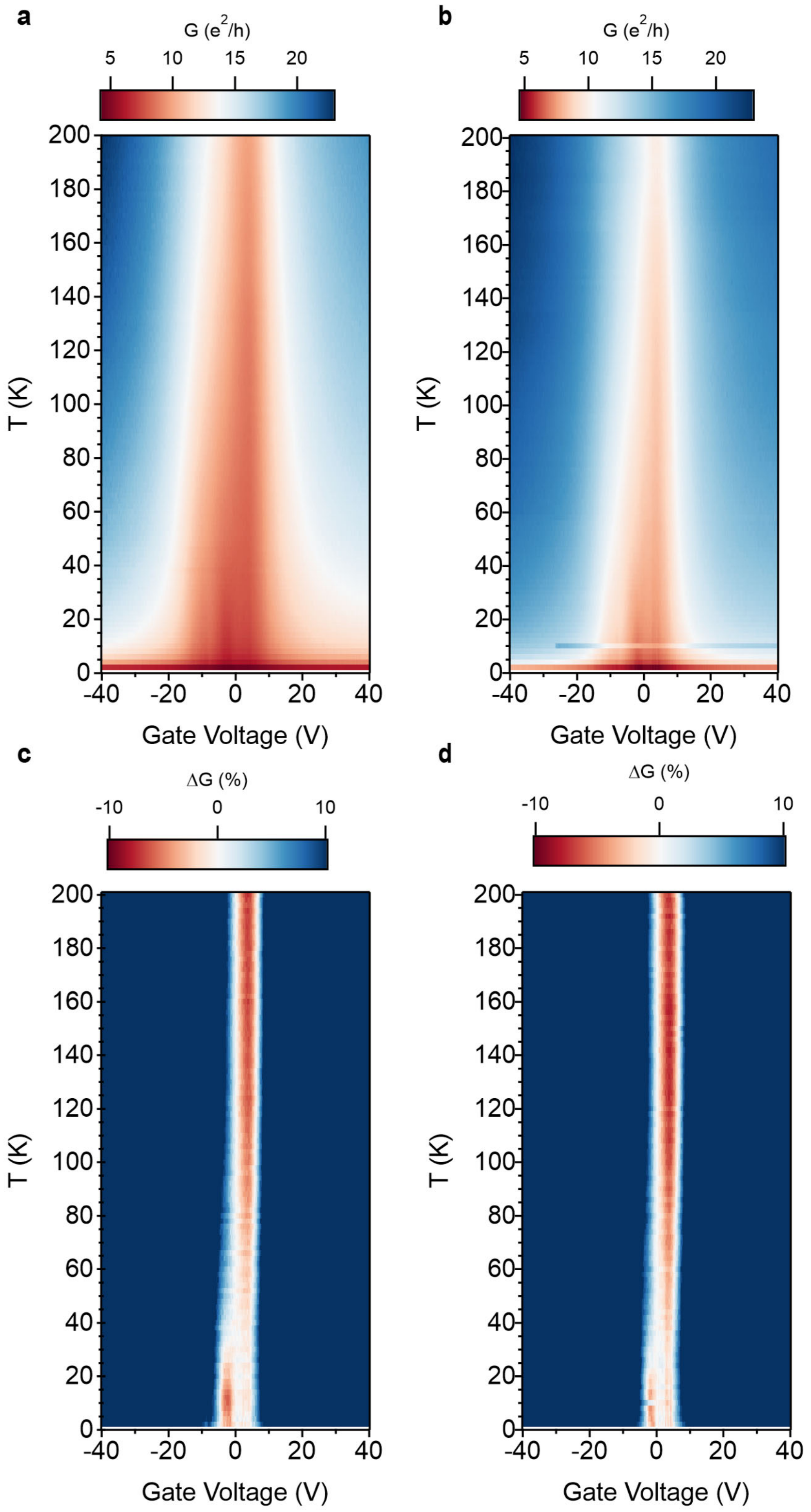

Figure S.58.- Gate and temperature dependence of the conductance (a and b) and ΔG (c and d) for the junction while cooling down (from 200 K to 2 K; a and c) and warming up (from 2 K to 200 K; b and d).

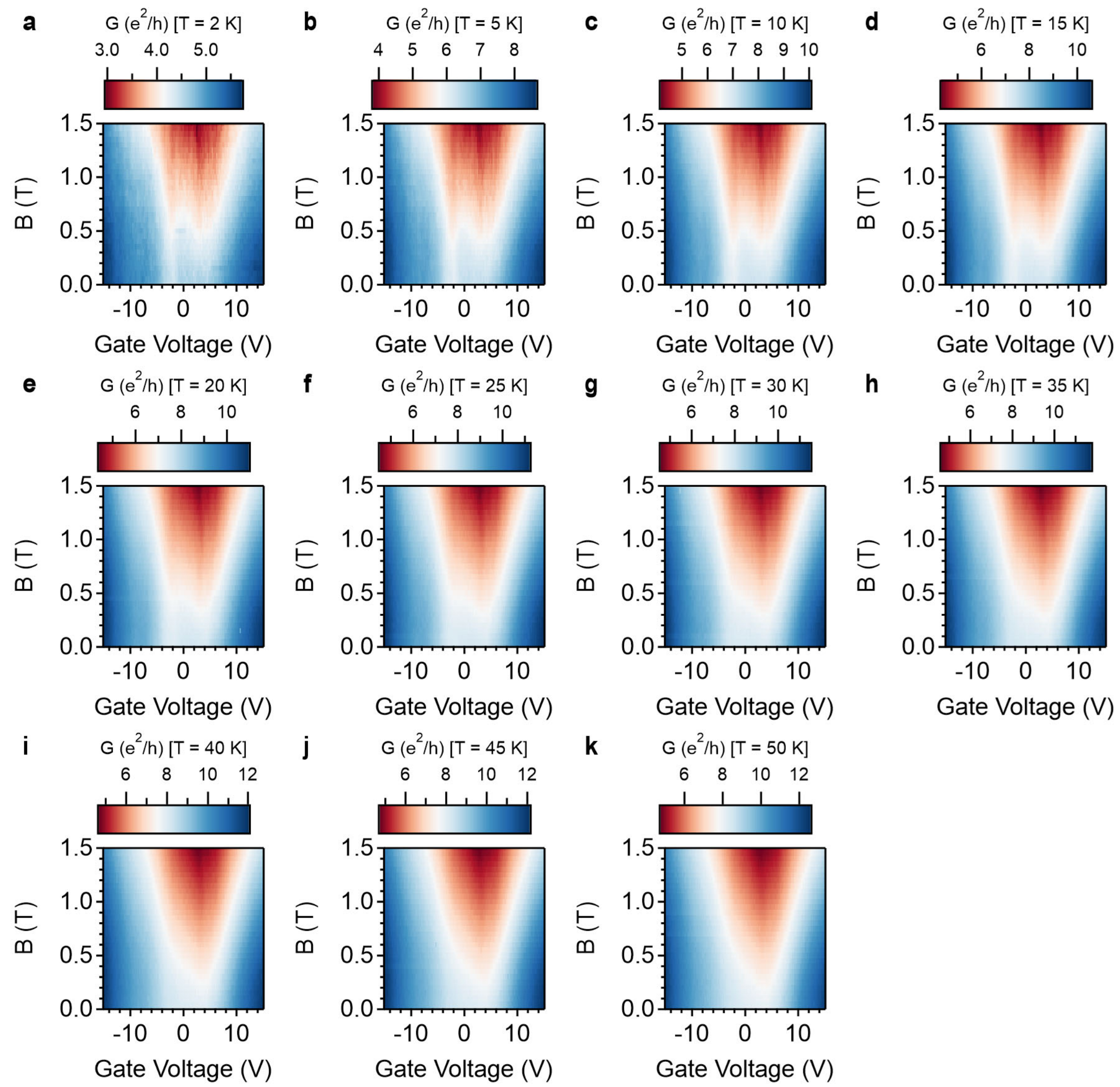

Figure S.59.- Gate and field dependence of the junction conductance at different temperatures.

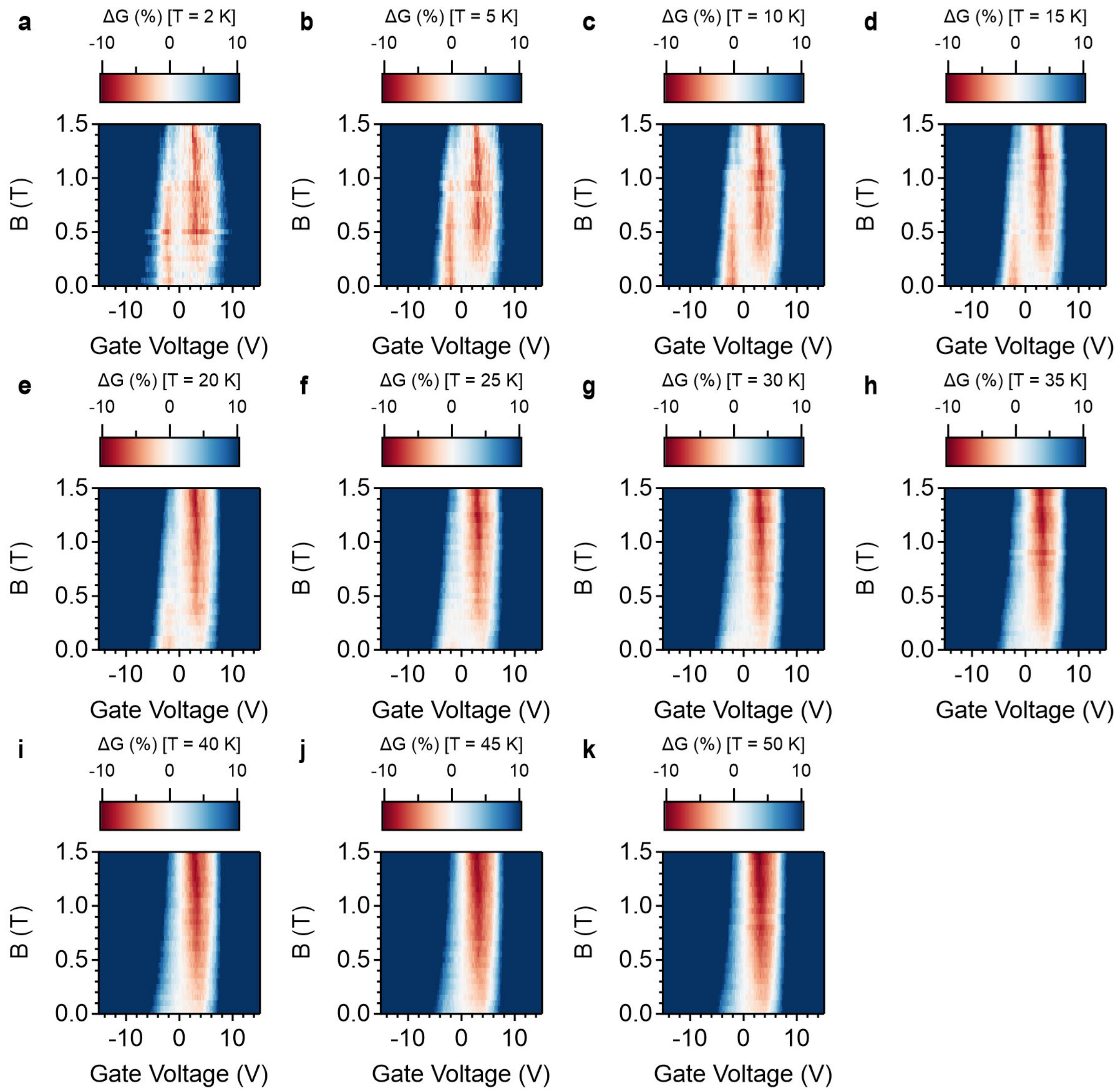

Figure S.60.- Gate and field dependence of the junction ΔG at different temperatures.

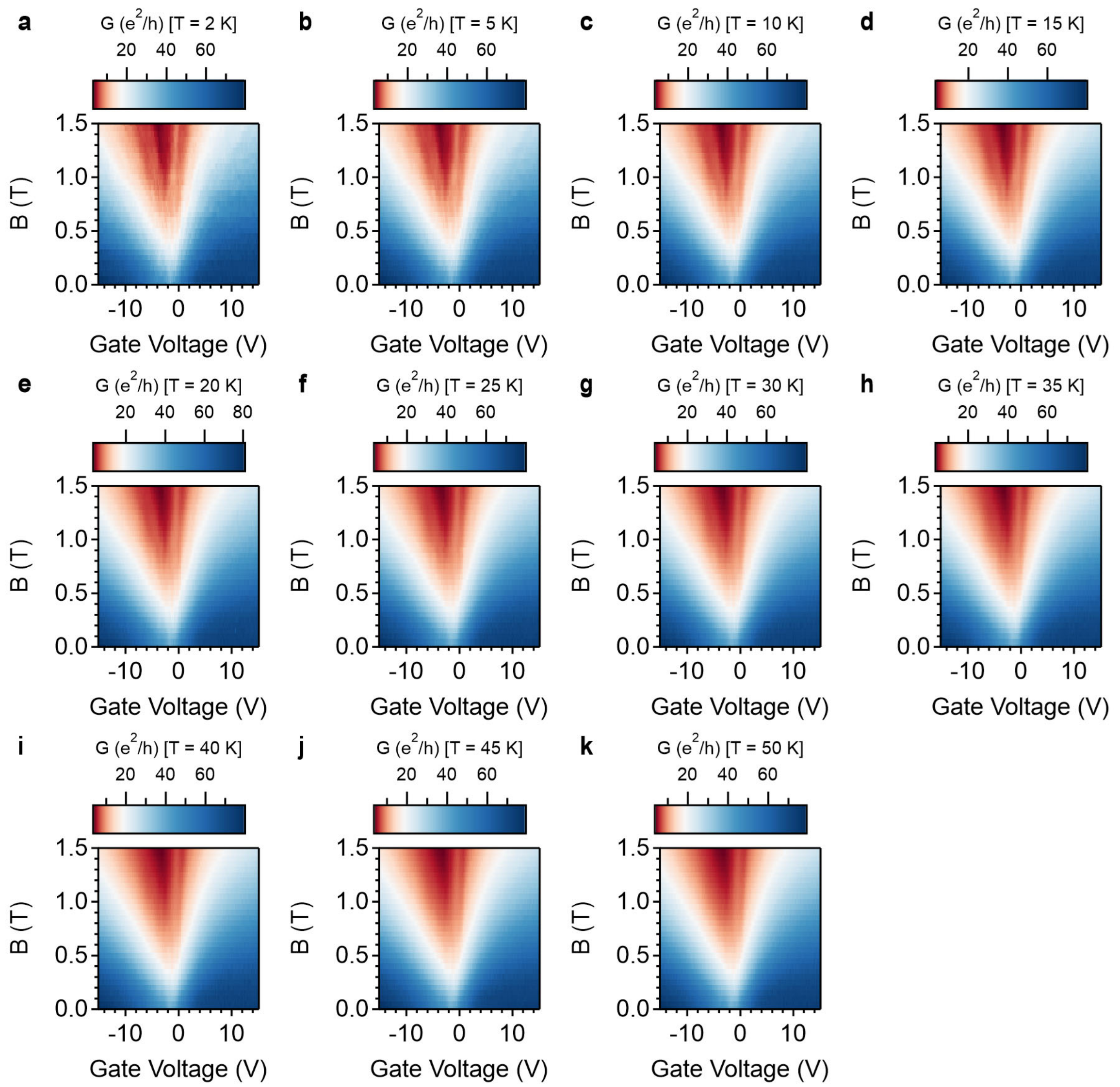

Figure S.61.- Gate and field dependence of the bottom contact conductance at different temperatures.

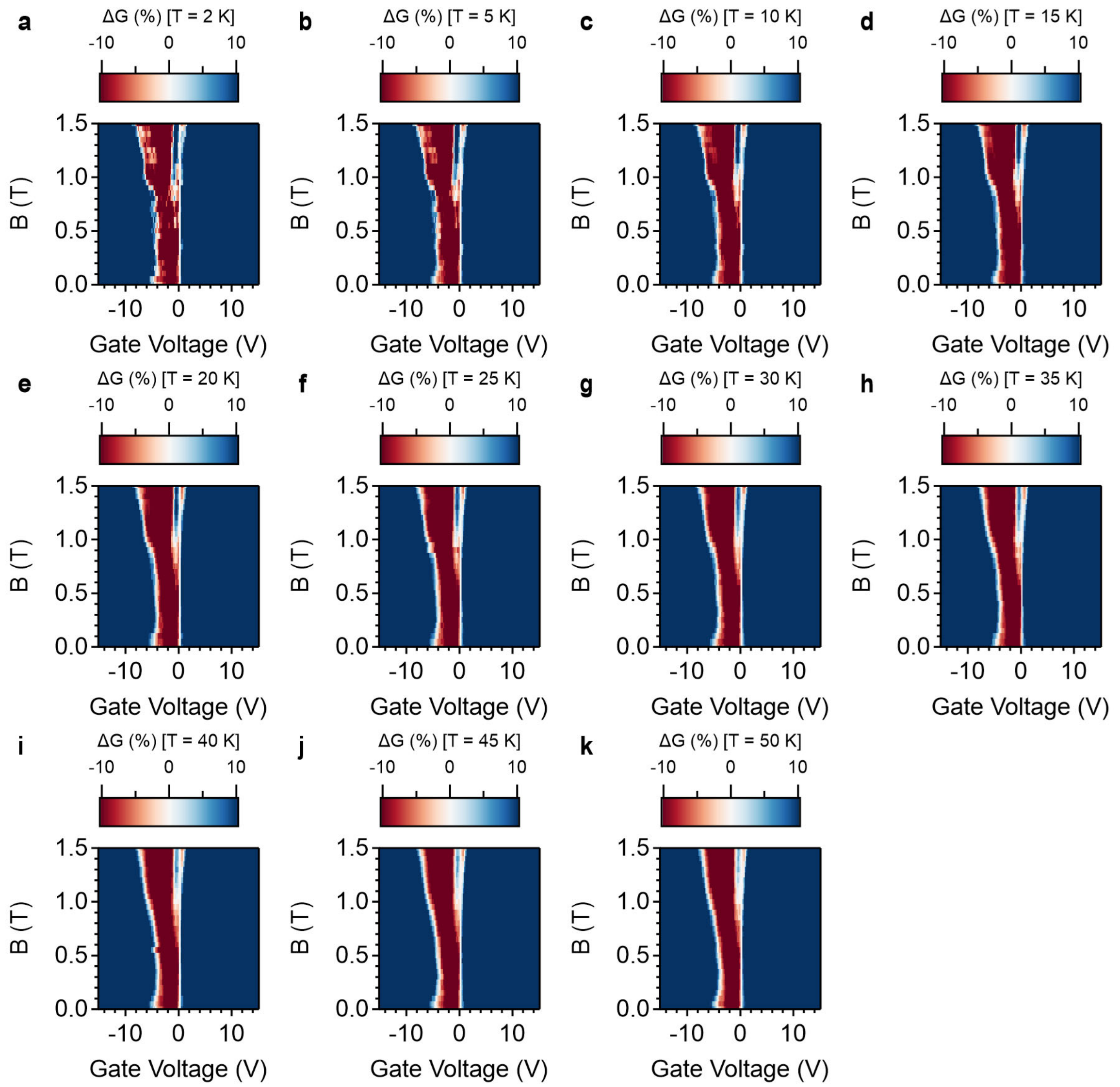

Figure S.62.- Gate and field dependence of the bottom contact ΔG at different temperatures.

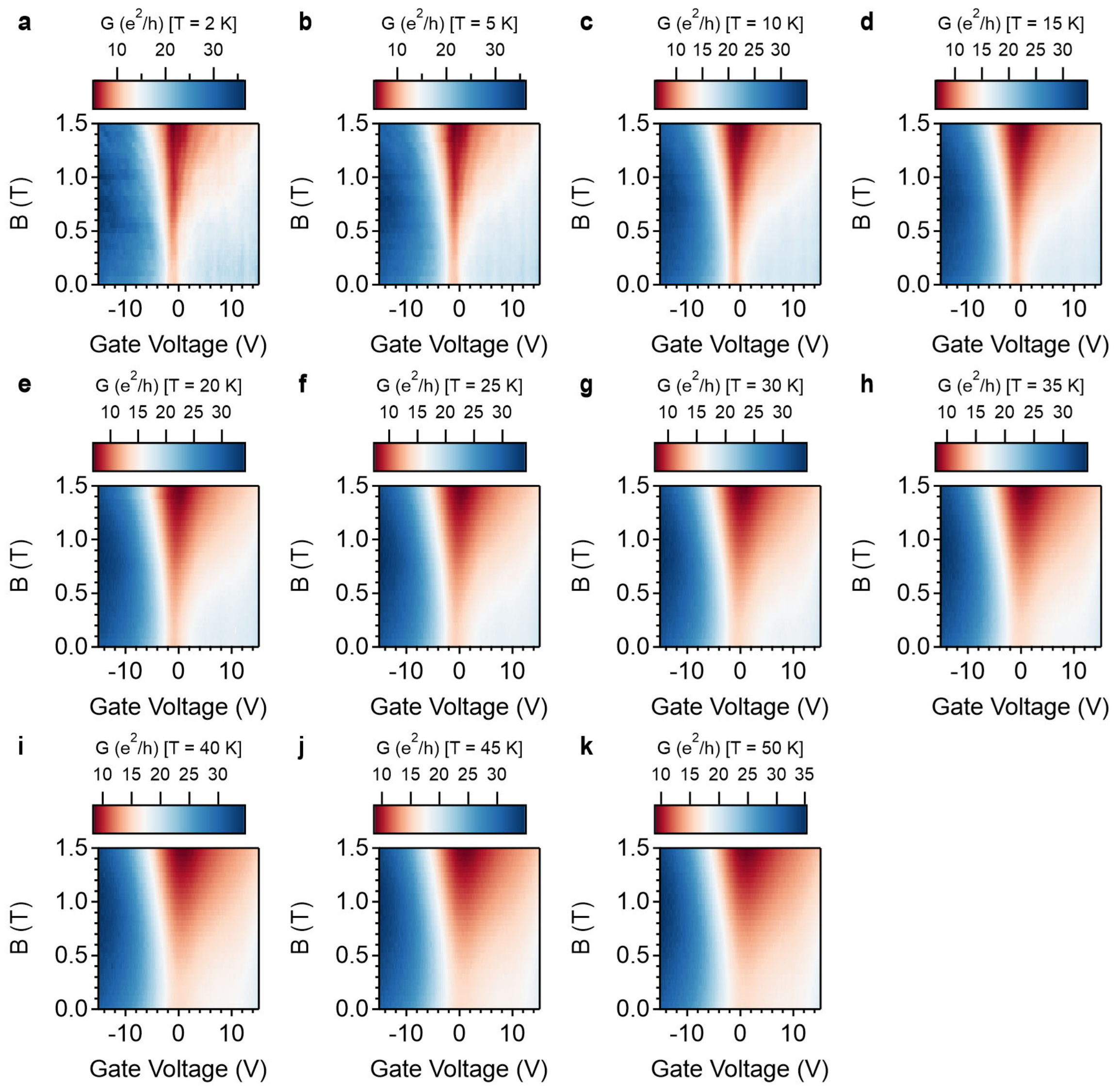

Figure S.63.- Gate and field dependence of the top contact conductance at different temperatures.

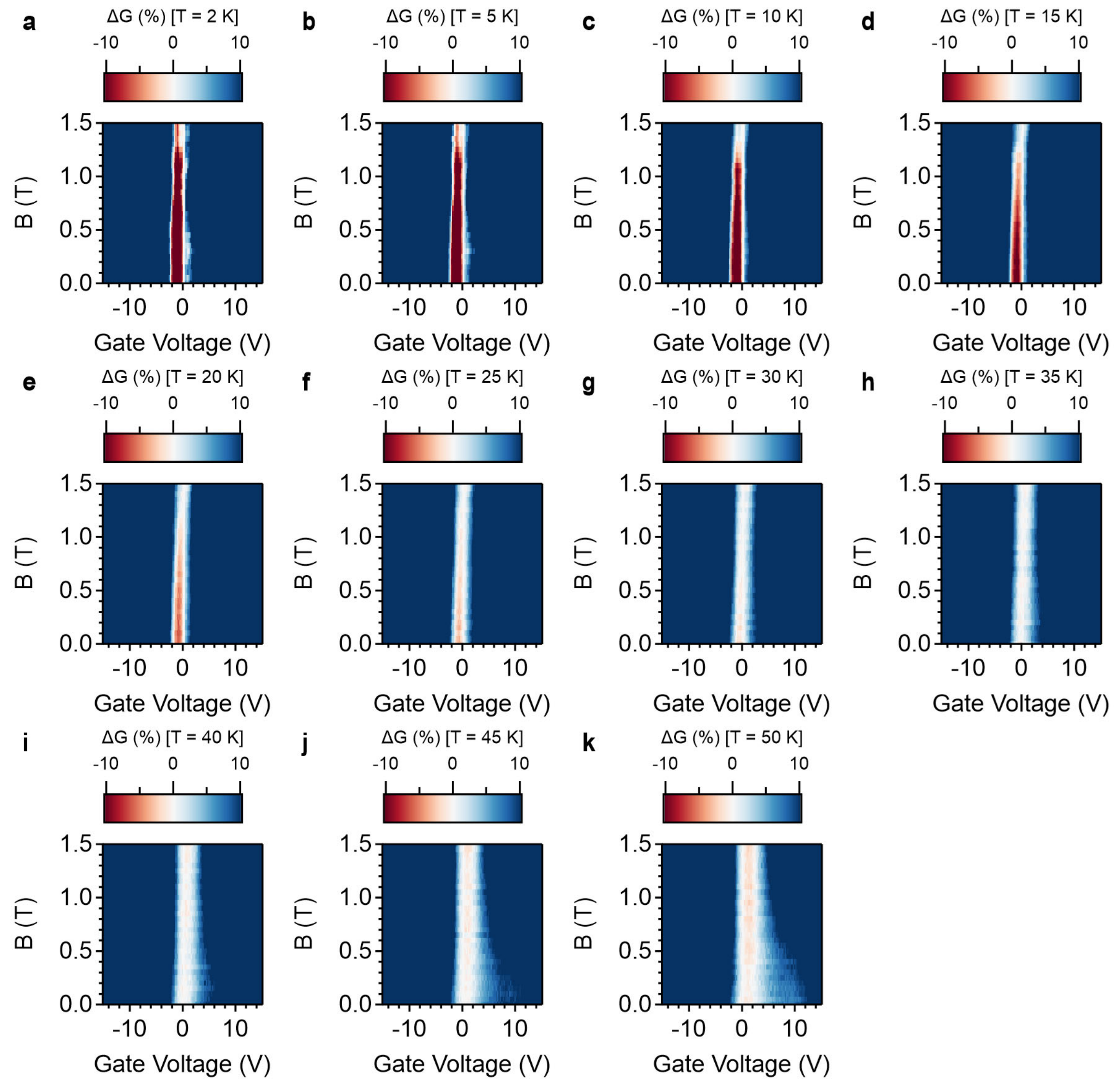

Figure S.64.- Gate and field dependence of the top contact ΔG at different temperatures.

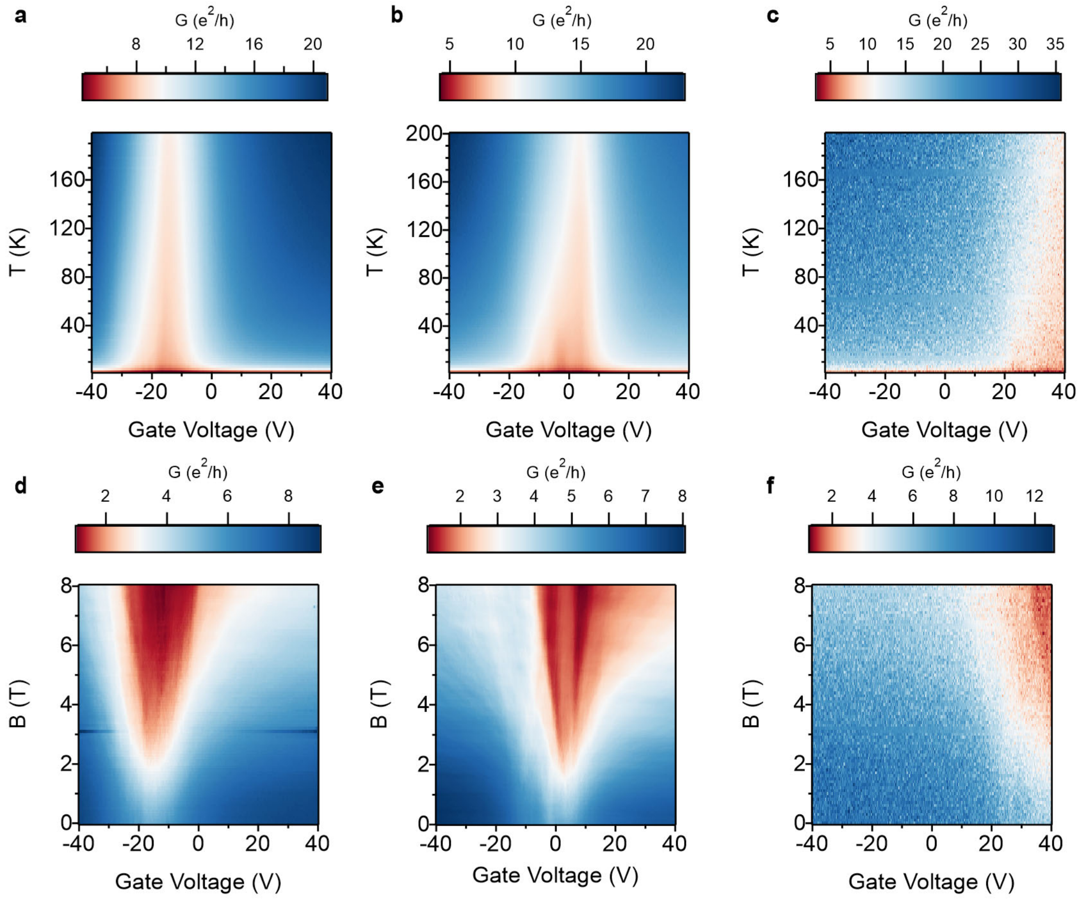

Figure S.65.- Gate and temperature dependence of the conductance of the junction while cooling down with an applied gate voltage of – 35 V (a), 0 V (b) and + 35 V (c). Gate and field dependence at 2 K of the conductance of the junction while cooling down with an applied gate voltage of – 35 V (d), 0 V (e) and + 35 V (f).

4.2. Device B.

Device

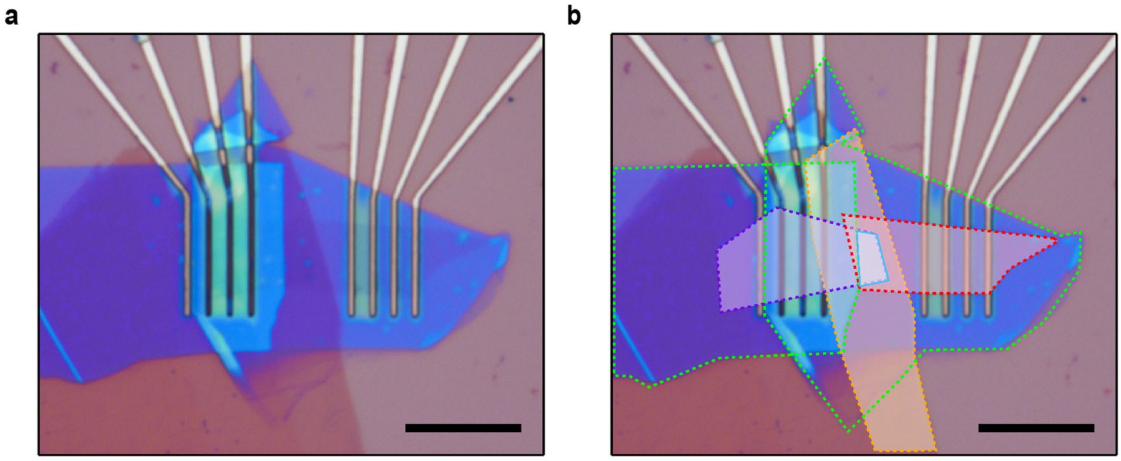

Figure S.66.- Optical picture of the device. For simplicity, the top graphite contact has been highlighted in red, the bottom graphite contact in purple and the 1T-TaS_2 barrier in orange. h-BN is marked in green. The junction (blue area) is the overlapped area formed by the three different materials. Scale bar: 10 µm.

Transport measurements

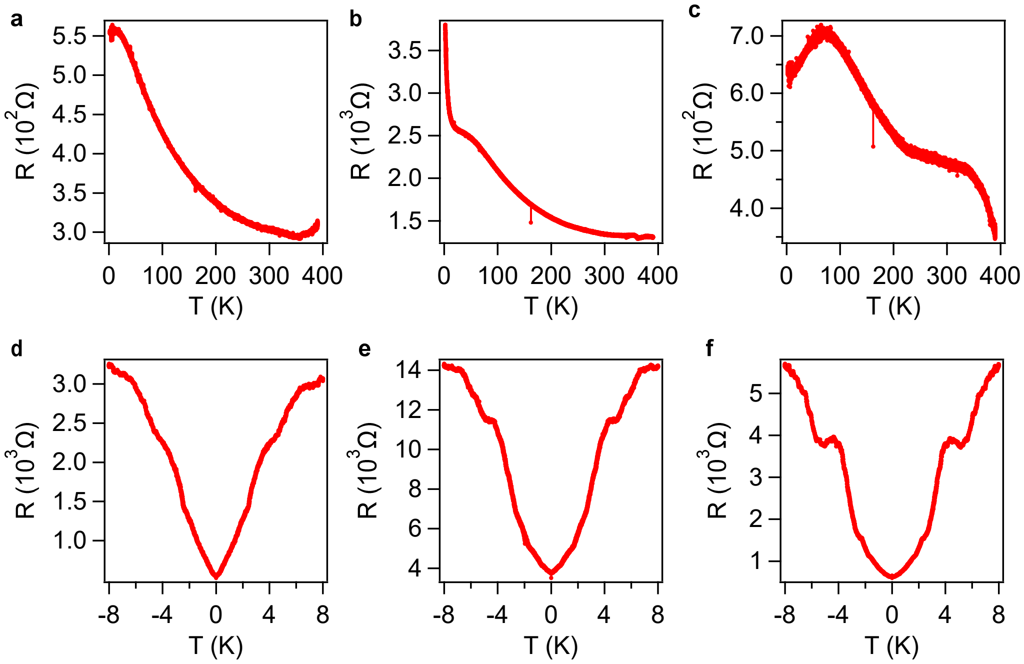

Figure S.67.- Dependence of the resistance as a function of the temperature (a,b,c) and the field (d,e,f) for the bottom graphite contact (a,d), the junction (b,e) and the top graphite contact (c,f).

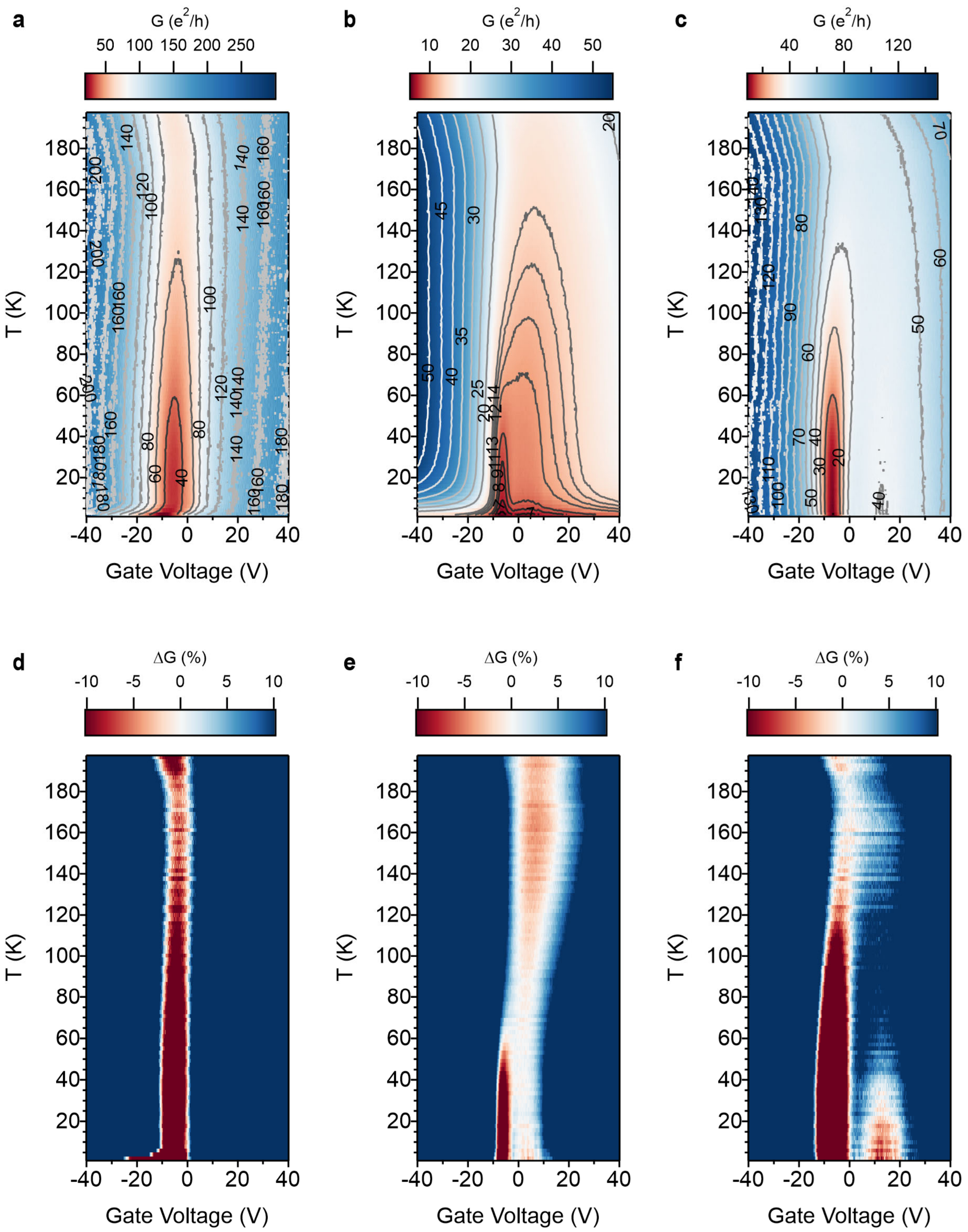

Figure S.68.- Gate and temperature dependence of the conductance (a, b and c) and ΔG (d, e and f) for the bottom FLG (a and d), the junction (b and e) and the top FLG (c and f).

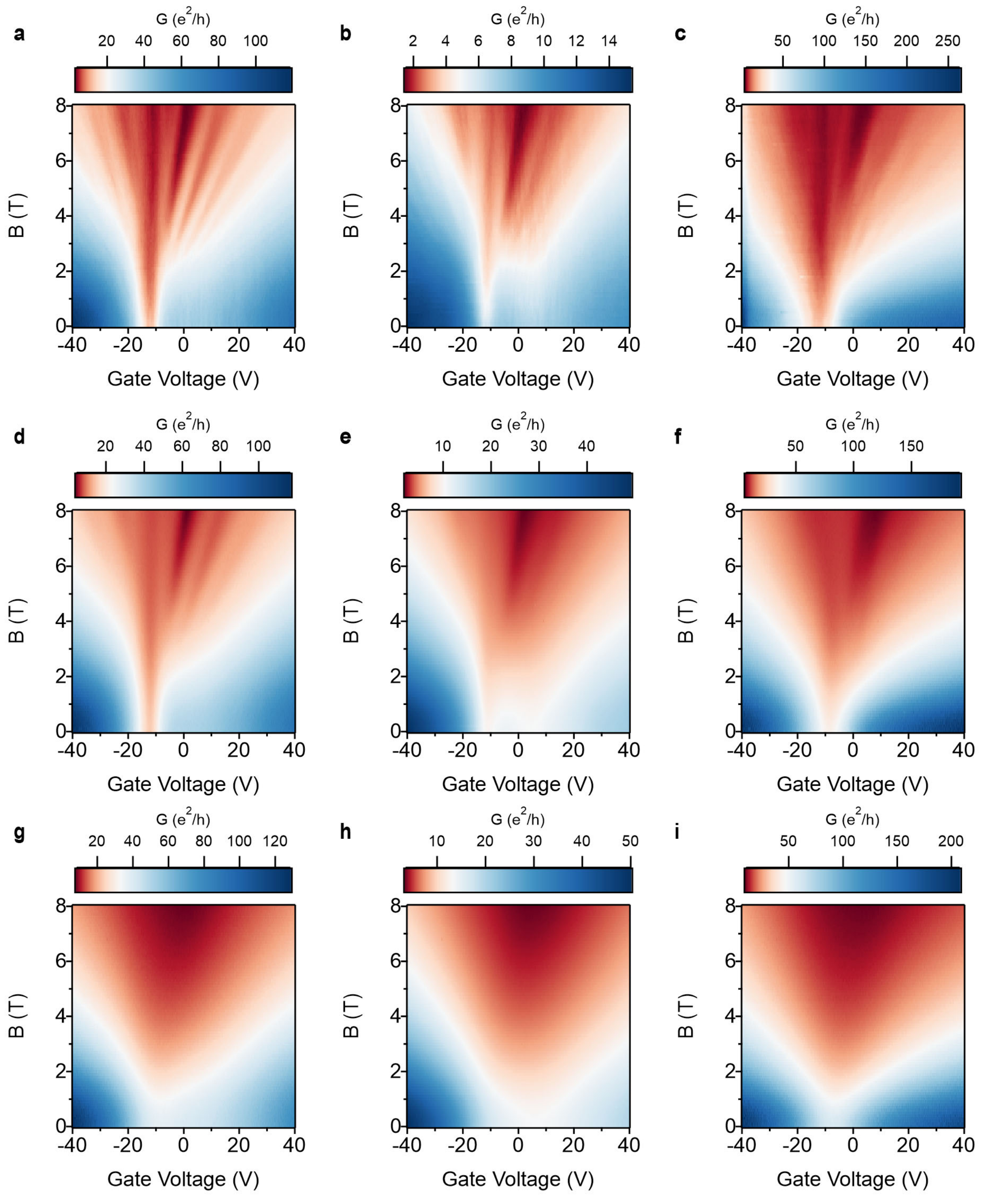

Figure S.69.- Conductance of the bottom FLG contact (a,d,g), the junction (b,e,h) and the top FLG contact (c,f,i) as a function of the gate voltage and external magnetic field at 2K (a, b and c), 40 K (d, e and f) and 140 K (g, h and i).

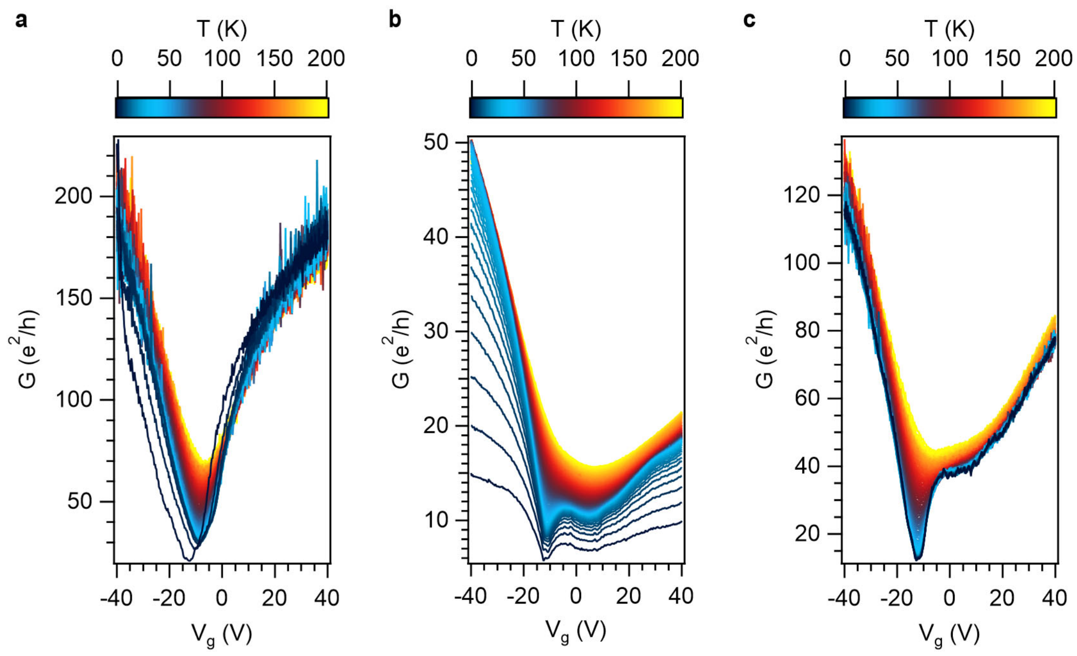

Figure S.70.- Gate dependence of the conductance at different temperatures for the bottom FLG (a), the junction (b) and the top FLG (c).

4.3. Device C.

Device

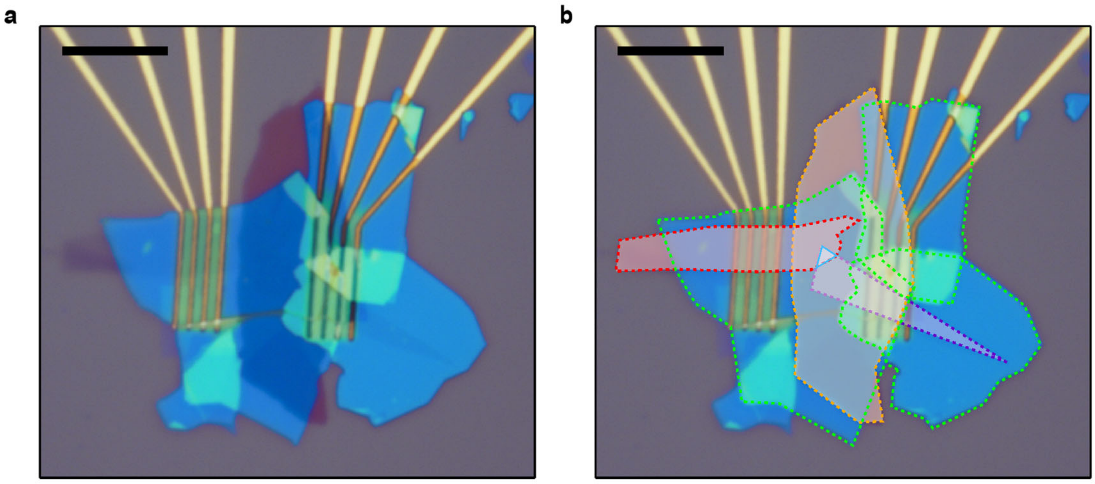

Figure S.71.- Optical picture of the device. For simplicity, the top graphite contact has been highlighted in red, the bottom graphite contact in purple and the 1T-TaS_2 barrier in orange. h-BN is marked in green. The junction (blue area) is the overlapped area formed by the three different materials. Scale bar: 10 μm.

Transport measurements

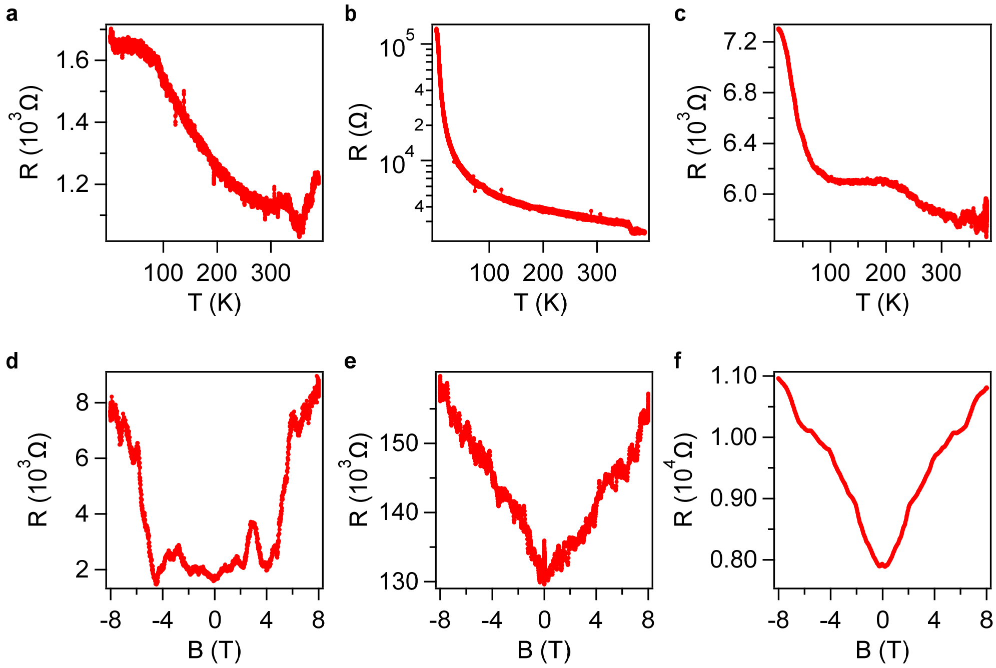

Figure S.72.- Dependence of the resistance as a function of the temperature (a,b,c) and the field at 2 K (d,e,f) for the bottom graphite contact (a,d), the junction (b,e) and the top graphite contact (c,f).

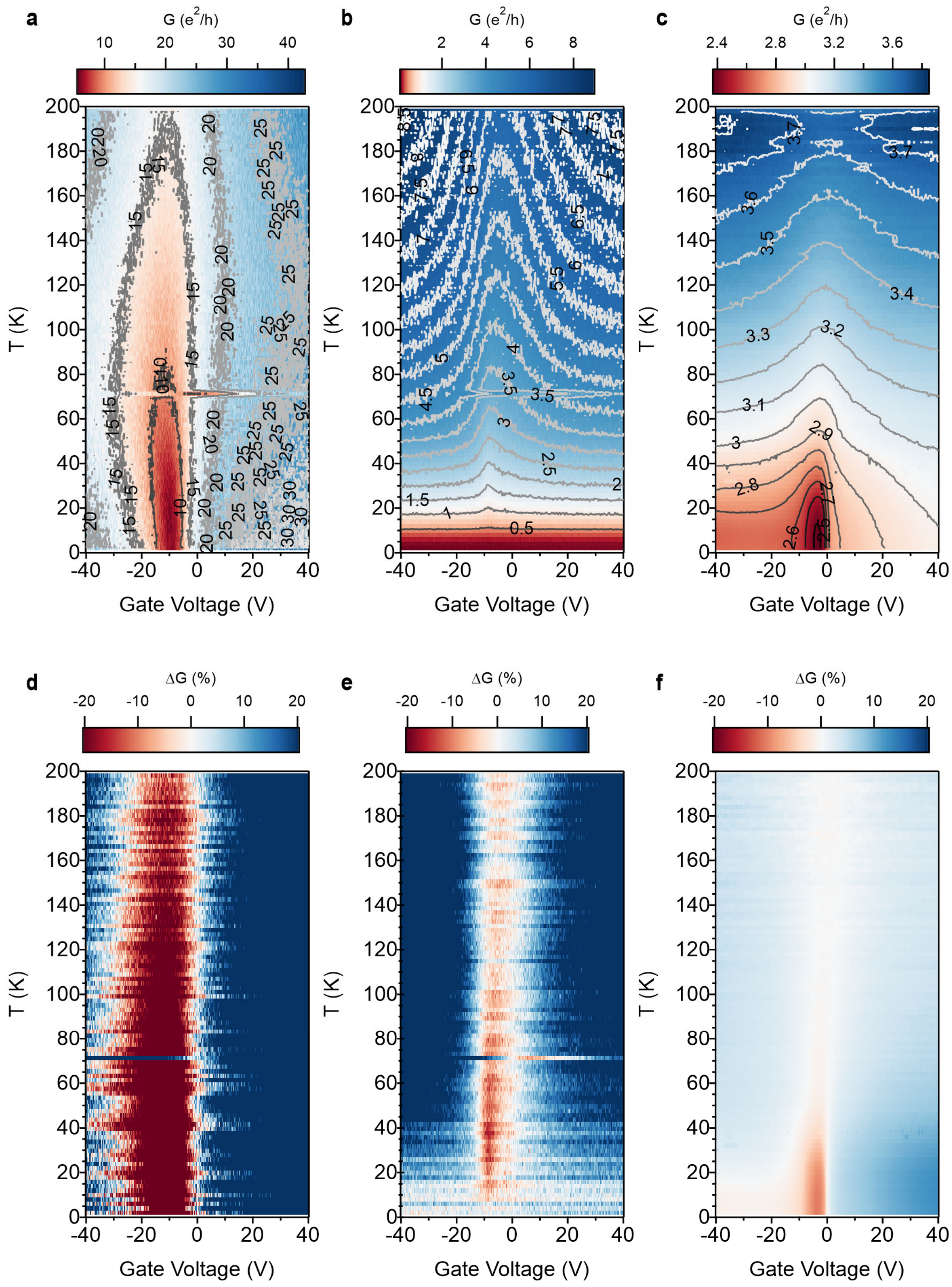

Figure S.73.- Gate and temperature dependence of the conductance (a, b and c) and ΔG (d, e and f) for the bottom FLG (a and d), the junction (b and e) and the top FLG (c and f).

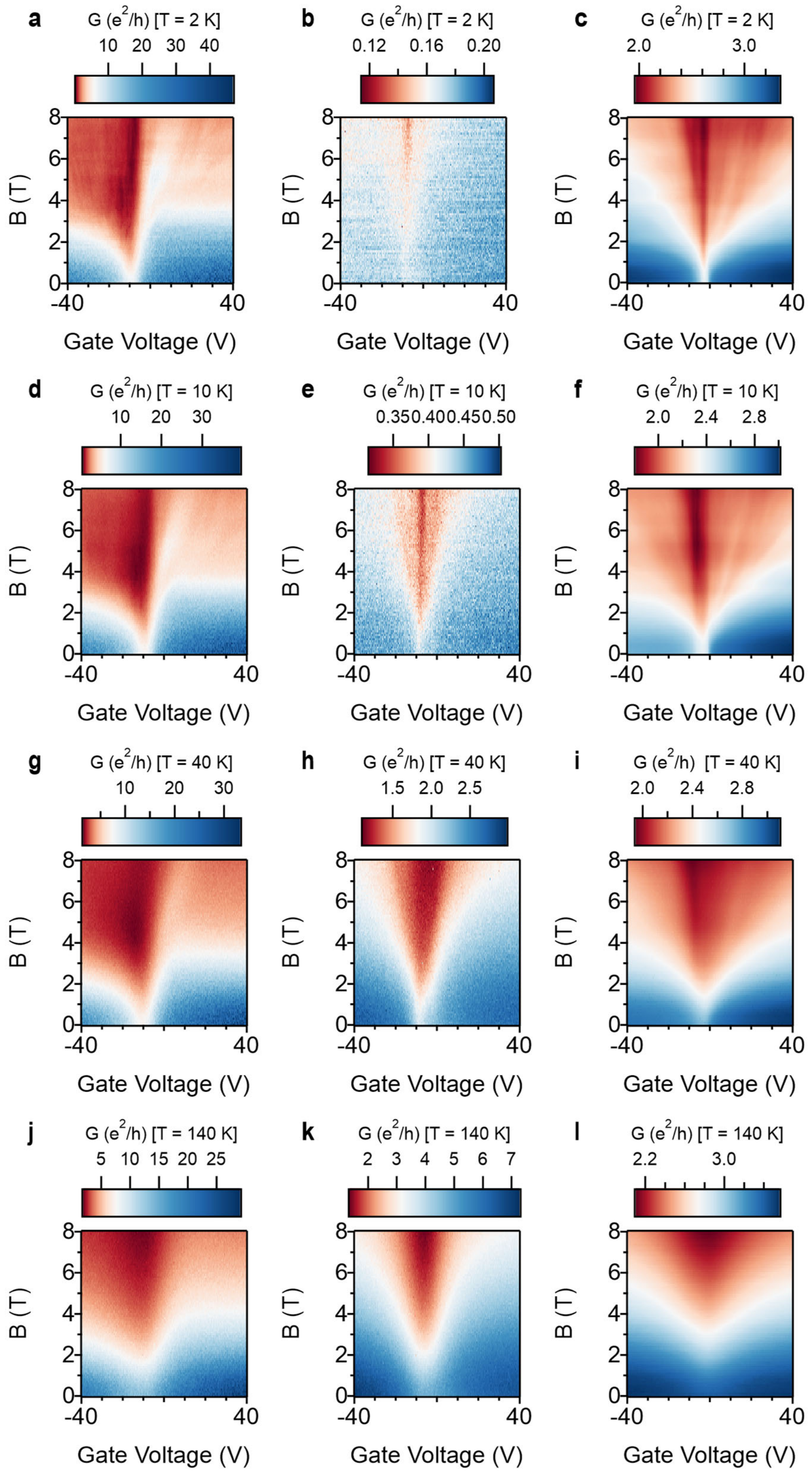

Figure S.74.- Gate and field dependence of the conductance at 2 K (a, b and c), 10 K (d, e and f), 40 K (g, h and i) and 140 K (j, k and l) for the bottom FLG contact (a, d, g and j), junction (b, e, h and k) and top FLG contact (c, f, i and l).

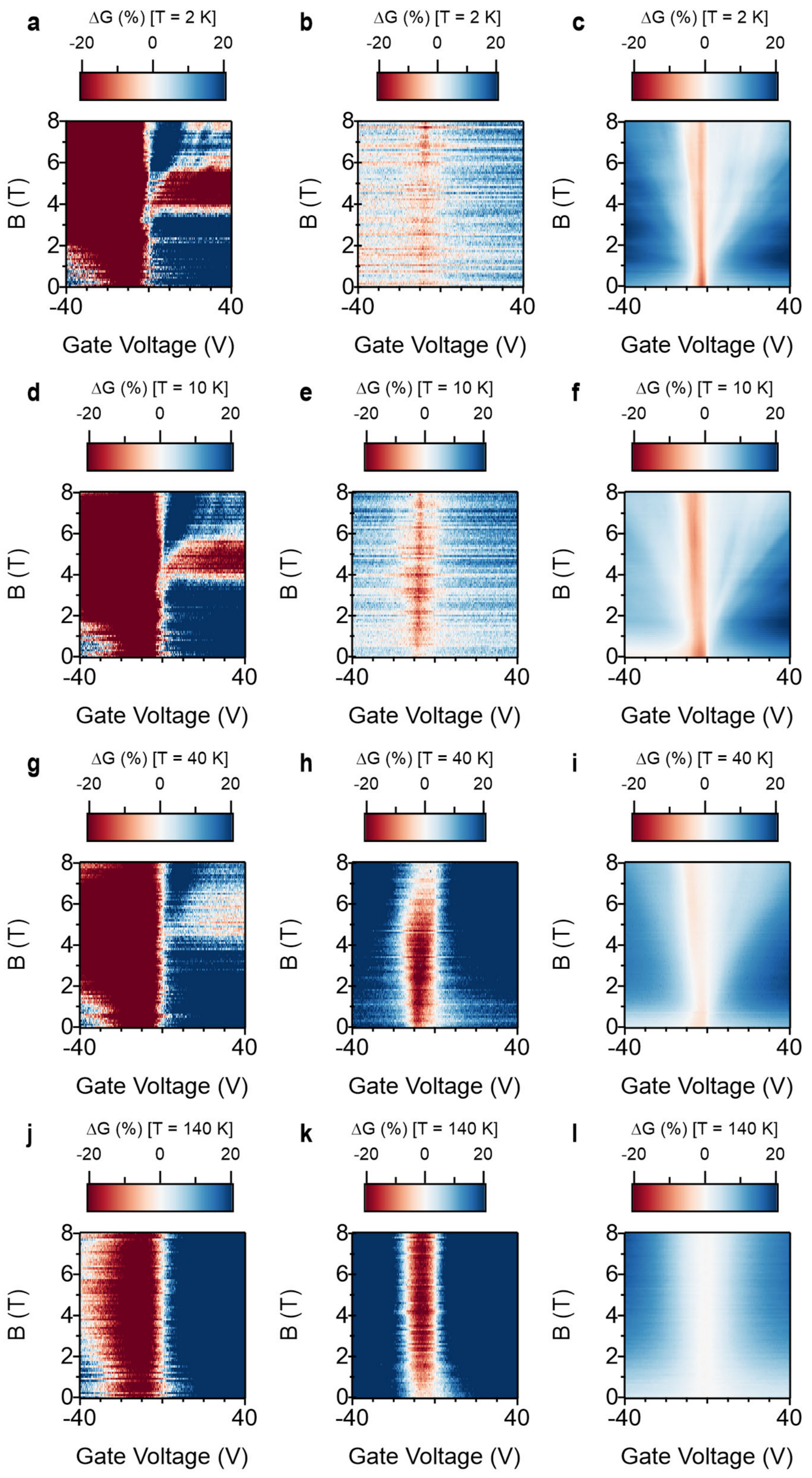

Figure S.75.- Gate and field dependence of ΔG at 2 K (a, b and c), 10 K (d, e and f), 40 K (g, h and i) and 140 K (j, k and l) for the bottom FLG contact (a, d, g and j), junction (b, e, h and k) and top FLG contact (c, f, i and l).

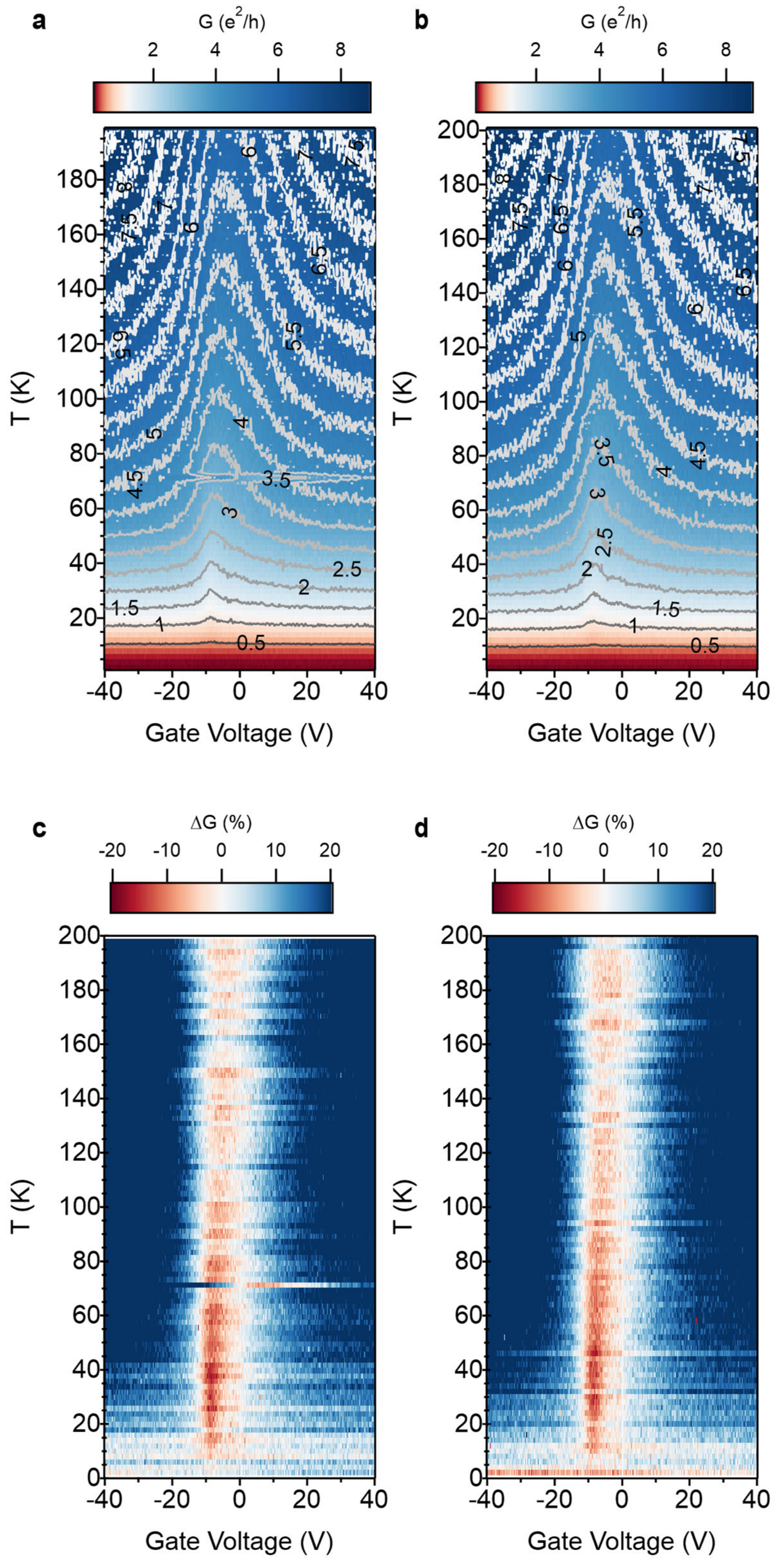

Figure S.76.- Gate and temperature dependence of the conductance (a and b) and ΔG (c and d) for the junction while cooling down (from 200 K to 2 K; a and c) and warming up (from 2 K to 200 K; b and d).

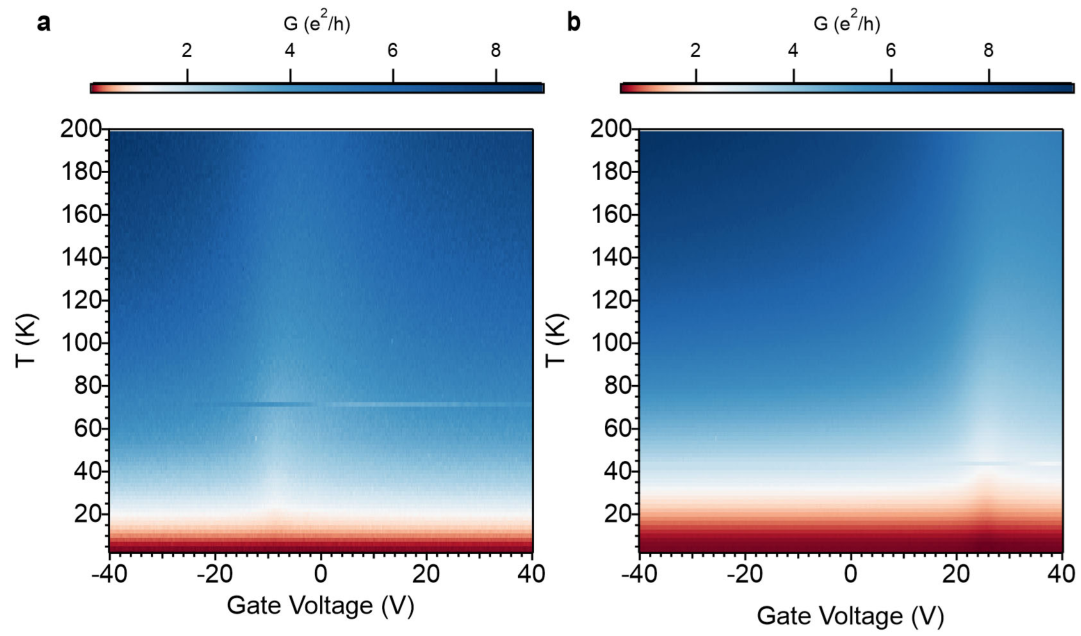

Figure S.77.- Gate and temperature dependence of the conductance of the junction while cooling down with an applied gate voltage of 0 V (a) and + 35 V (b).

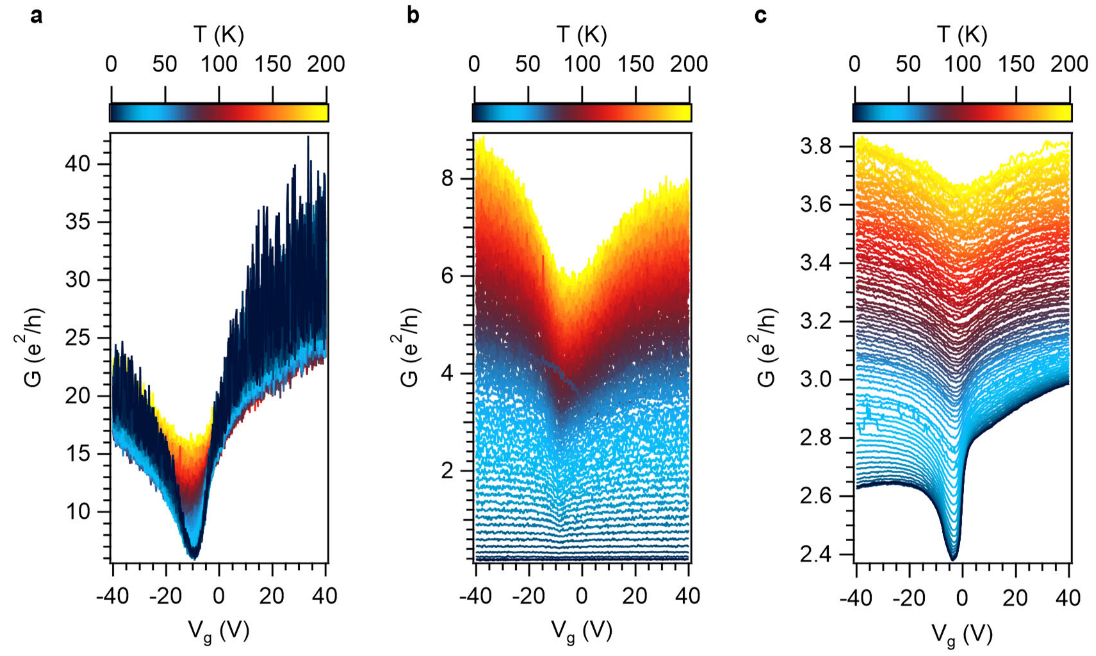

Figure S.78.- Gate dependence of the conductance at different temperatures for the bottom FLG (a), the junction (b) and the top FLG (c).

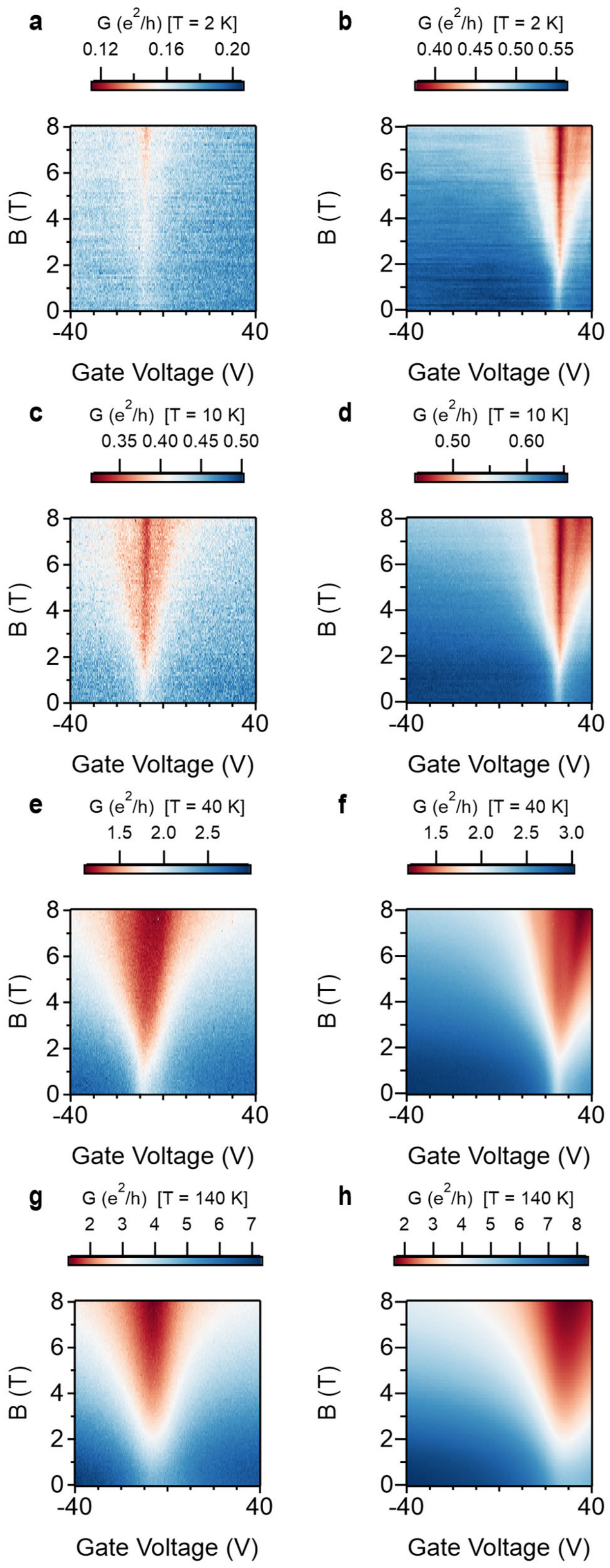

Figure S.79.- Gate and field dependence at various temperatures of the conductance of the junction while cooling down with an applied gate voltage of 0 V (a, c, e and g) and + 35 V (b, d, f and h).

4.4. Device D.

Device

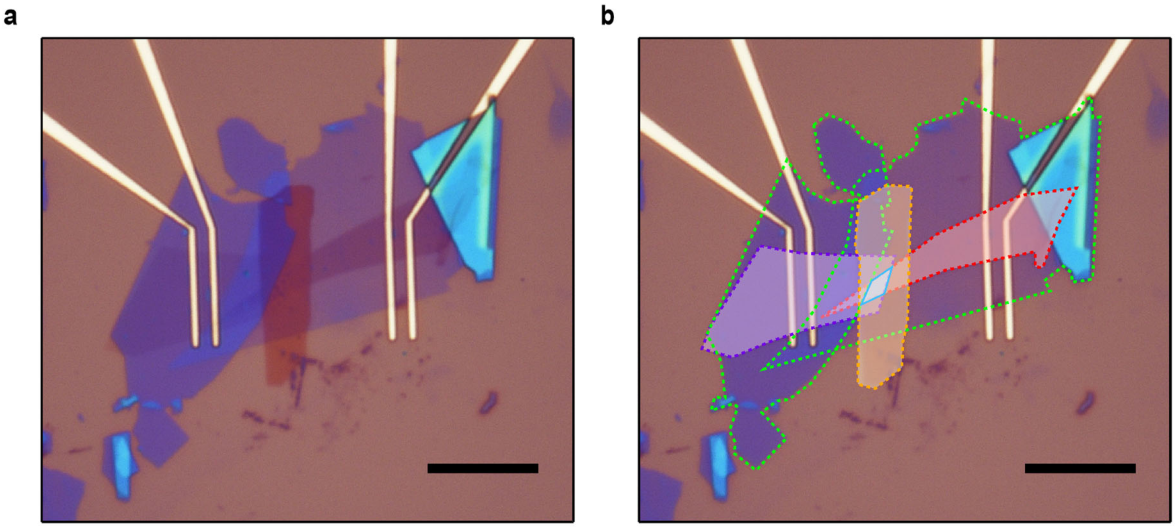

Figure S.80.- Optical picture of the device. For simplicity, the top graphite contact has been highlighted in red, the bottom graphite contact in purple and the 1T-TaS_2 barrier in orange. h-BN is marked in green. The junction (blue area) is the overlapped area formed by the three different materials. Scale bar: 10 μm.

Transport measurements.

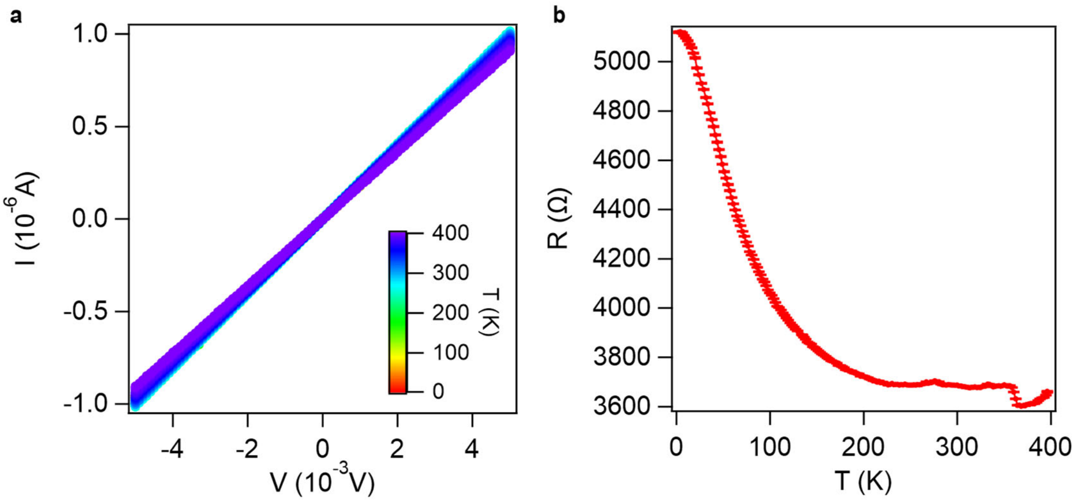

Figure S.81.- DC current versus DC voltage (left) for the junction as a function of the temperature as well as the respective fitted resistance (right).

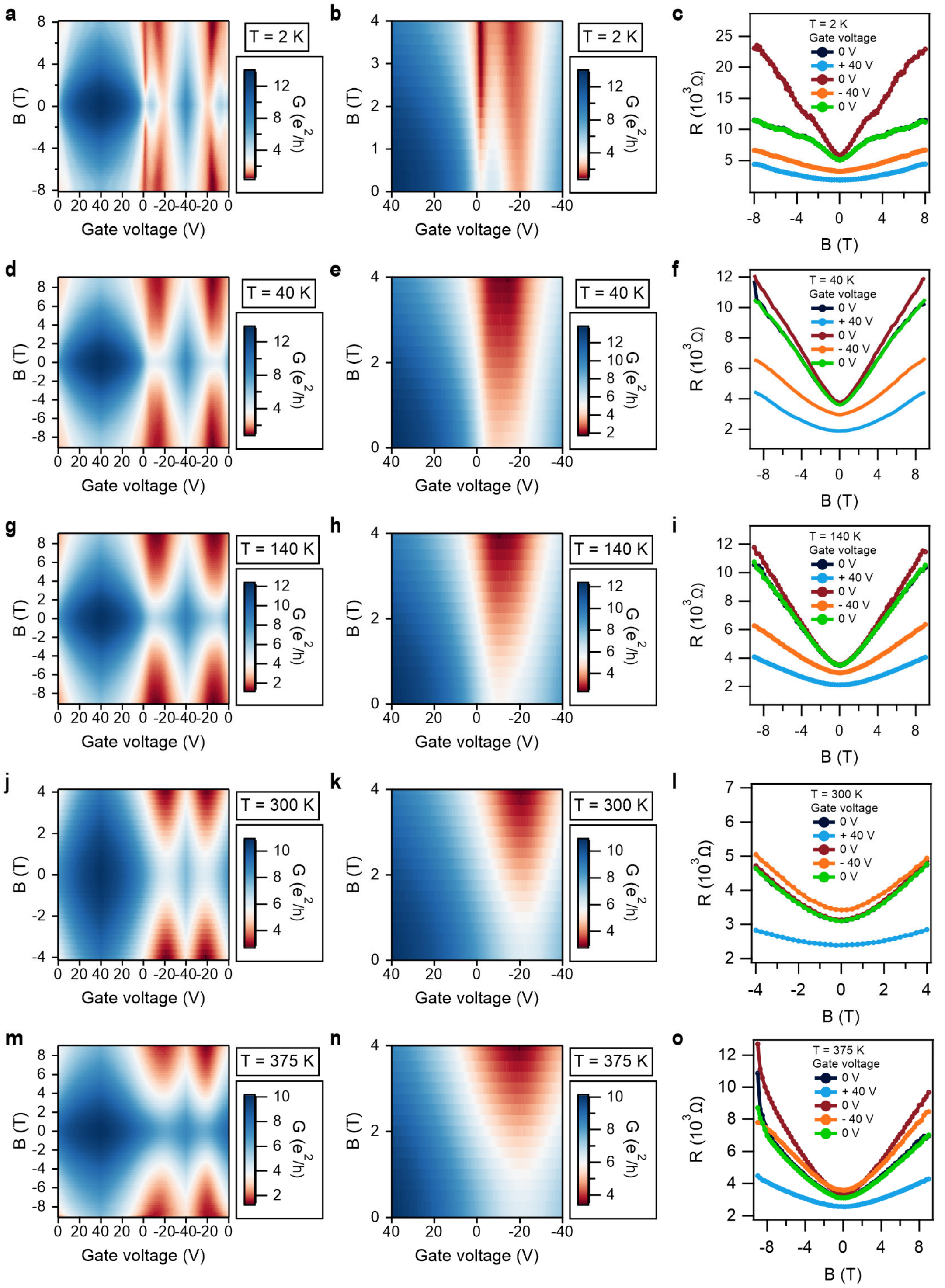

Figure S.82.- Field and gate voltage dependence of the conductance for different temperatures. It is also shown (right column) the field dependence of the resistance for different gate voltage values and temperatures.

4.5. Device E.

Device.

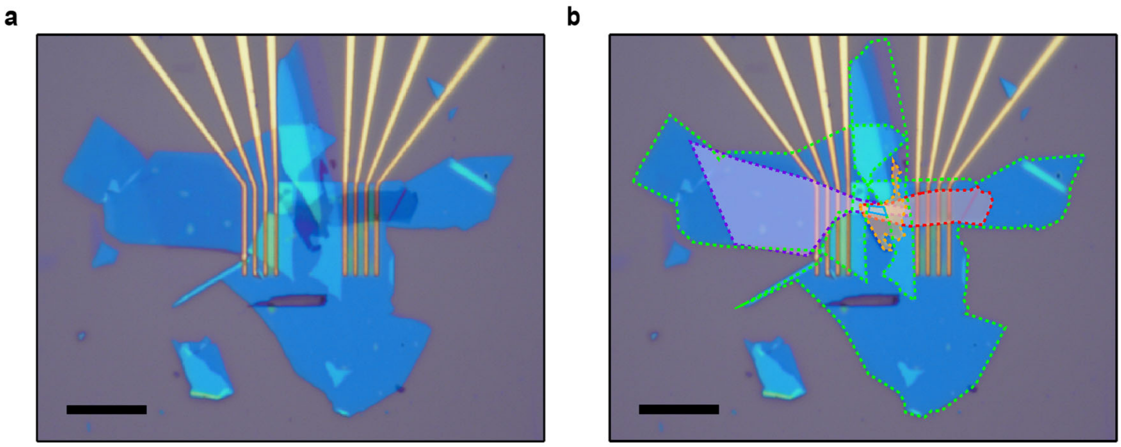

Figure S.83.- Optical picture of the device. For simplicity, the top graphite contact has been highlighted in red, the bottom graphite contact in purple and the 1T-TaS_2 barrier in orange. h-BN is marked in green. The junction (blue area) is the overlapped area formed by the three different materials. Scale bar: 10 µm.

Transport measurements.

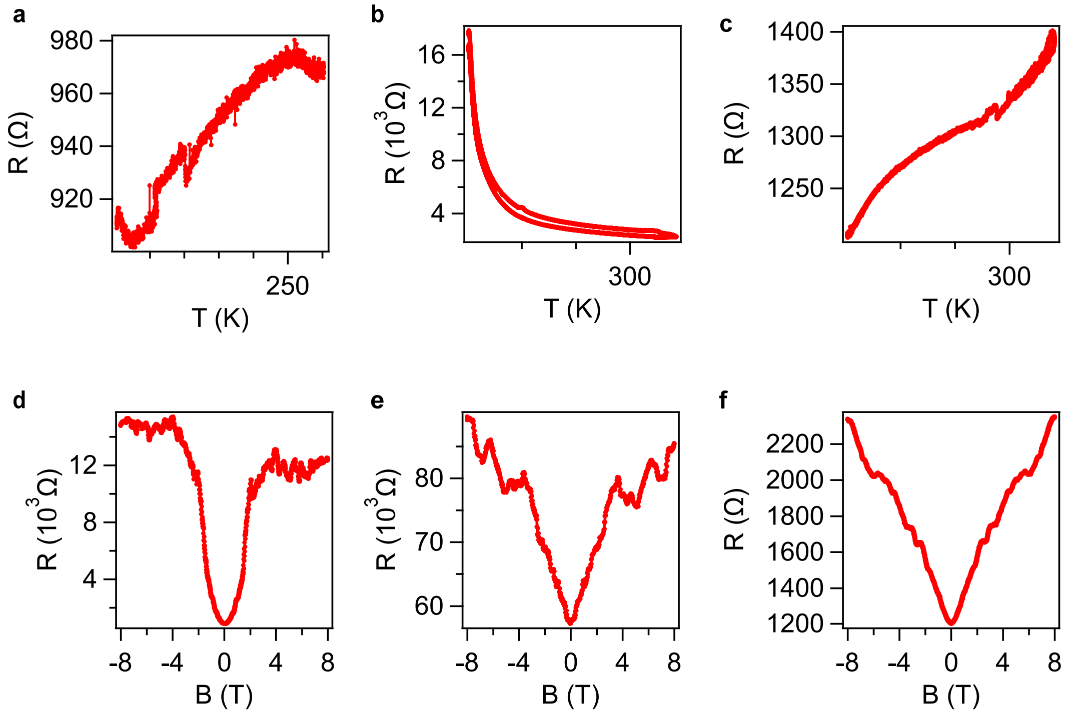

Figure S.84.- Dependence of the resistance as a function of the temperature (a, b, c) and the field (d, e, f) for the bottom graphite contact (a, d), the junction (b, e) and the top graphite contact (c, f) at 2 K.

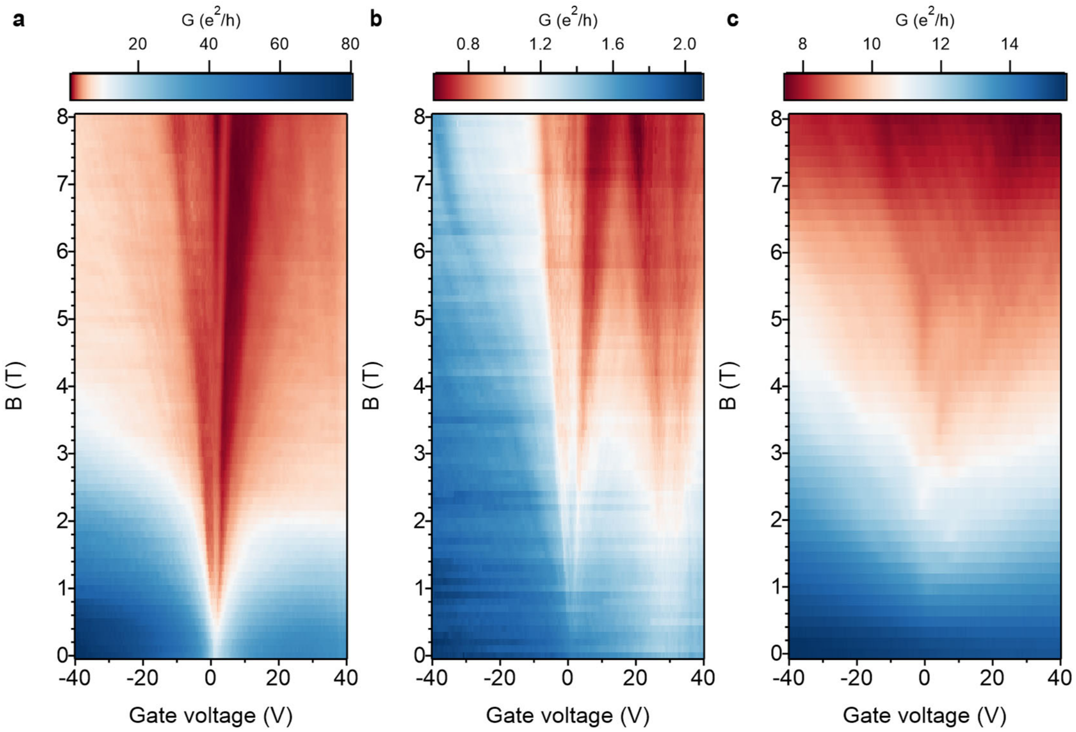

Figure S.85.- Conductance as a function of the gate voltage and the magnetic field at 2 K for a) the bottom graphite contact, b) the junction and c) the top graphite contact.

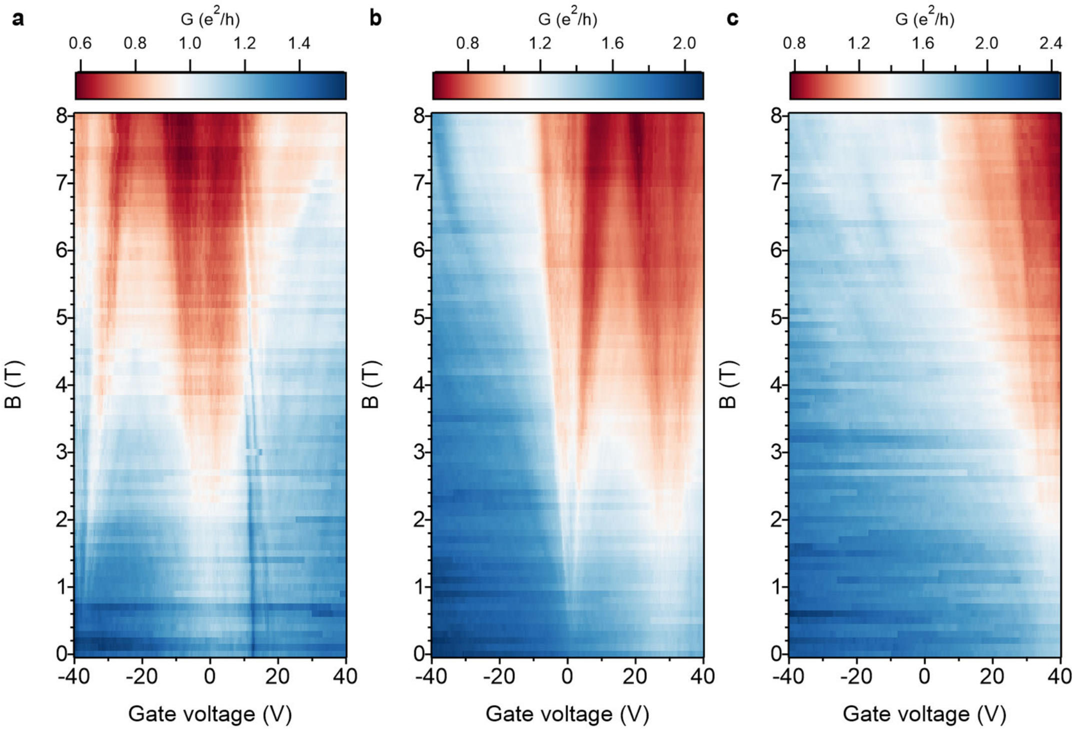

Figure S.86.- Conductance as a function of the gate voltage and the magnetic field at 2 K for the junction by cooling down from 400 K to 2 K with a fixed gate of a) – 40 V, b) 0 V and c) +40 V.

4.6. Device F.

Device.

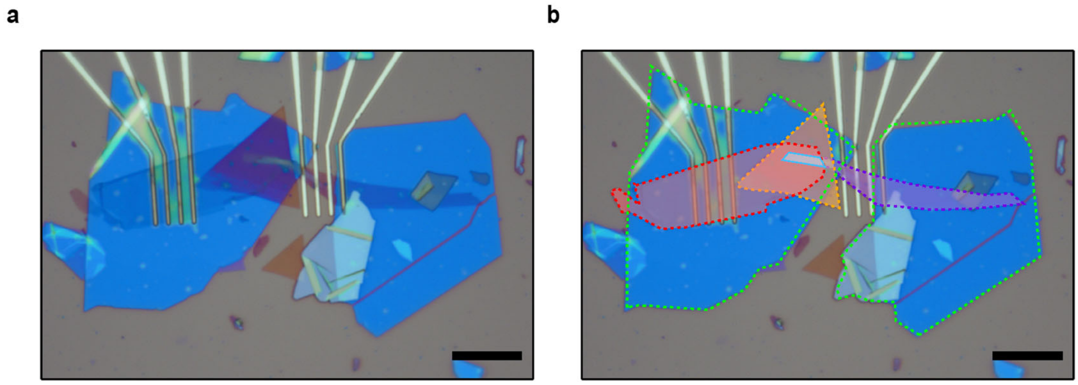

Figure S.87.- Optical picture of the device. For simplicity, the top graphite contact has been highlighted in red, the bottom graphite contact in purple and the 1T-TaS_2 barrier in orange. h-BN is marked in green. The junction (blue area) is the overlapped area formed by the three different materials. Scale bar: 10 µm.

Transport measurements.

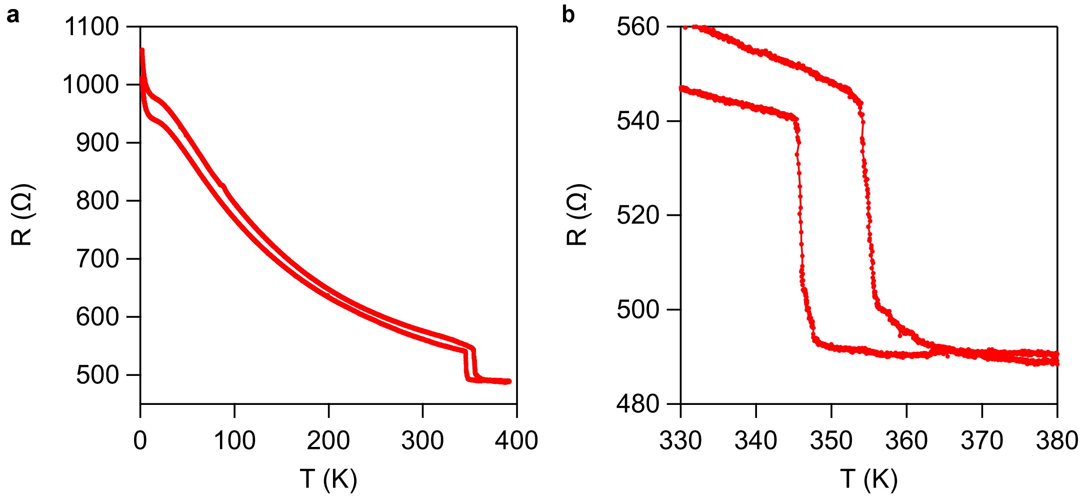

Figure S.88.- Temperature dependence of the resistance of the junction. Full temperature range (left) and detailed around the N-CDW to I-CDW transition of the 1T-TaS_2 (right).

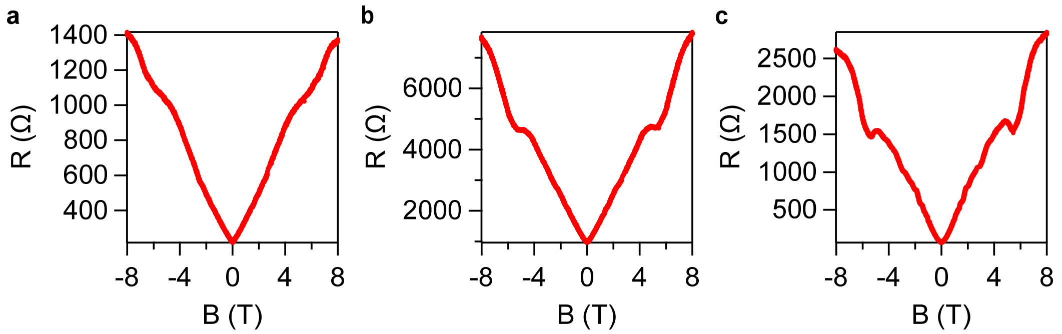

Figure S.89.- Field dependence of the resistance for both graphite contacts (a for the bottom contact; c for the top one) and for the junction (b) at 2 K.

4.7. Device G.

Device.

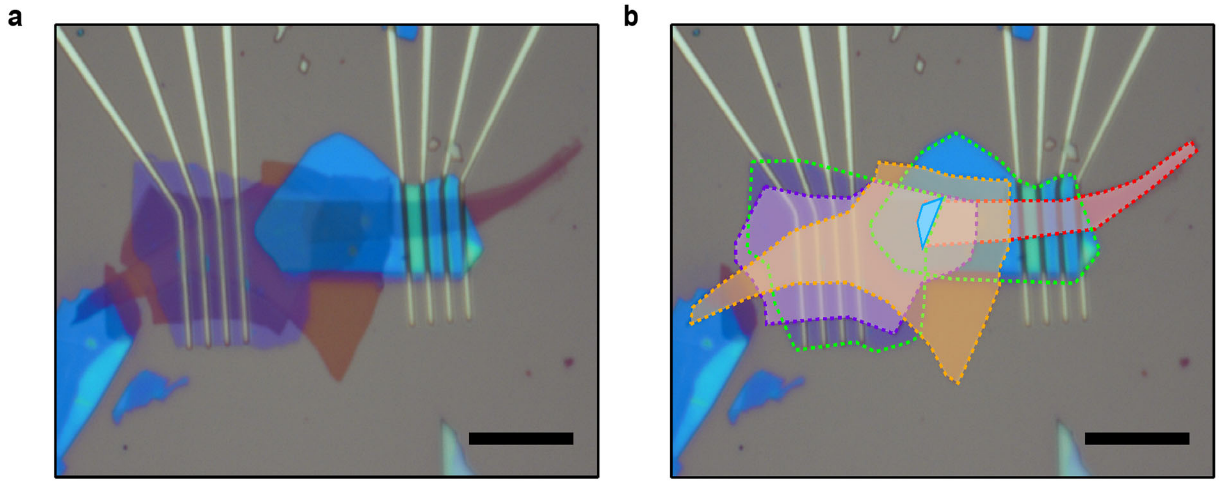

Figure S.90.- Optical picture of the device. For simplicity, the top graphite contact has been highlighted in red, the bottom graphite contact in purple and the 1T-TaS_2 barrier in orange. h-BN is marked in green. The junction (blue area) is the overlapped area formed by the three different materials. Scale bar: 10 µm.

Transport measurements.

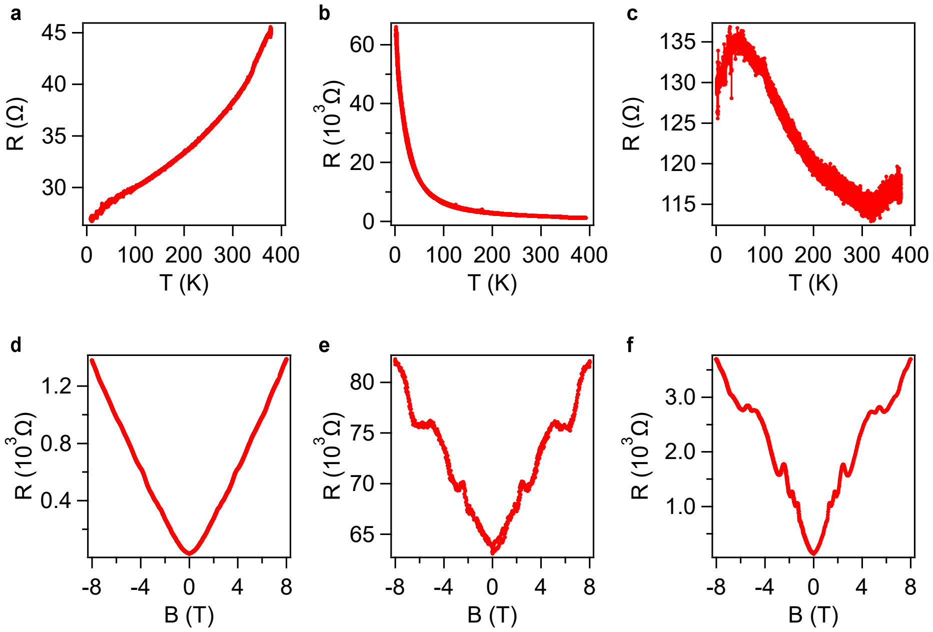

o

Figure S.91.- Dependence of the resistance as a function of the temperature (a, b, c) and the field (d, e, f) for the bottom graphite contact (a, d), the junction (b, e) and the top graphite contact (c, f) at 2 K.

4.8. Device H.

Device.

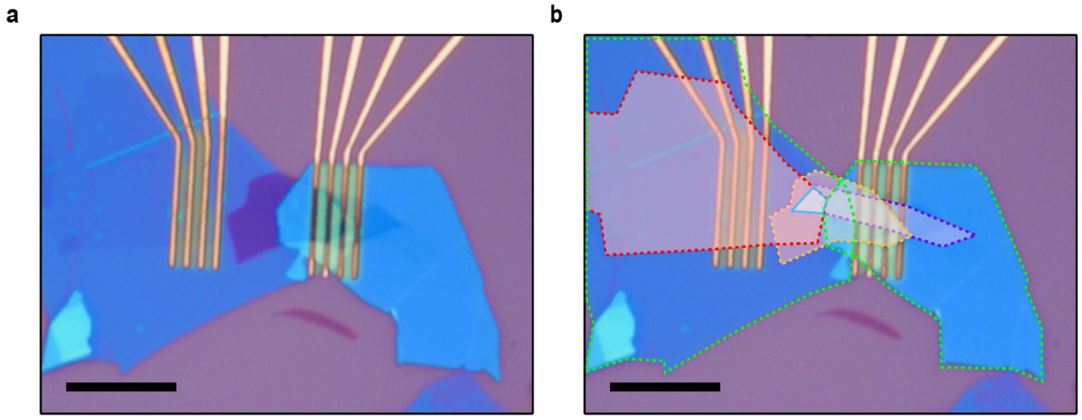

Figure S.92.- Optical picture of the device. For simplicity, the top graphite contact has been highlighted in red, the bottom graphite contact in purple and the 1T-TaS_2 barrier in orange. h-BN is marked in green. The junction (blue area) is the overlapped area formed by the three different materials. Scale bar: 10 μm.

Transport measurements.

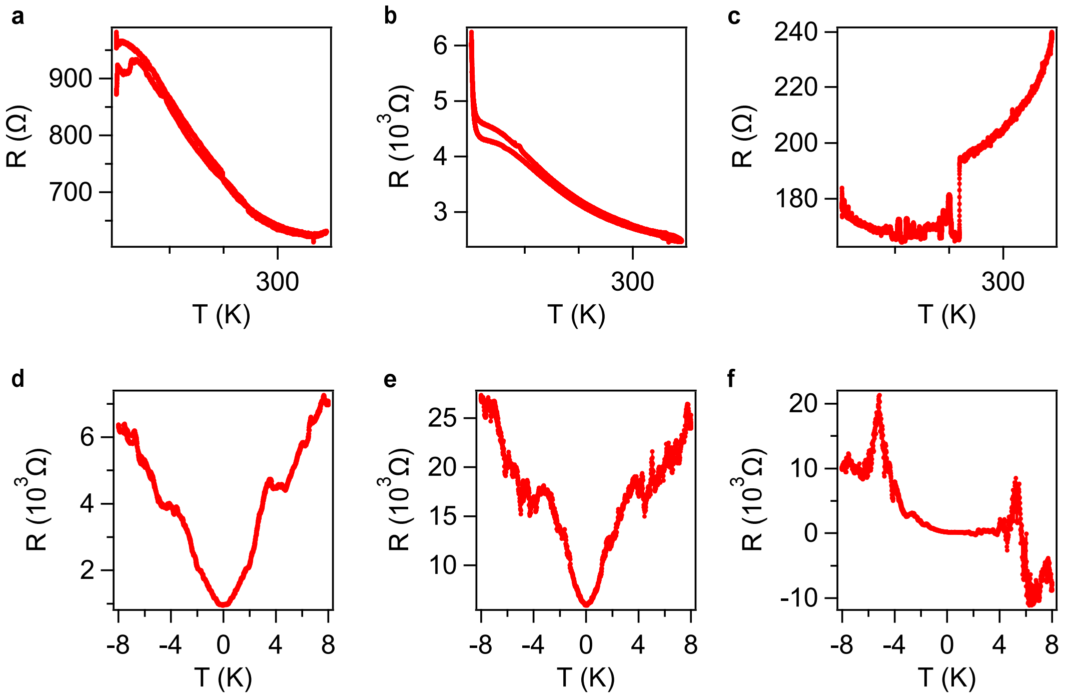

Figure S.93.- Dependence of the resistance as a function of the temperature (a, b, c) and the field (d, e, f) for the bottom graphite contact (a, d), the junction (b, e) and the top graphite contact (c, f) at 2 K.

4.9. Device I.

Device.

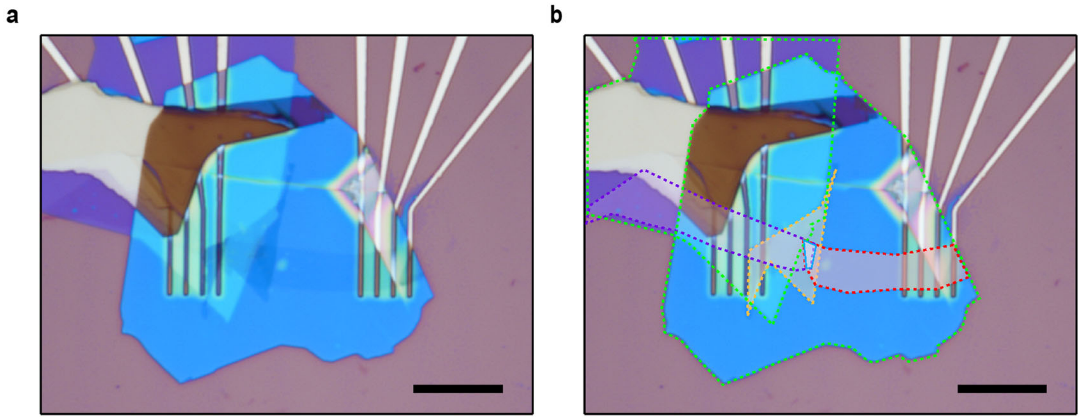

Figure S.94.- Optical picture of the device. For simplicity, the top graphite contact has been highlighted in red, the bottom graphite contact in purple and the 1T-TaS_2 barrier in orange. h-BN is marked in green. The junction (blue area) is the overlapped area formed by the three different materials. Scale bar: 10 μm.

Transport measurements.

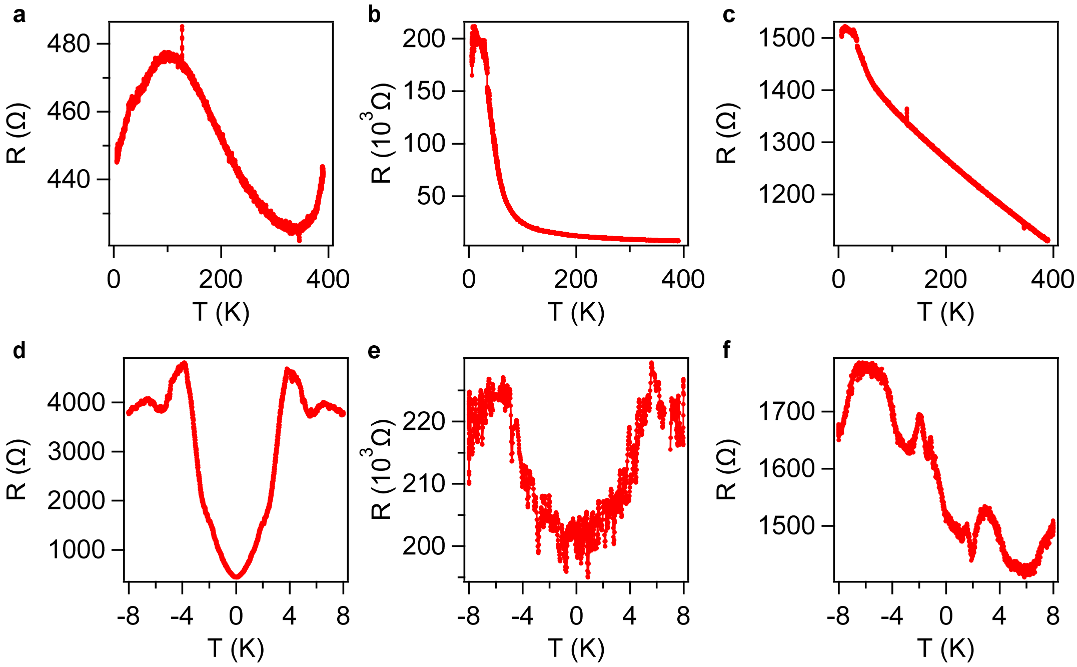

Figure S.95.- Dependence of the resistance as a function of the temperature (a, b, c) and the field (d, e, f) for the bottom graphite contact (a, d), the junction (b, e) and the top graphite contact (c, f) at 2 K.

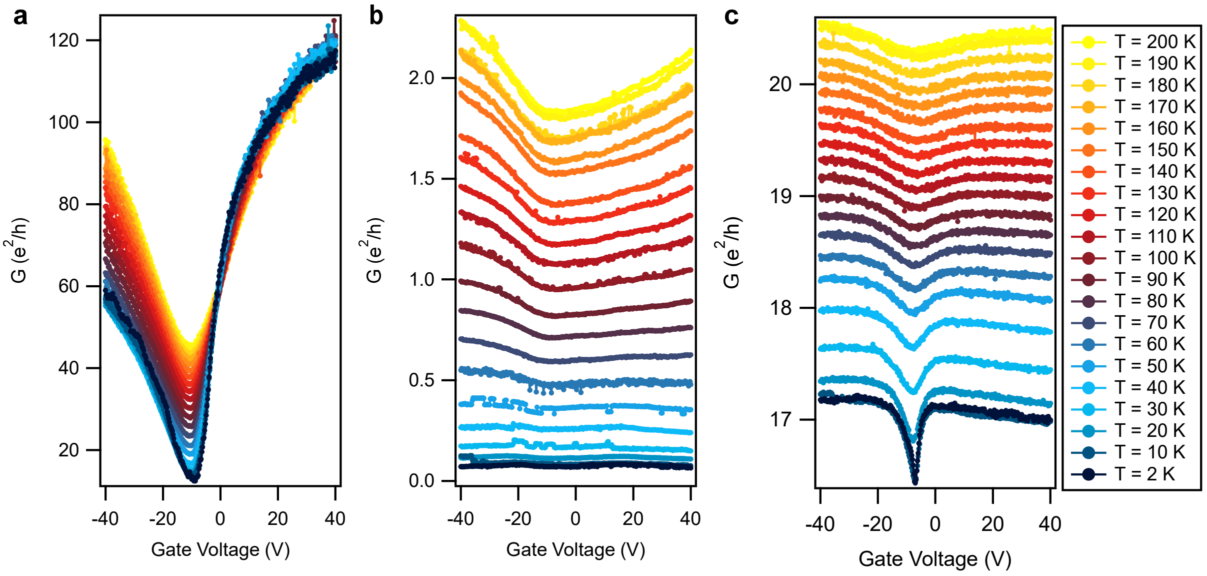

Figure S.96.- Gate dependence of the conductance at different temperatures for the bottom FLG (a), the junction (b) and the top FLG (c).

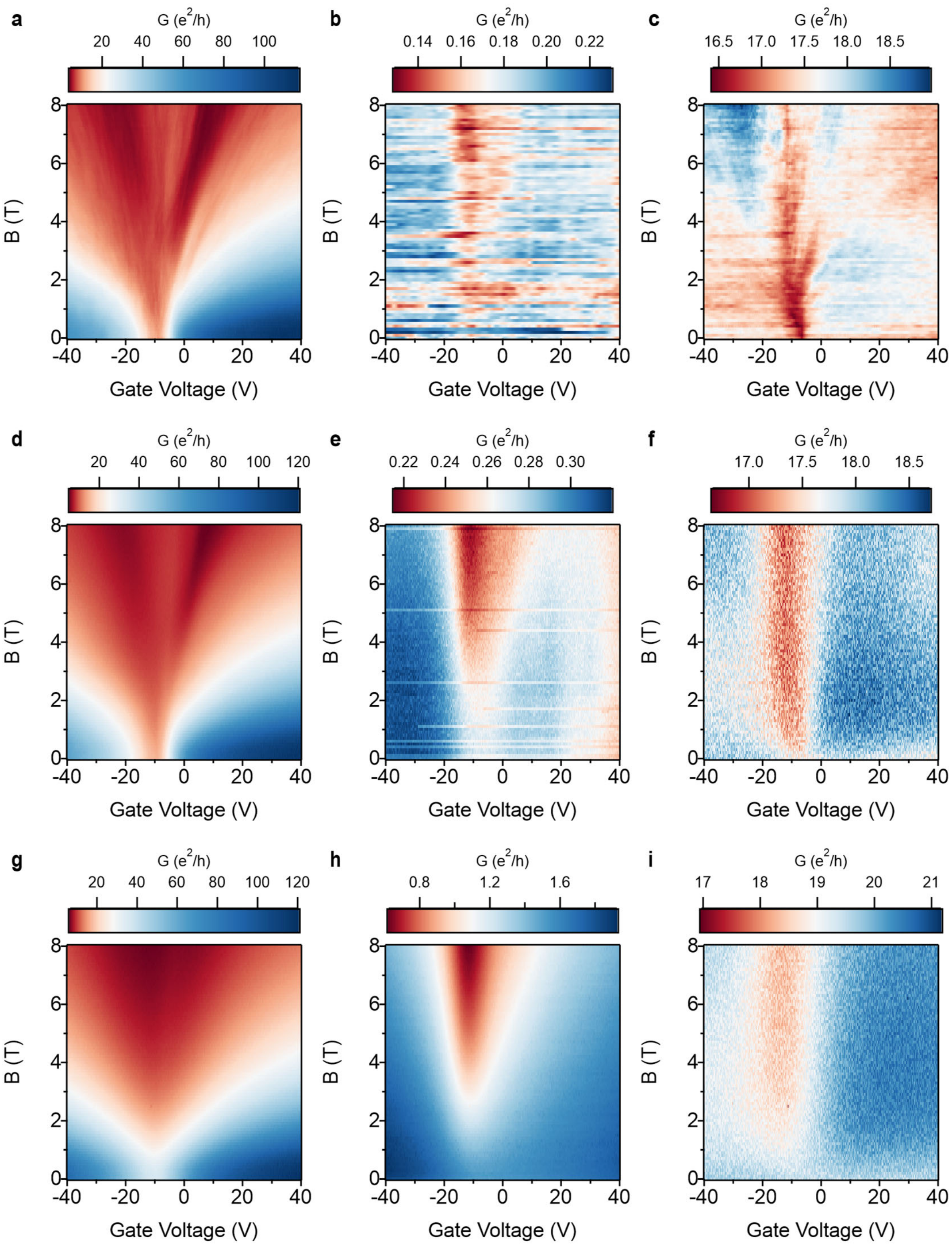

Figure S.97.- Conductance of the bottom FLG contact (a, d, g), the junction (b, e, h) and the top FLG contact (c, f, i) as a function of the gate voltage and external magnetic field for different temperatures (a, b, c for 2 K; d, e, f for 40 K and g, h, i for 140 K).

5. Scanning transmission electron microscopy images.

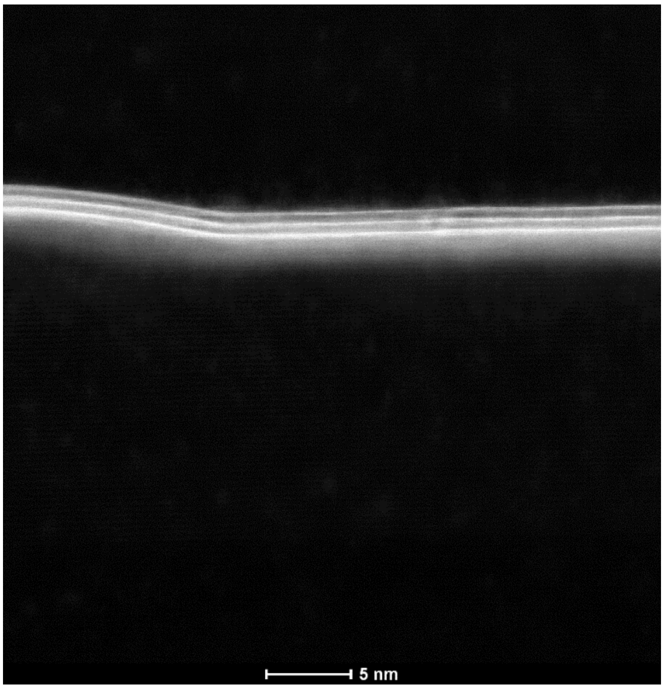

Figure S.98.- Scanning transmission electron microscopy (STEM) image of an area of the cross-sectional view of the vertical heterostructure (Device B), showing 3 layers of 1T-TaS_2.

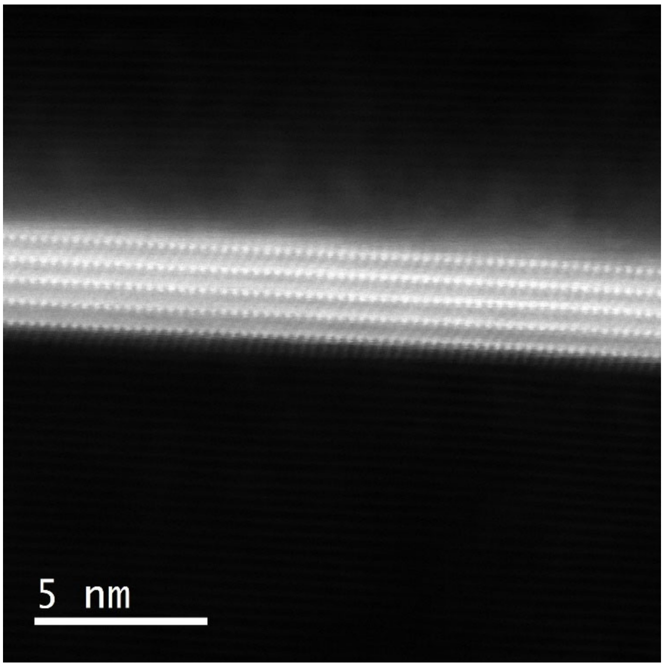

Figure S.99.- Scanning transmission electron microscopy (STEM) image of an area of the cross-sectional view of the vertical heterostructure (Device D), showing 5 layers of 1T-TaS_2.

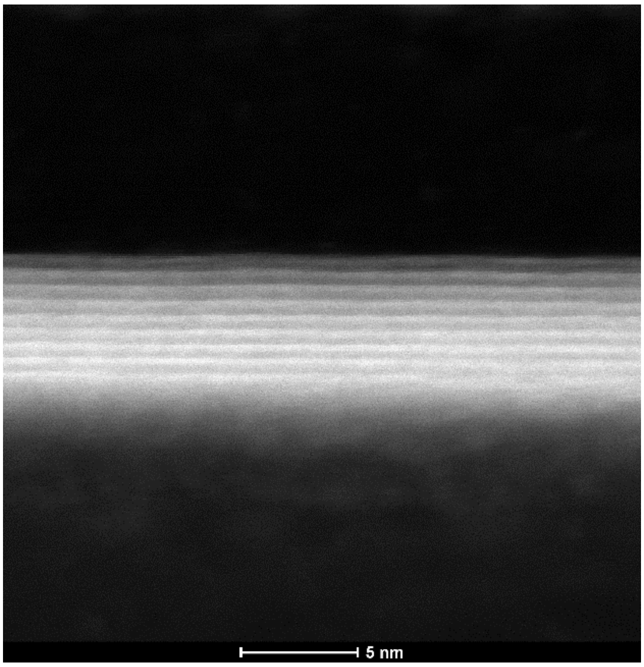

Figure S.100.- Scanning transmission electron microscopy (STEM) image of an area of the cross-sectional view of the vertical heterostructure (Device A), showing 9 layers of $1T\text{-}TaS_2$.

6. Band structure calculations.

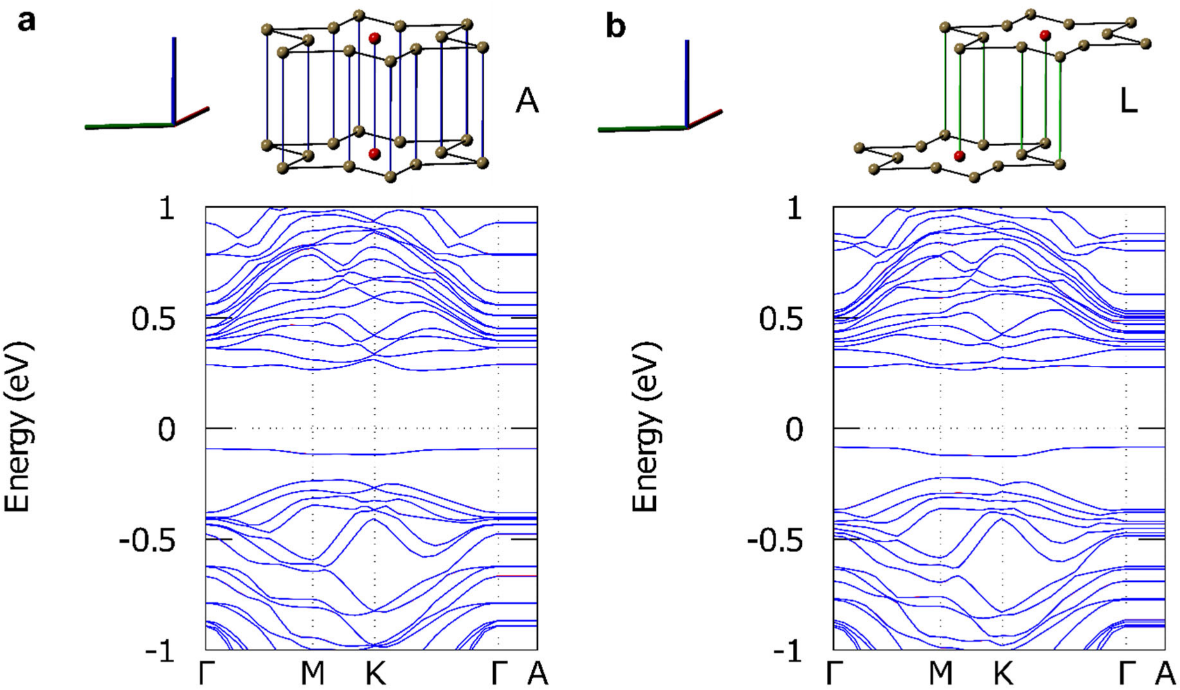

Figure S.101.- Calculated band structure for C-CDW $\sqrt{13}$ x $\sqrt{13}$ supercells of 1T-TaS_2 bilayer for (a) A stacking and (b) L stacking applying U to the d orbitals of the Ta atoms of the whole SD.

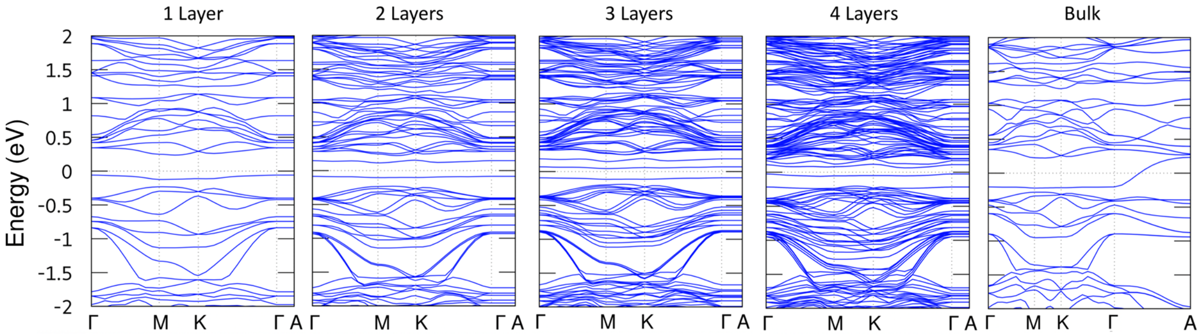

Figure S.102.- Calculated band structure for C-CDW $\sqrt{13}$ x $\sqrt{13}$ supercells of 1T-TaS_2 from monolayer to bulk applying U to the d orbitals of the central Ta atoms of the SD.

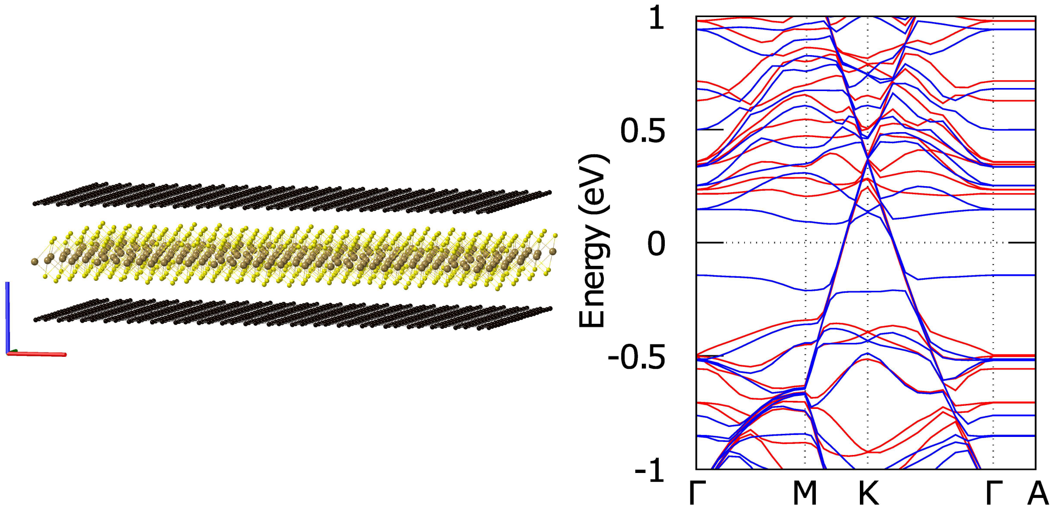

Figure S.103.- Structure and DFT+U electronic band structure of a heterostructure formed by graphene/1T-TaS_2/graphene monolayers. The red, green and blue colors of the axis correspond to the a, b, and c axis, respectively.

7. References.

(1) Castellanos-Gomez, A.; Navarro-Moratalla, E.; Mokry, G.; Quereda, J.; Pinilla-Cienfuegos, E.; Agraït, N.; van der Zant, H. S. J.; Coronado, E.; Steele, G. a.; Rubio-Bollinger, G. Fast and Reliable Identification of Atomically Thin Layers of TaSe2 Crystals. *Nano Res.* **2013**, *6* (3), 191–199. https://doi.org/10.1007/s12274-013-0295-9.

(2) Li, H.; Wu, J.; Huang, X.; Lu, G.; Yang, J.; Lu, X.; Xiong, Q.; Zhang, H. Rapid and Reliable Thickness Identification of Two-Dimensional Nanosheets Using Optical Microscopy. *ACS Nano* **2013**, *7* (11), 10344–10353. https://doi.org/10.1021/nn4047474.

(3) Huang, B.; Clark, G.; Navarro-Moratalla, E.; Klein, D. R.; Cheng, R.; Seyler, K. L.; Zhong, D.; Schmidgall, E.; McGuire, M. A.; Cobden, D. H.; Yao, W.; Xiao, D.; Jarillo-Herrero, P.; Xu, X. Layer-Dependent Ferromagnetism in a van der Waals Crystal down to the Monolayer Limit. *Nature* **2017**, *546* (7657), 270–273. https://doi.org/10.1038/nature22391.

(4) Blake, P.; Hill, E. W.; Castro Neto, A. H.; Novoselov, K. S.; Jiang, D.; Yang, R.; Booth, T. J.; Geim, A. K. Making Graphene Visible. *Appl. Phys. Lett.* **2007**, *91* (6), 063124. https://doi.org/10.1063/1.2768624.

(5) Navarro-Moratalla, E.; Island, J. O.; Mañas-Valero, S.; Pinilla-Cienfuegos, E.; Castellanos-Gomez, A.; Quereda, J.; Rubio-Bollinger, G.; Chirolli, L.; Silva-Guillén, J. A.; Agraït, N.; Steele, G. A.; Guinea, F.; van der Zant, H. S. J.; Coronado, E. Enhanced Superconductivity in Atomically Thin TaS2. *Nat. Commun.* **2016**, *7*, 11043. https://doi.org/10.1038/ncomms11043.

(6) Ayari, A.; Cobas, E.; Ogundadegbe, O.; Fuhrer, M. S. Realization and Electrical Characterization of Ultrathin Crystals of Layered Transition-Metal Dichalcogenides. *J. Appl. Phys.* **2007**, *101*, 1–5. https://doi.org/10.1063/1.2407388.

(7) Peck, E. R.; Fisher, D. J. Dispersion of Argon. *J. Opt. Soc. Am.* **1964**, *54* (11), 1362. https://doi.org/10.1364/JOSA.54.001362.

(8) Green, M. A. Self-Consistent Optical Parameters of Intrinsic Silicon at 300 K Including Temperature Coefficients. *Sol. Energy Mater. Sol. Cells* **2008**, *92* (11), 1305–1310. https://doi.org/10.1016/j.solmat.2008.06.009.

(9) Rodríguez-de Marcos, L. V.; Larruquert, J. I.; Méndez, J. A.; Aznárez, J. A. Self-Consistent Optical Constants of SiO2 and Ta2O5 Films. *Opt. Mater. Express* **2016**, *6* (11), 3622. https://doi.org/10.1364/OME.6.003622.

(10) Beal, A. R.; Hughes, H. P.; Liang, W. Y. The Reflectivity Spectra of Some Group VA Transition Metal Dichalcogenides. *J. Phys. C Solid State Phys.* **1975**, *8* (24), 4236–4234. https://doi.org/10.1088/0022-3719/8/24/015.

(11) Castellanos-Gomez, a.; Agrat, N.; Rubio-Bollinger, G. Optical Identification of Atomically Thin Dichalcogenide Crystals. *Appl. Phys. Lett.* **2010**, *96,* 2133116. https://doi.org/10.1063/1.3442495.

(12) Bing, D.; Wang, Y.; Bai, J.; Du, R.; Wu, G.; Liu, L. Optical Contrast for Identifying the Thickness of Two-Dimensional Materials. *Opt. Commun.* **2018**, *406* (March 2017), 128–138. https://doi.org/10.1016/j.optcom.2017.06.012.

(13) Casiraghi, C.; Hartschuh, A.; Lidorikis, E.; Qian, H.; Harutyunyan, H.; Gokus, T.; Novoselov, K. S.; Ferrari, A. C. Rayleigh Imaging of Graphene and Graphene Layers. *Nano Lett.* **2007**, *7* (9), 2711–2717. https://doi.org/10.1021/nl071168m.

(14) Yu, Y.; Yang, F.; Lu, X. F.; Yan, Y. J.; Cho, Y.-H.; Ma, L.; Niu, X.; Kim, S.; Son, Y.-W.; Feng, D.; Li, S.; Cheong, S.; Chen, X. H.; Zhang, Y. Gate-Tunable Phase Transitions in Thin Flakes of 1T-TaS2. *Nat. Nanotechnol.* **2015**, *10* (3), 270–276. https://doi.org/10.1038/nnano.2014.323.

(15) Yoshida, M.; Zhang, Y.; Ye, J.; Suzuki, R.; Imai, Y.; Kimura, S.; Fujiwara, A.; Iwasa, Y. Controlling Charge-Density-Wave States in Nano-Thick Crystals of 1T-TaS2. *Sci. Rep.* **2015**, *4* (1), 7302. https://doi.org/10.1038/srep07302.

(16) Martino, E.; Pisoni, A.; Ćirić, L.; Arakcheeva, A.; Berger, H.; Akrap, A.; Putzke, C.; Moll, P. J. W.; Batistić, I.; Tutiš, E.; Forró, L.; Semeniuk, K. Preferential Out-of-Plane Conduction and Quasi-One-Dimensional Electronic States in Layered 1T-TaS2. *npj 2D Mater. Appl.* **2020**, *4* (1), 7. https://doi.org/10.1038/s41699-020-0145-z.

(17) Jellinek, F. The System Tantalum-Sulfur. *J. Less-Common Met.* **1962**, *4* (1), 9–15. https://doi.org/10.1016/0022-5088(62)90053-X.

(18) Rhodes, D.; Chae, S. H.; Ribeiro-Palau, R.; Hone, J. Disorder in van der Waals Heterostructures of 2D Materials. *Nat. Mater.* **2019**, *18* (6), 541–549. https://doi.org/10.1038/s41563-019-0366-8.

(19) Kang, S. D.; Snyder, G. J. Charge-Transport Model for Conducting Polymers. *Nat. Mater.* **2017**, *16* (2), 252–257. https://doi.org/10.1038/nmat4784.

(20) Bedoya-Pinto, A.; Prima-García, H.; Casanova, F.; Coronado, E.; Hueso, L. E. Spin-Polarized Hopping Transport in Magnetically Tunable Rare-Earth Quinolines. *Adv. Electron. Mater.* **2015**, *1* (6), 1500065. https://doi.org/10.1002/aelm.201500065.

(21) Peng, S.; Jin, Z.; Yao, Y.; Li, L.; Zhang, D.; Shi, J.; Huang, X.; Niu, J.; Zhang, Y.; Yu, G. Metal-Contact-Induced Transition of Electrical Transport in Monolayer MoS 2 : From Thermally Activated to Variable-Range Hopping. *Adv. Electron. Mater.* **2019**, *5* (7), 1900042. https://doi.org/10.1002/aelm.201900042.
